# Synthesis of dinucleoside acylphosphonites by phosphonodiamidite chemistry and investigation of phosphorus epimerization

**DOI:** 10.3762/bjoc.11.19

**Published:** 2015-01-30

**Authors:** William H Hersh

**Affiliations:** 1 Department of Chemistry and Biochemistry, Queens College and the Graduate Center of the City University of New York, Queens, NY 11367-1597, USA

**Keywords:** acylphosphine, acyl phosphite, chiral, DFT, NMR, nucleic acids, oligonucleotides

## Abstract

The reaction of the diamidite, (iPr_2_N)_2_PH, with acyl chlorides proceeds with the loss of HCl to give the corresponding acyl diamidites, RC(O)P(N(iPr)_2_)_2_ (R = Me (**7**), Ph (**9**)), without the intervention of sodium to give a phosphorus anion. The structure of **9** was confirmed by single-crystal X-ray diffraction. The coupling of the diamidites **7** and **9** with 5′-*O*-DMTr-thymidine was carried out with *N*-methylimidazolium triflate as the activator to give the monoamidites 3′-*O*-(P(N(iPr)_2_)C(O)R)-5′-*O*-DMTr-thymidine, and further coupling with 3′-*O*-(*tert*-butyldimethylsilyl)thymidine was carried out with activation by pyridinium trifluoroacetate/*N*-methylimidazole. The new dinucleoside acylphosphonites could be further oxidized, hydrolyzed to the *H*-phosphonates, and sulfurized to give the known mixture of diastereomeric phosphorothioates. The goal of this work was the measurement of the barrier to inversion of the acylphosphonites, which was expected to be low by analogy to the low barrier found in acylphosphines. However, the barrier was found to be high as no epimerization was detected up to 150 °C, and consistent with this, density functional theory calculations give an inversion barrier of over 40 kcal/mol.

## Introduction

Antisense oligonucleotides, which are modified oligonucleotides that bind to mRNA in order to inhibit translation to proteins, have been intensively investigated for decades [1–2]. Modifications of native DNA or RNA are required to permit pharmaceutical use, for example, for conferring nuclease resistance, enhanced binding to mRNA, and recruitment of RNase H. A selection of recent publications [3–7] illustrates the changes that have occurred since the first antisense drug was approved in 1998 [8]. However, despite ongoing clinical trials, only recently has a second antisense drug [3] been approved [9].

Most (albeit not all) antisense reagents, including the two approved drugs, are phosphorothioates, in which one of the terminal oxygen atoms on the phosphodiester internucleotide linker is replaced by sulfur. A potential issue with phosphorothioates is the creation of a new stereocenter at every phosphorus linker. This results in the formation of an enormous number of diastereomers that may have different properties, such as nuclease stability and RNase H interactions. Some [5,10] but not all [11–12] of the non-phosphorothioate antisense reagents avoid the diastereomer problem, but the extensive study of phosphorothioates is reason enough to investigate the synthesis of P-chiral phosphorothioates [13–19]. However, none of the reported methods seem to allow for routine, high-yield synthesis with high stereocontrol [20–23]. The extension to phosphorothioate RNA for applications of RNA interference has also been described [24].

We recently reported the synthesis of a number of chiral disulfide sulfurizing reagents [25] and the results of the sulfurization of phosphite triesters in order to look for stereoselectivity [26]. In order to change such a method into a practical synthesis route for P-chiral phosphorothioates, a trivalent dinucleoside phosphorus moiety is required that undergoes rapid epimerization under the sulfurization conditions – that is, the goal was to carry out a dynamic kinetic resolution with formation of a single epimeric phosphorothioate.

**Figure 1:**
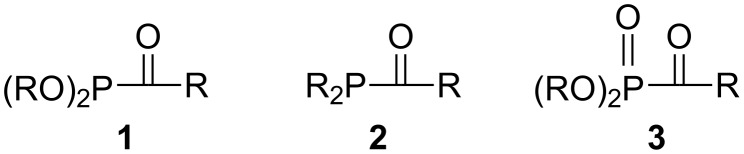
Acyl phosphorus compounds.

In order to carry out the required dynamic kinetic resolution, it was hypothesized that acylphosphonites **1** might undergo rapid epimerization by comparison to their known acylphosphine analogs **2** (Figure 1). Mislow previously showed that trialkyl and triarylphosphines slowly undergo epimerization only at temperatures above approximately 130 °C, with activation barriers near 33 kcal/mol [27], but that acylphosphines **2** undergo epimerization much more rapidly [28]. Although acylphosphines are pyramidal, the analogy to amides suggests that the planar transition state for epimerization was greatly stabilized. Literature data from several groups suggests that the inversion barrier is decreased to below 20 kcal/mol [28–30]. In order to extend the comparison from phosphines to phosphites, we recently showed that dinucleoside phosphite triesters, like phosphines, epimerize slowly – in this case at 150 °C with an inversion barrier of 33 kcal/mol [31]. Since the replacement of an alkyl or aryl group in phosphines by an acyl group lowered the epimerization barrier by >13 kcal/mol, it seemed reasonable to propose the same could happen by a similar replacement of an alkoxy group in phosphite triesters.

Although the required acylphosphonites are essentially unknown, acylphosphonates **3** (Figure 1) have been extensively studied over the past few decades [32], both for their synthetic utility [33–37] and as compounds of biological interest [38–39]. They have also been used for phosphorothioate synthesis, as will be described shortly. The straightforward route for the synthesis of **3** involves the Arbuzov reaction of a trialkyl phosphite ester with an acid chloride [40], necessarily giving the dialkyl acylphosphonate with identical alkoxy groups. Thus, no comparable synthesis of **1** could be carried out since the dinucleoside acylphosphonite would require different nucleoside moieties.

**Scheme 1:**
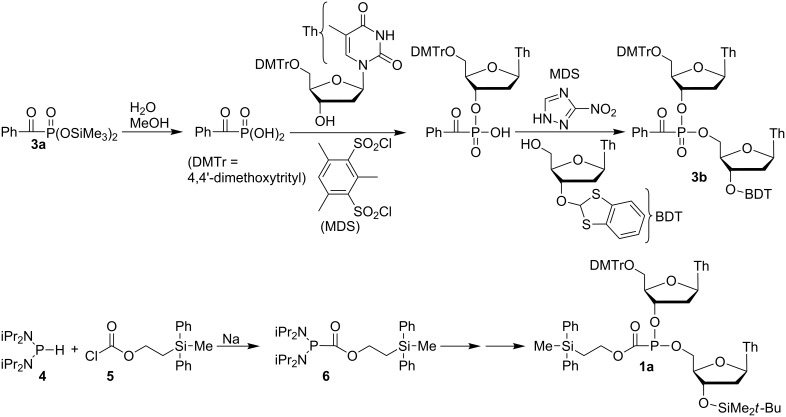
Synthesis of a dinucleoside acylphosphonate (**3b**) and a formate diester (**1a**).

One example can be found in the literature of a non-Arbuzov route leading to the dinucleoside analog of **3** where as reported by Hata et al., **3a** [41] gives **3b** via sequential coupling [42–43] (Scheme 1). While the deoxygenation of phosphine oxides is known [44–45], deoxygenation of the functionally much more complex phosphonate **3b** to the dinucleoside analog of **1** was not attempted. Only one example of an acylphosphonite analog of **1** was found in the literature, namely, a dinucleoside formate reported by Caruthers et al. [46]. As shown in Scheme 1, the reaction of *H*-phosphonodiamidite **4** with chloroformate **5** gave acyl diamidite **6**, which by following standard nucleoside coupling protocols gave the formate phosphonite **1a**. The initial P–C bond formation yielding **6** was odd, since although the addition of sodium was intended to give the phosphorus anion (iPr_2_N)_2_P^−^ from **4**, the authors noted that there was in fact no evidence of any reaction of **4** with sodium. The phosphonite **1a** was reported on the basis of ^31^P NMR analysis to exist as a mixture of two diastereomers, but the possibility of epimerization seems not to have been considered. Instead, **1a** was immediately oxidized to give the phosphonoformate dinucleoside.

In this paper we report: (1) a more general synthesis of acyl diamidites, which most likely accounts for Caruthers’ results, including the X-ray crystal structure of the benzoyl analog, (2) the diamidite conversion to dinucleoside acylphosphonites, (3) conversion of these compounds to phosphorothioates, (4) evidence that acylphosphonites, unlike their phosphine analogs, do not undergo facile inversion, and (5) confirmation of this observation by density functional theory (DFT) calculations.

## Results and Discussion

**Synthesis of acylphosphonodiamidites.** Initially we attempted to deprotonate *H*-phosphonodiamidite **4** with butyllithium, but in all cases, the major reaction product with acetic anhydride was simply the starting material. However, a minor product with a peak in the ^31^P NMR spectrum near 63 ppm was observed that was comparable to that exhibited by **6** at 52 ppm. We repeated Caruthers’ method [46], which involved a two hour reflux of **4** with sodium, and similarly detected no visible disappearance of the sodium. No reaction of this mixture with acetic anhydride was detected, beyond the occasional appearance of the 63 ppm peak. Removal of the solvent from the **4**/Na mixture, followed by reaction of the resultant crude material with acetyl chloride, resulted in variable amounts of the 63 ppm peak.

**Scheme 2:**
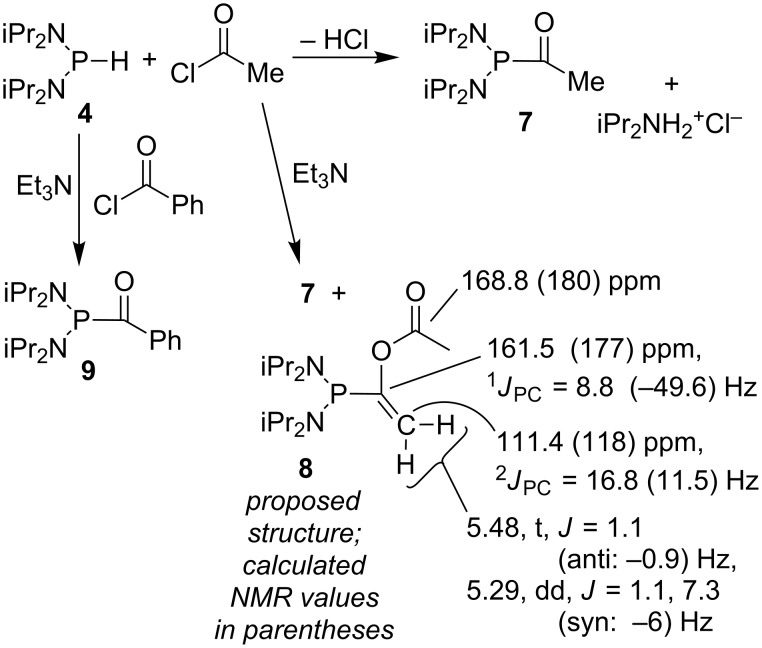
Reaction of an *H*-phosphonodiamidite with acid chlorides.

Since there was no evidence that deprotonation of **4** gave rise to the product, the direct reaction of **4** with acetyl chloride in the absence of sodium was examined (Scheme 2). Surprisingly, an immediate reaction occurred with acetyl chloride, giving a yellow-orange solution accompanied by copious production of what was assumed to be gaseous HCl. The hexane-soluble product (**7**, Scheme 2) could be isolated as an orange oil exhibiting a single peak in the ^31^P NMR spectrum at 63 ppm, but only in 64% yield. Variable amounts of iPr_2_NH_2_^+^Cl^−^ also formed, which presumably accounted for the low yield. The characterization included a doublet for the carbonyl methyl group in both the ^1^H and ^13^C NMR spectra (^1^H: 2.27 ppm, ^3^*J*_PH_ = 8.8 Hz; ^13^C: 30.7 ppm, ^2^*J*_PC_ = 49.7 Hz). However, a peak for the carbonyl carbon could not be found nor could any literature precedent be found. A DFT-NMR calculation using Gaussian [47] was carried out (see Supporting Information File 1 for details), with the surprising result that the carbonyl carbon peak was expected at 242 ppm – that is, not at all analogous to an amide chemical shift. Expansion of the standard sweep width beyond 220 ppm indeed led to observation of the missing carbon peak, at 227.9 ppm. The signal was observed as a doublet with ^1^*J*_PC_ = 22.4 Hz, in poor agreement with the calculated value of −60 Hz, although both CH_3_ calculations were in remarkably good agreement (^13^C: 31 ppm, ^2^*J*_PC_ = 54 Hz). Investigation of the poor agreement with the one-bond coupling constant has not been attempted, but the cause might be a phosphorus lone pair/carbonyl dihedral angle effect in which the true minimum energy conformation has not been found [48–50]. Once purified, **7** exhibited a strong carbonyl peak in the infrared spectrum at 1654 cm^−1^, a surprisingly low frequency considering the downfield ^13^C chemical shift. This inconsistency is perhaps due to an effect caused by the electron-donor diisopropylamide groups.

A rational approach to prevent the formation of the HCl and iPr_2_NH_2_^+^Cl^−^ that accompany the formation of **7** would involve the addition of a base. However, the use of Et_3_N gave a 43:57 mixture of **7** and a new byproduct, **8**, with a ^31^P NMR peak at 49.7 ppm. Although efforts to isolate **8** have been unsuccessful, the proposed structure (Scheme 2; see Supporting Information File 1 for details) is consistent both with formation by base-induced enolization followed by trapping with acetyl chloride, and with the ^1^H, ^13^C, and DEPT NMR spectra. For instance, the alkene-like hydrogen signals at 5.48 and 5.29 ppm have a 1.1 Hz coupling constant, which is typical of geminal alkylidene hydrogen atoms. The difference between the 1.1 and 7.3 Hz phosphorus–hydrogen coupling constants is plausible for a vinylphosphine, although the values themselves are low [48]. The NMR spectrum calculated using Gaussian was also in good agreement with experimental observations (Scheme 2; also, ^31^P NMR: observed 50 ppm, calculated 52 ppm, and for comparison, **7**: observed 64 ppm, calculated 70 ppm), although once again, the calculated one-bond coupling constant is in poor agreement.

The reaction of **4** with benzoyl chloride yielded a similar reaction, namely, copious bubbling of gaseous HCl with formation of a red-orange solution. In this case, unlike that with acetyl chloride, two peaks were observed in the ^31^P NMR spectrum, one of which appeared to be due to the desired product, **9**, while the other was far downfield at 121 ppm, perhaps due to Ph(CO)P(Cl)N(iPr)_2_. The use of Et_3_N resulted in a clean reaction presumably since no enolization is possible, leading to **9** in 89% yield after hexane extraction. A carbonyl peak was observed in the ^13^C NMR spectrum, similar to that of **7**, as a doublet at 220.9 ppm (^1^*J*_PC_ = 23.7 Hz), in addition to a strong carbonyl band in the infrared spectrum at 1631 cm^−1^.

**Figure 2:**
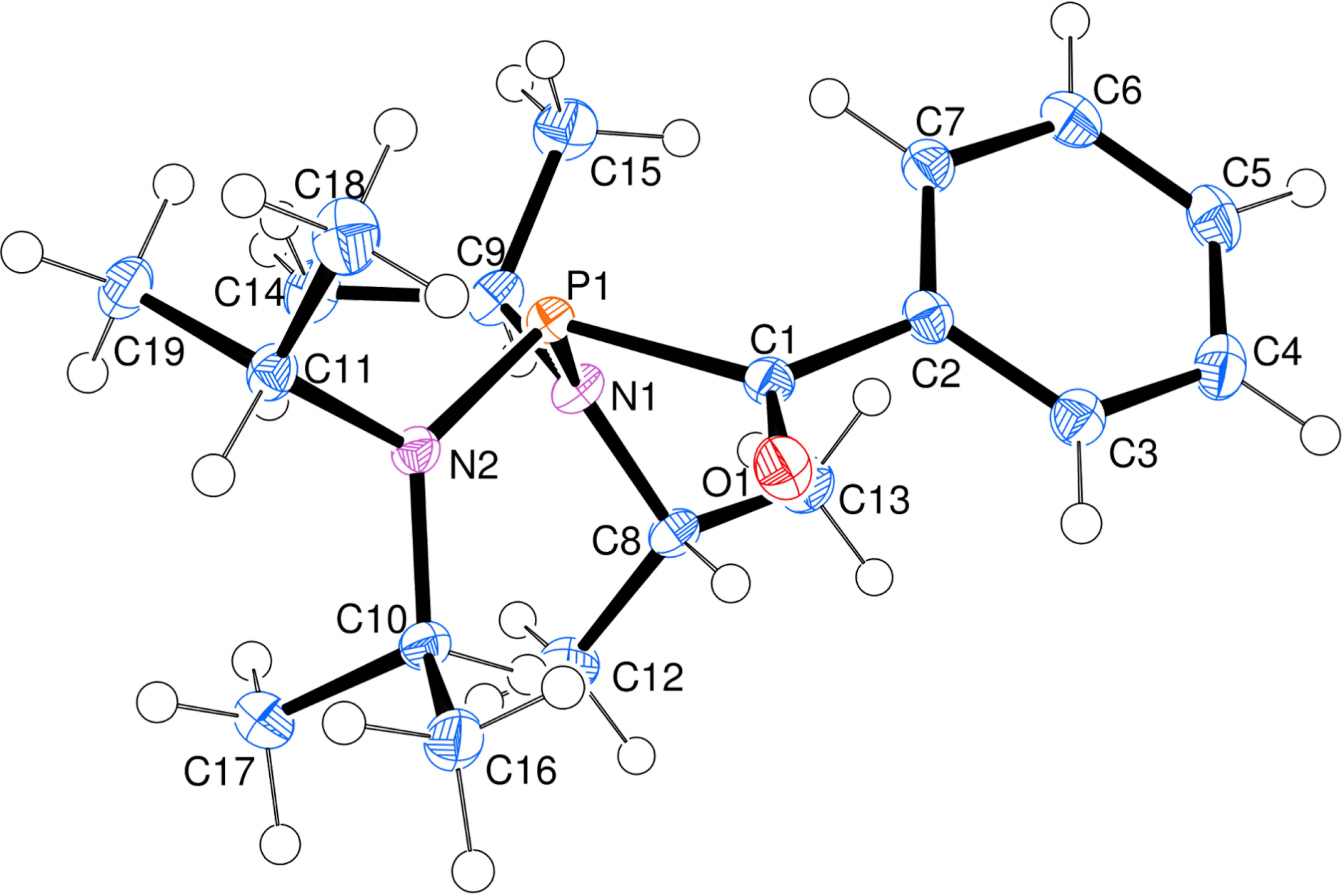
ORTEP [52] drawing of **9**. Selected distances (Å) and angles (°): P–N1 1.687(1), P–N2 1.679(1), P–C1 1.879(1), C1–O 1.221(1), C1–C2 1.495(1), C1–P–N1 99.90(5), C1–P–N2 100.59(5), P–C1–O 120.23(8), P–C1–C2 119.78(8), C2–C1–O 119.82(9).

**X-ray crystal structure of 9.** Large crystals of **9** could be obtained from hexane, allowing confirmation of the structure by single-crystal X-ray diffraction [51]. An ORTEP drawing is shown in Figure 2, illustrating the steric crowding around phosphorus due to the diisopropylamino groups. One result is that the amines are essentially planar, where the sum of the angles about each nitrogen atom is 360.0(1)°. However, the phosphorus, as expected, is pyramidal with an N–P–N angle of 112.51(5)° and average N–P–C angles of 100.2°. The dihedral angle between the carbonyl and the putative phosphorus lone pair is 108°, that is, only 18° from the angle required for the lone pair and the carbonyl p-orbital to be parallel to each other. The low CO stretching frequency in the infrared spectrum at 1631 cm^−1^ is consistent with the overlap of the carbonyl and lone pair orbitals. However, as noted above, this does not result in an amide-like ^13^C chemical shift.

**Scheme 3:**
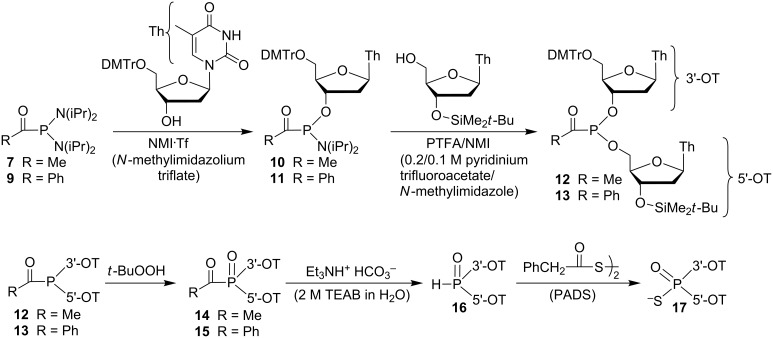
Synthesis of dinucleosides.

**Nucleoside coupling.** The initial attempts at coupling **7** and **9** with 5′-*O*-DMTr-thymidine were carried out using the Caruthers procedure with 4,5-dicyanoimidazole (DCI) as the activator. While formation of the product was observed, it was not a clean reaction. The *N*-methylimidazolium triflate (NMI·Tf) method reported for the monosubstitution of diamidites was found to be effective [53], resulting in relatively clean formation of amidites **10** and **11** (Scheme 3). The initial reaction produced a yellow foam, which was assumed to be the color of the desired products due to its similarity to **7** and **9**. However, the presence of unreacted 5′-*O*-DMTr-thymidine, the starting acyls **7** and **9**, and downfield ^31^P NMR peaks that are characteristic of diesters necessitated chromatographic separation. The isolation of **11** as a yellow solid as a 42.5:57.5 mixture of phosphorus epimers was carried out without difficulty. Little fractionation of diastereomers occurred during multiple chromatographies. In contrast, the isolation of **10** was somewhat tedious both due to the presence of impurities that were not easily separated and continuous fractionation of the phosphorus epimers that occurred during repeated chromatographies. However, **10** was ultimately obtained as a white foam.

Samples of the fast-moving diastereomer and of a mixture of the two diastereomers of **10** were fully characterized. They exhibited the characteristic carbonyl doublets at 226.9 and 226.8 ppm in the ^13^C NMR spectra (both with ^1^*J*_PC_ = 25.3 Hz). However, the infrared spectra could not be used to confirm the presence of the carbonyl since the thymidine ring exhibited a strong carbonyl absorption in the same region (i.e., 5′-*O*-DMTr-thymidine at 1684 cm^−1^, see Supporting Information File 1). The absence of room-temperature epimerization was apparent from both the chromatographic separation as well as the distinct peaks in the NMR spectra, and is well-known (as is facile chromatographic separation) for the analogous β-cyanoethoxyphosphoramidites [54–55]. A similar characterization of the mixture of diastereomers of **11** also exhibited two sets of peaks in the NMR spectra. However, one peculiarity involving only the diisopropylamino moiety was noted, namely, a fluctional process that resulted in broad peaks for the isopropylmethyl signals of both **10** and **11** in the ^1^H and ^13^C NMR spectra. Since this was not relevant to phosphorus epimerization, it was not further investigated.

Dinucleoside coupling was carried out using the 2:1 pyridinium trifluoroacetate/*N*-methylimidazole (PTFA/NMI) activator developed at Isis to combine phosphonamidites **10** and **11** with 3′-*tert*-butyldimethylsilyl-protected thymidine to give **12** and **13** [56]. The reactions were monitored by ^31^P NMR, giving fairly clean conversion from starting materials to the *P-*epimeric products. The reaction of **10** to give **12** was significantly faster than that of **11** to give **13**. That is, while the former was nearly complete in 30 min, the latter was not complete even in 2.5 h. However, prolonged reaction led to formation of diester impurities that could not be chromatographically separated. While not identified, the impurities could be due to detritylation although the characteristic orange color of the trityl cation was not seen. The diesters were less robust than the precursor monoester amidites. For instance, rapid chromatographic separation of the products from the ammonium salts formed during the synthesis was required to prevent further decomposition. The best procedure was found to involve short reaction times to minimize formation of the diester impurities, and the use of excess phosphonamidite since both **10** and **11** could be chromatographically removed from the dinucleosides. However, even a 50% excess was not sufficient to consume all of the 3′-*O*-(*tert*-butyldimethylsilyl)thymidine, and separation of this alcohol impurity from the acyl dinucleosides could not be achieved.

The characterization of the acyl dinucleosides was accomplished by 1D and 2D ^1^H and ^13^C NMR spectra, with confirmation of the dinucleoside structures by high-resolution positive ion ESI mass spectrometry. The characteristic carbonyl doublets shifted a few ppm upfield with larger P–C coupling constants than those observed for **10** and **11** (223.28 and 223.25 ppm, ^1^*J*_PC_ = 38.6 and 41.4 Hz for **12**, 211.6 and 211.1 ppm, ^1^*J*_PC_ = 44.3 and 42.4 Hz for **13**). Particularly, for **13**, the two deoxyribose hydrogen atom networks could be correlated with the two H1′ muliplets at 6.2–6.4 ppm in the 2D-COSY NMR spectrum. The identity of the two networks is based on the assumption that the downfield H1′ multiplets are due to the DMTr-thymidine, and the upfield H1′ multiplets are due to the *tert*-butyldimethylsilyl thymidine, as is seen for the parent alcohols [31]. For **12**, this analysis could not be carried out with as much certainty due to the larger amount of the 3′-*O*-*tert*-butyldimethylsilyl thymidine impurity. As previously observed for the β-cyanoethoxy dithymidyl triesters [31], the characteristic two pairs of thymidyl methyl doublets (due to allylic coupling to the H6 alkene hydrogen) on the two diastereomers were seen, one pair downfield near 1.8 ppm for the *tert*-butyldimethylsilyl-thymidine, and one pair upfield near 1.4 ppm for the DMTr-thymidine.

**Conversion to phosphorothioates.** An additional confirmation of the structures was possible via oxidation and conversion to the known diastereomeric phosphorothioates. As shown by Hata et al., acylphosphonate **3b** (Scheme 1) was converted to the corresponding phosphorothioate by treatment with a base (to give the *H*-phosphonate via loss of carboxylate) followed by elemental sulfur [43]. As shown in Scheme 3, oxidation of **12** and **13** with anhydrous *tert*-butyl hydroperoxide gave the acylphosphonates **14** and **15**. These products were immediately hydrolyzed by addition of approximately 2 equiv of aqueous triethylammonium bicarbonate (TEAB) to give the *H*-phosphonate **16**. Treatment of **16** with approximately 4 equiv of phenylacetyl disulfide (PADS) [57] gave the known dinucleoside phosphorothioate **17** [26,58].

**Phosphorus epimerization.** During the many attempts to purify the acyl dinucleosides by chromatography, it became apparent that some fractionation of the diastereomeric mixtures occurred. Due to the sensitivity of the acyls, chromatography on long columns of silica (with or without 1% NEt_3_ to deactivate the silica) gave rise to the loss of acyls, without any improvement in diastereomer separation. However, the individual samples of fractionated diastereomers of **12** or **13** showed no evidence of room-temperature epimerization. Heating of samples at temperatures from 50 to 150 °C over several hours was carried out in acetonitrile, as was done for the epimeric phosphite triesters [31]. Although this was not the most rigorous test (since available samples only ranged in diastereomer ratios from 1:1 to 1:1.4), no detectable change in the diastereomer ratios was observed. More importantly, sample decomposition occurred at all temperatures. Because of this, it would have been impossible to distinguish between small changes in diastereomer ratio and small differences in rates of diastereomer decomposition.

**Scheme 4:**
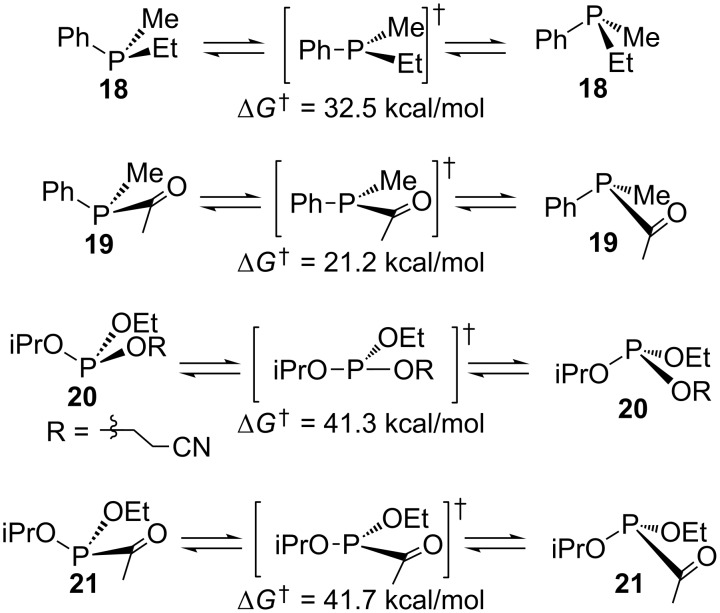
Calculated phosphine, acylphosphine, phosphite, and acylphosphonite inversion barriers.

**Calculation of acylphosphonite inversion barrier.** As described in the Introduction, inversion barriers for phosphines, acylphosphines, and phosphites are known. These were calculated first for validation of the method. The structures of chiral phosphine **18**, chiral acylphosphine **19**, and chiral phosphite **20** (Scheme 4) were each minimized by DFT using Gaussian (see Supporting Information File 1 for details), and the vibrational calculation confirmed a local minimum for each structure. While the phosphites are complicated by multiple local minima, which depend on the conformations about the P–O bonds, the energies are comparable (separated by less than 1 kcal/mol) and do not have a significant impact on the inversion barriers. Transition states were located using routines implemented in Gaussian (see Supporting Information File 1 for details), and in all cases the vibrational calculation exhibited one negative frequency that corresponded to the phosphorus inversion vibration. Not surprisingly, the phosphine transition state exhibited a trigonal planar geometry, and the gas-phase energy barrier of 32.5 kcal/mol was in excellent agreement with the values reported by Mislow for reactions carried out in non-polar toluene as a solvent. Similarly, the acylphosphine transition state exhibited a trigonal planar geometry, and the gas-phase energy barrier of 21.2 kcal/mol was also in excellent agreement with the typical values noted above. The phosphite, in contrast, did not yield a trigonal planar transition state, but rather gave a T-shaped transition state. In fact, such a result has been reported for PF_3_ [59–60] and is due to the presence of the highly electronegative atoms bound to phosphorus. While the alkoxy groups of the phosphite triester are considered to be π-electron donors, they are strongly electronegative. This results in an inversion geometry that is rather different from the phosphines, presumably mediated by oxygen–phosphorus lone pair–lone pair repulsion. The calculated barrier of 41.3 kcal/mol (from the slightly higher ground state geometry) was not in good agreement with that reported, being 8.3 kcal/mol higher. Unlike the other calculations, this one incorporated the polar acetonitrile solvent that was used for the epimerization kinetics [31], but it had essentially no effect on the calculated barrier.

The transition state calculations were then carried out on the chiral acylphosphonite **21** with the expectation that a high phosphite-like barrier might occur. In fact, the barrier of 41.7 kcal/mol was slightly higher than the phosphite triester calculation. Unlike the phosphite T-shaped transition state, this occurs via a trigonal planar transition state, although like the others with one negative frequency corresponding to phosphorus inversion. The high barrier is consistent with the absence of acylphosphonite epimerization of **12** and **13**, and this suggests that any amide-like stabilization of the phosphorus lone pair in a trigonal transition state is eliminated by oxygen–phosphorus lone pair–lone pair repulsion.

## Conclusion

High-yield syntheses have been developed for some novel phosphorus synthons, including the acyl and benzoyldiamidites via the surprising condensation of phosphonodiamidite **4** and the two acid chlorides with elimination of HCl. The nucleoside acylphosphonamidites were also readily prepared, although the benzoyl analog **11** was more easily isolated (albeit as the diastereomeric mixture of *P*-epimers), while the acetyl analog **10** allowed the diastereomers to be separated following repeated chromatographies. Final coupling to give the dinucleoside acylphosphonites proceeded smoothly, but separation of the pure compounds from the nucleoside precursors could not be completely achieved. If the pure acylphosphonites are to be obtained, solid-phase synthesis would probably be required. The chromatography results suggested that room-temperature epimerization at phosphorus did not occur, and heating at 50–150 °C also gave no indication of epimerization. Density functional theory calculations are consistent with this result, giving a calculated inversion barrier of 42 kcal/mol. While one might argue that given the results of such a calculation, the lack of epimerization was a foregone conclusion, it is still important to test theory with experiment. In conclusion, the analogy between phosphines and phosphites, in which the low barrier to acylphosphine inversion suggested that the same might occur with acylphosphonites, is in this case superficial, and the anticipated low-temperature inversion does not occur.

## Supporting Information

Supporting Information contains the experimental procedures, characterization of new compounds, copies of ^1^H, ^13^C and ^31^P NMR spectra, IR spectra, details of the X-ray structure determination, and details of the DFT calculations.

File 1 Experimental.

File 2 CIF file of compound **9**.
