# Supplementary material for: Synthesis of dinucleoside acylphosphonites by phosphonodiamidite chemistry and investigation of phosphorus epimerization
Source: Beilstein J Org Chem. 2015 Jan 30;11:184–91. doi: 10.3762/bjoc.11.19 (PMC4362088; doi:10.3762/bjoc.11.19)
Supplement: File 1 — Experimental. [file Beilstein_J_Org_Chem-11-184-s001.pdf]

Supporting Information

for

Synthesis of dinucleoside acylphosphonites by phosphonodiamidite chemistry and investigation of phosphorus epimerization

William H. Hersh*

Address: Department of Chemistry and Biochemistry, Queens College and the Graduate Center of the City University of New York, Queens, NY 11367-1597, USA

Email: William H. Hersh - william.hersh@qc.cuny.edu

*Corresponding author

Experimental

Table of Contents

**General Methods**	S2
**$(iPr_2N)_2PC(O)CH_3$ (7).**	S2
**$(iPr_2N)_2PC(=CH_2)OC(O)CH_3$ (8).**	S3
**$(iPr_2N)_2PC(O)C_6H_5$ (9).**	S4
**3′-(5′-DMTr-OT)P(N(iPr)$_2$)C(O)CH$_3$ (10).**	S5
**3′-(5′-DMTr-OT)P(N(iPr)$_2$)C(O)C$_6$H$_5$ (11).**	S8
**3′-(5′-DMTr-OT)-5′-(3′-*t*-BuMe$_2$Si-OT)PC(O)CH$_3$ (12).**	S9
**3′-(5′-DMTr-OT)-5′-(3′-*t*-BuMe$_2$Si-OT)PC(O)C$_6$H$_5$ (13).**	S11
**Conversion of 12 and 13 to 17.**	S12
**Thermal decomposition of 12 and 13.**	S13
**X-ray structure of 9.**	S14
**Calculations.**	S15
**References.**	S16
**^{1}H, ^{13}C, ^{31}P, COSY90, HETCOR, HSQC, and IR spectra.**	S17
**XYZ coordinates from DFT optimization of 7.**	S58
**XYZ coordinates from DFT optimization of 8.**	S59
**XYZ coordinates from DFT optimization of 18.**	S60
**XYZ coordinates from DFT optimization of 19.**	S62
**XYZ coordinates from DFT optimization of 20.**	S63
**XYZ coordinates from DFT optimization of 21.**	S65

Experimental

General methods. All reactions and sample analysis preparations were carried out in an inert-atmosphere glovebox with recirculating nitrogen, using oven-dried glassware. NMR spectra were recorded on 400 and 500 MHz Bruker spectrometers referenced to $CDCl_3$ solvent peaks [1,2] and for ^{31}P NMR to external PPh_3 at -5.25 ppm. Peak assignments were made where possible using 2D COSY and HETCOR or HSQC spectra, with ^{13}C-^{1}H correlations shown in the spectral data where needed, as well as by comparison to the thymidine starting materials. Reaction solvents were distilled under nitrogen and then dried over activated 3 Å molecular sieves [3]. Column chromatography was carried out in the glovebox on 230-400 mesh silica gel that had been dried several hours at 250 °C under vacuum. For the peak assignments in the ^{1}H NMR spectra of **12** and **13**, the 3′-phosphorylated thymidine and the 5′-phosphorylated thymidine are labeled T1 and T2, respectively.

$(iPr_2N)_2PC(O)CH_3$ (7). The starting material $(iPr_2N)_2PH$ (**4**) was prepared via a modification of the literature procedure [4]. In the glovebox, powdered $LiAlH_4$ (0.242 g, 6.37 mmol, 1.17 equiv) was added in one portion to 1.451 g (5.44 mmol) of $(iPr_2N)_2PCl$ [5] in 10 mL of THF, and the suspension was stirred vigorously for two hours; while it was stoppered, the stopper was removed periodically to release pressure. The grey suspension was filtered through a layer of dry Celite (*CAUTION: the grey solid smokes and occasionally briefly ignites when removed from the glovebox)*, and the solvent was then removed from the yellow solution using a vacuum pump. The resultant white solid suspended in a yellow oil was extracted with 7 × ~8 mL of hexanes, filtering each 8 mL extract through dried Celite. Solvent removal using a vacuum pump gave 1.16 g (5.00

mmol, 92% yield) of **4** as a white solid suspended in a clear oil, which on the basis of ^{1}H and ^{31}P NMR was ~93% pure. This material could be stored cold in the glovebox at –35 °C but was typically used immediately.

A sample of **4** (0.311 g, 1.34 mmol) was suspended in 7 mL CH_2Cl_2, the flask was fitted with a dropping funnel containing a solution of 0.119 g of $CH_3C(O)Cl$ (1.52 mmol, 1.13 equiv) in 3 mL of CH_2Cl_2, and the dropping funnel was then attached to a solenoid-controlled vacuum valve. The acetyl chloride solution was added dropwise over ~1 min, while periodically opening the reaction to vacuum in order to keep the reaction under partial vacuum. Gentle bubbling of the solution occurred, presumably due to release of HCl gas, giving a yellow solution. Solvent removal gave a yellow solid that was then extracted with 3 × 5 mL of hexanes (the last extract was clear), filtering each extract through Celite. Final solvent removal gave 0.234 g (0.852 mmol, 64% yield) of **7** as a yellow oil at room temperature; storage at –35 °C gave a crystalline mass but it quickly melted at room temperature. The material so produced was used as is with no further purification; on the basis of ^{31}P NMR it was > 95% pure. ^{1}H NMR (400 MHz, $CDCl_3$) δ 3.29 (m, $(CH_3)_2CH$, 4H), 2.27 (d, $^{3}J_{PH}$ = 8.8 Hz, $CH_3C(O)$ 3H), 1.23 (d, J = 6.8 Hz, 12H), 1.18 (d, J = 6.4 Hz, 12H); ^{13}C NMR (100 MHz, $CDCl_3$) δ 227.9 (d, $^{1}J_{PC}$ = 22.4 Hz), 49.8 (br d, $(CH_3)_2CH$, $^{2}J_{PC}$ = 9.1 Hz), 30.7 (d, $CH_3C(O)$, $^{2}J_{PC}$ = 49.7 Hz), 24.4 (d, J = 6.2 Hz), 24.3 (d, J = 6.4 Hz); ^{31}P NMR (162 MHz, $CDCl_3$) δ 63.5; IR ($CDCl_3$) 2969, 1654 cm^{-1}. HRMS (ESI): Calcd for $C_{14}H_{32}N_2OP$ $[M+H]^+$ 275.2247, found 275.2247.

$(iPr_2N)_2PC(=CH_2)OC(O)CH_3$ (8). Reaction of **4** (0.876 g, 3.77 mmol), $CH_3C(O)Cl$ (0.319 g, 4.06 mmol, 1.08 equiv), and Et_3N (0.392 g, 3.87 mmol, 1.03 equiv) in 13 mL of CH_2Cl_2 gave a mixture of 43% **8**, 32% **7**, and 25% unreacted **4**, on the basis of

integration of the ^{1}H and ^{31}P NMR spectra. ^{1}H NMR (400 MHz, $CDCl_3$) δ 5.48 (t, J_{HH} = J_{PH} = 1.1 Hz, 1H), 5.29 (dd, J_{HH} = 1.1 Hz, J_{PH} = 7.3 Hz, 1H), 2.09 (s, 3H); ^{13}C NMR (100 MHz, $CDCl_3$) δ 168.8 (s), 161.5 (d, J_{PC} = 8.8 Hz), 111.4 (d, J_{PC} = 16.8 Hz), 48.0 (d, J_{PC} = 12.0 Hz, $CH(CH_3)_2$); ^{31}P NMR (162 MHz, $CDCl_3$) δ 49.7. The iPr peaks could not be identified except as noted, and it appeared that the presumed $OC(O)CH_3$ carbon also overlapped the iPr region on the basis of the DEPT NMR.

$(iPr_2N)_2PC(O)C_6H_5$ (9). A sample of **4** (0.852 g, 3.67 mmol) prepared as described for the synthesis of **7** was suspended in 10 mL CH_2Cl_2 and Et_3N (0.402 g, 3.97 mmol, 1.08 equiv). The flask was fitted with a dropping funnel containing a solution of 0.563 g of $C_6H_5C(O)Cl$ (4.01 mmol, 1.09 equiv) in 4 mL of CH_2Cl_2, which was then attached to a solenoid-controlled vacuum valve. The benzoyl chloride solution was added dropwise over ~1 min, while periodically opening the reaction to vacuum in order to keep the reaction under partial vacuum. Gentle bubbling of the solution occurred, presumably due to release of some HCl gas, giving an orange solution. Solvent removal gave an orange solid that was then extracted with 4 × 5 mL of hexanes (the last extract was clear), filtering each extract through Celite. Final solvent removal gave 1.10 g (3.27 mmol, 89% yield) of **9** as a yellow-orange solid. The material so produced was used as is with no further purification; on the basis of ^{31}P NMR it was > 99.6% pure. ^{1}H NMR (400 MHz, $CDCl_3$) δ 8.00 (m, H_{ortho}, 2H), 7.46 (m, H_{para}, 1H), 7.38 (t, J = 7.8 Hz, H_{meta}, 2H), 3.32 (m, 4H), 1.21 (d, J = 6.8 Hz, 12H), 1.08 (d, J = 6.8 Hz, 12H); ^{13}C NMR (100 MHz, $CDCl_3$) δ 220.9 (d, $^{1}J_{PC}$ = 23.7 Hz), 140.8 (d, $^{2}J_{PC}$ = 39.7 Hz, C_{ipso}), 132.3 (d, $^{5}J_{PC}$ = 1.5 Hz, C_{para}), 128.2 (C_{meta}), 127.9 (d, $^{3}J_{PC}$ = 11.8 Hz, C_{ortho}), 49.5 (br d, $^{2}J_{PC}$ = 8.1 Hz), 24.3 (d, $^{3}J_{PC}$ = 5.7 Hz), 23.9 (d, $^{3}J_{PC}$ = 6.1 Hz); ^{31}P NMR (162 MHz, $CDCl_3$) δ

59.3; IR ($CDCl_3$) 2969, 1631 cm^{-1}HRMS (ESI): Calcd for $C_{19}H_{34}N_2OP$ $[M+H]^+$ 337.2403, found 337.2402.

3′-(5′-DMTr-OT)P(N(iPr)$_2$)C(O)CH$_3$ (10). Solid *N*-methylimidazolium triflate (NMI·Tf, 0.399 g, 1.72 mmol, 0.74 equiv) [6] was added to a suspension of **7** (0.639 g, 2.33 mmol,) and 5′-*O*-(4,4′-dimethoxytrityl)thymidine (5′-DMTr-OT) [7] (1.20 g, 2.21 mmol, 0.95 equiv) in 10 mL acetonitrile. After stirring for 1.25 h, only a small amount of solid remained and the solution was filtered through Celite. Solvent removal using a vacuum pump gave a foamy yellow solid that was taken up in 10 mL benzene, 20 mL ether was added to precipitate salts, the mixture was filtered, and the solvent was again removed using a vacuum pump to give a yellow solid. This was stirred with 10 mL hexane to remove some of the starting acyl, giving 1.56 g of product as a yellow powder (99% crude yield) that was 12% starting acyl and 85% product by ^{31}P NMR but also contained impurities of DMTr-OT and $iPr_2NH_2^+$ Tf^-. Significant purification was achieved by taking up 1.32 g of this material in 8 mL benzene, filtering, and then precipitating out the product by addition of 24 mL of hexane. After cooling for 1 hr at –35 °C, the solvent was poured off and the residue pumped under vacuum to give a sticky orange solid; final solvent removal was achieved by addition of a small amount of ether and pulling a vacuum again to give a yellow foam (1.26 g, 95% recovery) that was 89% pure by ^{31}P NMR.

Chromatography of 0.65 g of this material on 40 mL of silica gel on a 60 mL fritted funnel, eluting with 9:1 CH_2Cl_2:THF, gave a yellow band collected in three fractions (60 mL 9:1 CH_2Cl_2:THF, 20 mL 1:1 CH_2Cl_2:THF and 40 mL THF); all three fractions exhibited a spot with R_f = 0.5-0.55 on TLC (9:1 CH_2Cl_2:THF), with material at

the origin eluting at the end of the last fraction. Analysis by ^{31}P NMR showed that the first two fractions (97.8 mg) were ~95% pure and ~86% the "fast" isomer at 117.6 ppm, while the third fraction (429 mg; 81% total recovery) was ~81% pure and ~33:67 "fast": "slow" isomers at 117.6 and 116.8 ppm. The two samples were separately re-chromatographed.

The "fast" isomer was chromatographed on 10 mL of silica eluting only with 9:1 CH_2Cl_2:THF, and gave in the first two UV-active fractions 57.1 mg of material that was ~95% pure as a 93:7 mixture of "fast":"slow" isomers. ^{1}H NMR (500 MHz, $CDCl_3$) δ 8.51 (br s, NH, 1H), 7.66 (~q, $^{4}J \approx 1.2$ Hz, H_6, 1H), 7.40 (m, Ph, 2H), 7.31 – 7.22 (m, Ph and 4H of $MeOC_6H_4$ AA′BB′, 3H), 6.83 (m, 4H of $MeOC_6H_4$ AA′BB′), 6.40 (dd, ^{3}J = 7.7, 5.9 Hz, H_1', 1H), 4.65 (ddd, $^{3}J \approx 3.2$ Hz, H_3', 1H), 4.32 (~ddd, $^{3}J \approx 1.3$ Hz, $H_{4'}$, 1H), 3.79 (s, *Me*OC_6H_4, 6H), 3.52 (dd, ^{2}J = 10.7 Hz, ^{3}J = 2.8 Hz, H_5', 1H), 3.37 (dd, ^{2}J = 10.7 Hz, ^{3}J = 2.6 Hz, H_5', 1H), 3.28 (m, $CH(CH_3)_2$, 2H), 2.51 (m, H_2', 1H), 2.33 (m, H_2', 1H), 2.24 (d, $^{3}J_{PH}$ = 5.4 Hz, $CH_3C(O)P$, 3H), 1.44 (d, ^{4}J = 1.0 Hz, CH_3C_1, 3H), 1.19 (br d, ^{3}J = 7.8 Hz, $CH(CH_3)_2$, 3H), 1.18 (br d, ^{3}J = 7.3 Hz, $CH(CH_3)_2$, 3H); ^{13}C NMR (125.8 MHz, $CDCl_3$) δ 226.9 (d, $^{1}J_{PC}$ = 25.3 Hz), 163.8, 158.9, 150.3, 144.4, 135.7 (CH_1), 135.5 (4°), 135.4 (4°), 130.27, 130.26 (Ar CH ~7.3; $MeOC_6H_4$ CH ~6.8), 128.3 (Ph CH ~7.4), 128.1, (Ar CH, ~7.3), 127.3 (Ar CH, ~7.25), 113.40, 113.38 (Ar CH, ~7.3; $MeOC_6H_4$ CH, ~6.8), 111.3 (4°), 87.1 (4°), 85.7 (d, $^{3}J_{PC4'}$ = 5.4 Hz, C_4'), 84.9 (CH_1', 6.40), 77.6 (d, $^{2}J_{PC3'}$ = 19.9 Hz, CH_3'), 63.3 (CH_5'), 55.4 (*Me*O, 3.79), 46 (iPr *C*H, from HSQC cross peak with ^{1}H at δ 3.28), 40.5 (d, $^{3}J_{PC2'}$ = 3.9 Hz, CH_2'), 30.8 (d, $^{2}J_{PC}$ = 36.4 Hz, $CH_3C(O)P$), ~25 (broad, iPr Me at 1.19 and 1.18), 15.4 (iPr Me at 1.19 and 1.18), 11.9 (CH_3C_1); ^{31}P NMR (162

MHz, $CDCl_3$) δ 117.6, 116.8 (93.2:6.8); IR ($CDCl_3$) 3396, 2969, 1688 cm^{-1}. HRMS (ESI): Calcd for $C_{39}H_{49}N_3O_8P$ $[M+H]^+$ 718.3252, found 718.3252.

The 32:68 “fast”:“slow” mixture was chromatographed on 30 mL of silica eluting only with 9:1 CH_2Cl_2:THF, giving three fractions as white foams consisting of isomeric mixtures of the “fast”:“slow” isomers as follows: 46:54 (~96% pure, 81.5 mg), 25:75 (~91% pure, 40.0 mg), and 18:82 (~92% pure, 55.7 mg). Detailed spectra were obtained for the 46:54 mixture (only peaks for the “slow” isomer are given except as noted): ^{1}H NMR (500 MHz, $CDCl_3$) δ 8.82, 8.77 (fast, slow, br s, NH), 7.60 (~q, $^4J \approx 1.3$ Hz, H_1), 7.39 (m, 2H, Ph), 7.30 – 7.26 (m, 3H, Ph and 4H of $MeOC_6H_4$ AA′BB′), 6.83 (m, 4H of $MeOC_6H_4$ AA′BB′), 6.48 (dd, $^3J = 8.2, 5.7$ Hz, $H_{1'}$), 4.64 (m, $H_{3'}$), 4.19 (~q, $^3J \approx 2.6$ Hz, $H_{4'}$), 3.78 (s, 6H, *Me*OC_6H_4), 3.47 (dd, $^2J = 10.6$ Hz, $^3J = 2.7$ Hz, $H_{5'}$), 3.32 (dd, $^2J = 10.6$ Hz, $^3J = 2.7$ Hz, $H_{5'}$), 3.23 (m, $CH(CH_3)_2$, 2H), 2.65 (m, $H_{2'}$, 1H), 2.33 (m, $H_{2'}$, 1H), 2.34 (d, $^3J_{PH} = 5.4$ Hz, $CH_3C(O)P$), 1.44 (br s (overlaps fast isomer), CH_3C_1), 1.40 (d, $^3J = 6.7$ Hz, part of $CH(CH_3)_2$), 1.17 (br m, $CH(CH_3)_2$), 1.04 (br d, $^3J = 5.3$ Hz, part of $CH(CH_3)_2$); ^{13}C NMR (125.8 MHz, $CDCl_3$) δ 226.8 (d, $^1J_{PC} = 25.3$ Hz), 163.9, 158.9, 150.4, 144.3, 135.7 (CH_1), 135.5 (4°), 135.4 (4°), 130.25, 130.21 (Ar CH ~7.3; $MeOC_6H_4$ CH ~6.8), 128.3 (Ph CH ~7.4), 128.1, (Ar CH, ~7.3), 127.3 (Ar CH, ~7.25), 113.39, 113.37 (Ar CH, ~7.3; $MeOC_6H_4$ CH, ~6.8), 111.4 (4°), 87.1 (4°), 85.8 (d, $^3J_{PC4'} = 4.7$ Hz), 84.9 ($CH_{1'}$, 6.48), 78.0 (d, $^2J_{PC3'} = 19.0$ Hz), 63.5 ($C_{5'}$), 55.3 (*Me*O, 3.79), 46.7 (iPr *C*H, from HSQC cross peak with ^{1}H at δ 3.3), 40.1 (d, $^3J_{PC2'} = 4.7$ Hz), 30.83 (d, $^2J_{PC} = 36.8$ Hz, $CH_3C(O)P$), ~25 (broad, iPr Me at 1.17, 1.04), 19.3 (iPr Me at 1.40), 11.9 (CH_3C_1); ^{31}P NMR (162 MHz, $CDCl_3$) δ 117.6, 116.8 (46:54); IR ($CDCl_3$) 3396,

2969, 1688 cm^{-1}. HRMS (ESI): Calcd for $C_{39}H_{49}N_3O_8P$ $[M+H]^+$ 718.3252, found 718.3254.

3′-(5′-DMTr-OT)P(N(iPr)$_2$)C(O)C$_6$H$_5$ (11). An orange suspension of **9** (0.502 g, 1.49 mmol, 1.07 equiv), DMTr-OT (0.758 g, 1.39 mmol), and NMI·Tf (0.326 g, 1.40 mmol, 1.01 equiv) in 10 mL acetonitrile was stirred for 2 h to give a clear orange solution. Solvent removal using a vacuum pump gave an orange-yellow foam. Chromatography on 30 mL of silica gel on a 60 mL fritted funnel, eluting with 9:1 CH_2Cl_2:THF gave a yellow band in 60 mL of solvent, discarding a pale yellow tail; solvent removal gave 0.757 g (70% crude yield) of yellow foam consisting of product and starting material. Final purification was achieved by taking up the material in 4 mL ether, and precipitating out product by addition of 10 mL of hexane with swirling, cooling briefly to –35 °C, and filtration to give the product as a yellow solid. Addition of CH_2Cl_2 followed by solvent removal was required to remove the hexane, giving 0.632 g of yellow foam (58% yield). ^{1}H NMR (500 MHz, $CDCl_3$) δ 8.70, 8.62 (minor, major, br s, NH, 1H), 7.91 (m, 2H of Ph), 7.69, 7.62 (minor, major, ~q, $^4J \approx 1.6$, 1.3 Hz, H_1, 1H), 7.62 – 7.2 (m, 8H of Ph and 4H of $MeOC_6H_4$ AA′BB′), 6.80 (m, 4H of $MeOC_6H_4$ AA′BB′), 6.51, 6.40 (major, minor, dd, ^3J = 7.8, 6.2, and 7.2, 6.2 Hz, $H_1′$, 1H), 4.70 (m, $H_3′$, 1H), 4.43, 4.26 (minor, major, ~q, $^3J \approx 3.0$, 2.7 Hz, $H_4′$, 1H), 3.781, 3.779 and 3.73, 3.71 (major, minor, each two diastereotopic s, *Me*OC_6H_4, 6H), 3.52, 3.43 (m, $(H_5′)_2$, 2H), 3.27 (m, C*H*$(CH_3)_2$, 2H), 2.73 (major, ddd, ^2J = 13.8 Hz, ^3J = 6.1, 2.7 Hz, $H_2′$, ~0.5 H), 2.58 (minor, ddd, ^2J = 13.6 Hz, ^3J = 5.9, 3.2 Hz, $H_2′$, ~0.5 H), 2.34 (m, $H_2′$, 1H), 1.45 (br s, C*H$_3$*C$_1$, 3H), 1.22, 1.09, 0.7 (br, CH(C*H$_3$*)$_2$, 6H); ^{13}C NMR (125.8 MHz, $CDCl_3$) δ 217.2, 217.1 (major, minor, d, $^1J_{PC}$ = 30.6, 29.9 Hz), 163.94, 163.90, 163.86, 163.84, 163.82,

158.83, 158.81, 158.77, 158.73, 150.34, 150.30, 144.58, 144.46, 139.08, 139.02, 138.81, 138.75 (all 4°), 135.92 (CH_1 (major) 7.62), 135.82 (CH_1 (minor) 7.69), 135.55, 135.52, 135.51 (4°), 133.16, 133.08 (Ar CH ~7.5), 130.25, 130.22 ($MeOC_6H_4$ CH ~7.3), 128.5, 128.4, 128.3, 128.09, 128.06, 127.89, 127.83, 127.24, 127.17 (Ar CH 7.9-7.2), 113.36, 113.34, 113.32 ($MeOC_6H_4$ CH ~6.8), 111.22, 111.14 (4°), 86.99, 86.98 (4°), 85.8, 85.6 (d, $^3J_{PC4'}$ = 5.5, 4.3 Hz), 85.0, 84.8 ($CH_{1'}$, major, minor), 77.32, 77.16, 76.96 (visible in DEPT45, $C_{3'}H$ 4.70), 63.5, 63.1, 55.4, 55.30, 55.28 (*Me*O, 3.78-3.71), 46.6, 40.2, ~24.5 (broad, iPr Me), 11.9 (CH_3C_1); ^{31}P NMR (162 MHz, $CDCl_3$) δ 119.8, 116.7 (42.5:57.5); IR ($CDCl_3$) 3396, 2970, 1688 cm^{-1}. HRMS (ESI): Calcd for $C_{44}H_{51}N_3O_8P$ $[M+H]^+$ 780.34083, found 780.33935.

3′-(5′-DMTr-OT)-5′-(3′-*t*-BuMe$_2$Si-OT)PC(O)CH$_3$ (12). To a stirred solution of 69.4 mg of **10** (0.097 mmol, 1.46 equiv) and 23.6 mg of 3′-*O*-(*tert*-butyldimethylsilyl)thymidine [8,9] (3′-TBS-OT, 0.066 mmol, 1 equiv) in 1 mL of acetonitrile was added 0.95 g (1.2 mL) of 0.20 M/0.10 M pyridinium trifluoroacetate/*N*-methylimidazole (PTFA/NMI) in acetonitrile (0.24/0.12 mmol, 2.5/1.2 equiv relative to **10**), in one portion. The clear solution was stirred for 25 min, the solution was concentrated to about half its volume using a vacuum pump, and the solution was applied to a column of 6 mL of silica gel packed in THF. A UV-active band was eluted in about 10 mL of THF, the solvent was removed using a vacuum pump, and triturated with ether to give 105.4 mg of pale yellow foam. ^{31}P NMR indicated complete reaction of **10** and the presence of ~12% of unidentified material in the dinucleoside acyl region, and ^1H NMR indicated ~14% of unreacted 3′-TBS-OT. The mixture was applied in 2 mL of CH_2Cl_2 to a 15 mL column of silica packed in 5% THF in CH_2Cl_2. After elution with 20

mL of 5% THF in CH_2Cl_2 followed by 10 mL of 10% THF in CH_2Cl_2, unidentified weakly UV-active material (6.6 mg total) eluted in 5 mL of 10% THF in CH_2Cl_2 followed by 20 mL of 20% THF in CH_2Cl_2. The remaining UV-active material eluted in 30 mL of 20-30% THF in CH_2Cl_2 followed by 20 mL of THF, giving 54.5 mg of **12** (~85% yield) that was ~84% pure by ^{31}P NMR and contained ~14 mol% unreacted 3′-TBS-OT. Rechromatography of this material combined with similar fractions from prior syntheses (99 mg total) on a 15 mL column of silica packed in 10% THF in ethyl acetate gave after a 17 mL forerun an 8 mL UV-active fraction with virtually all the **12** recovered (55 mg), containing 6% of an unidentified impurity (^{31}P NMR: 142.9 ppm) and ~36 mol% of 3′-TBS-OT. ^{1}H NMR (500 MHz, $CDCl_3$) δ 9.57-9.35 (5 s, NH, 2H), 7.59, 7.55 (q, $^4J \approx 1.2$, 1.1 Hz, H_6, 1H), 7.37 (m, 3H), 7.26 (m, 7H), 6.83 (d, J = 7.5 Hz, 4H of $MeOC_6H_4$ AA′BB′), 6.42, 6.39 (dd, ^3J = 8.5, 6.0 Hz and 7.5, 6.0 Hz, $^{T1}H_1′$, 1H), 6.29, 6.24 (t, ^3J = 6.5, 6.8 Hz, $^{T2}H_1′$, 1H), 4.80 (~q, $^3J \approx 6.8$ Hz, $^{T1}H_3′$, 0.5 H), 4.38,4.21, 4.06 (m, ~2H), 3.93, 3.89 (m, 2H, overlapping with 3′-TBS-OT), 3.781, 3.778 (s, MeO, 6H), 3.52, 3.36 (m, $^{T1}H_5′$, 1.5H), 2.91 (m,$^{T1}H_5′$, 0.5 H), 2.6-2.2 (m, $H_2′$, 4H), 2.36, 2.28 (d, J = 4.0 and 4.0 Hz, CH_3C(O), 3H, with overlapping 3′-TBS-OT), 1.87, 1.83 (s, $^{T2}CH_3$C-5, 3H), 1.46, 1.45 (d, $^4J \approx 0.5$ Hz, $^{T1}CH_3$C-5, 3H), 0.88 (s, *t*-Bu, 9H), 0.078, 0.054, 0.040 (s, Me_2Si, 6H); ^{13}C NMR (125.8 MHz, $CDCl_3$) δ 223.28, 223.25 (d, $^1J_{PC}$ = 38.6, 41.4 Hz), 164.11, 164.04, 158.9, 150.68, 150.63, 150.51, 144.20, 144.16 (all 4°), 137.15, 136.0, 135.24 (*C*H_6 7.59, 7.55), 135.17 (*C*H_6 7.59, 7.55), 130.2, 128.2, 127.4, 113.4, ($MeOC_6H_4$ 6.83), 111. 7 (4°), 111.27 (4°), 111.20 (4°), 111.11 (4°), 87.7 (3′-TBS-OT, *C*$H_1′$), 87.33, 87.28, 87.0, 86.0, 85.7, 85.4, 85.1, 84.75, 84.69, 71.7, 71.36, 71.32, 68.4, 66.7, 63.2, 63.1, 62.1, 55.4 (MeO), 40.75, 40.59, 39.9, 30.2 (*C*H_3C(O)), 29.9 (*C*H_3C(O)), 25.83 (*t*-Bu),

25.79 (t-Bu), 18.06, 18.04, 18.00 (t-Bu 4° C), 12.63, 12.54 ($^{T2}CH_3$C-5), 11.99, 11.92 ($^{T1}CH_3$C-5), -4.52, -4.58, -4.72, -4.74 (CH_3Si); ^{31}P NMR (202 MHz, $CDCl_3$) δ 148.2, 145.2 (49:51); IR ($CDCl_3$) 3395, 2957, 2932, 1690 cm^{-1}. HRMS (ESI): Calcd for $C_{49}H_{62}N_4O_{13}PSi^+$ [M+H]$^+$ 973.38148, found 973.38100.

3′-(5′-DMTr-OT)-5′-(3′-*t*-BuMe$_2$Si-OT)PC(O)C$_6$H$_5$ (13). To a stirred solution of 118.0 mg of **11** (0.151 mmol, 1.34 equiv) and 40.0 mg of 3′-TBS-OT (0.112 mmol, 1 equiv) in 2 mL of acetonitrile was added 1.51 g (1.93 mL) of 0.20 M/0.10 M PTFA/NMI in acetonitrile (0.385/0.193 mmol, 2.6/1.3 equiv relative to **11**), in one portion. The yellow solution was stirred for 30 min, the solution was concentrated to about half its volume using a vacuum pump, and the solution was applied to a column of 7 mL of silica gel packed in THF. The yellow band was eluted in about 10 mL of THF, the solvent was removed using a vacuum pump, and triturated with ether to give 156.8 mg of yellow foam. ^{31}P NMR indicated the mixture contained 34 mol% of **11**. The mixture was applied in 3 mL of CH_2Cl_2 to a 15 mL column of silica packed in 5% THF in CH_2Cl_2. After elution with 32 mL of 5% THF in CH_2Cl_2 followed by 10 mL of 10% THF in CH_2Cl_2, unreacted **11** eluted in 45 mL of 10% THF in CH_2Cl_2, towards the end of which ~6 mg of **13** was eluted. The main yellow band eluted in 30 mL of 1:1 THF:CH_2Cl_2, giving 88 mg of **13** as a yellow solid, containing 7.5% of unidentified impurities (^{31}P NMR: 150.1, 148.0 ppm, 5.5% and 2% respectively), a trace of **11**, and ~12% of 3′-TBS-OT. ^{1}H NMR (500 MHz, $CDCl_3$) δ 9.19-9.05 (4 s, NH, 2H), 7.96-7.93 (m, Ar, 2H), 7.60-7.19 (m, Ar, $^{T1}H_6$, $^{T2}H_6$, 14H), 6.84-6.81 (m, 4H of MeOC_6H_4 AA′BB′), 6.44, 6.40 (dd, ^3J = 7.8, 6.3 Hz and 8.5, 5.5 Hz, $^{T1}H_1$′, 1H), 6.30 (m, $^{T2}H_1$′, 1H), 4.86, 4.79 (~br dd, $^3J \approx$ 6.9, 6.9 Hz, $^{T1}H_3$′, 1 H), 4.43, 4.34 (~dd and m, $^3J \approx$ 9.0, 5.0 Hz, $^{T2}H_3$′, 1 H), 4.2-4.00 (m, 1H, $^{T1}H_4$′, 2H,

$^{T2}H_{5}'$), 3.93 (m, 1H overlapping with 3′-TBS-OT, $^{T2}H_{4}'$), 3.786, 3.785, 3.77 (s, MeO, 6H), 3.44-3.34 (m, $^{T1}H_{5}'$, 1.5 H), 3.11 (dd, ^{3}J = 2.3 Hz, ^{2}J = 10.8 Hz,$^{T1}H_{5}'$, 0.5 H), 2.50, 2.42 (br dd, ^{3}J = 12.3, 5.3 Hz and 13.1, 5.8 Hz, $^{T1}H_{2}'$, 1H), 2.38-2.23 (m, $^{T1}H_{2}'$, 1H), 2.22-2.11 (m, $^{T2}H_{2}'$, 2H), 1.85, 1.79 (d, $^{4}J \approx$ 0.7 Hz, $^{T2}CH_{3}$C-5, 3H), 1.45, 1.38 (d, $^{4}J \approx$ 0.7 Hz, $^{T1}CH_{3}$C-5, 3H), 0.86 (s, *t*-Bu, 9H), 0.032, 0.028, 0.014, 0.003 (s, $Me_{2}Si$, 6H); ^{13}C NMR (125.8 MHz, $CDCl_{3}$) δ 211.6, 211.1 (minor, major, d, $^{1}J_{PC}$ = 44.3, 42.4 Hz), 163.9, 158.9, 150.6, 150.4, 144.28, 144.22, 137.4 (all 4°), 137.17, 136.9 (4°), 136.3, 135.9, 135.4, 135.3, 135.22 (4°), 135.19 (4°), 135.18 (4°), 134.49, 134.41, 130.20, 130.13, 129.19, 129.16, 128.44, 128.37, 128.31, 128.19, 128.13, 127.38, 113.47 ($MeOC_{6}H_{4}$ 6.84-6.81), 113.42 ($MeOC_{6}H_{4}$ 6.84-6.81), 113.41 ($MeOC_{6}H_{4}$ 6.84-6.81), 111.67 (4°), 111.62 (4°), 111.4 (4°), 111.20 (4°), 111.16 (4°), 87.7, 87.3, 86.1, 85.8, 85.5, 85.4, 84.9 (C_{H1}'), 84.7 (C_{H1}'), 84.6 (C_{H1}'), 71.8, 71.6, 68.6, 68.5, 66.95, 66.91, 63.29 ($^{T1}CH_{5}'$), 63.17 ($^{T1}CH_{5}'$), 62.2 (CH_{5}' of 3′-TBS-OT), 55.39 (MeO), 55.37 (MeO), 40.67 (CH_{2}'), 40.58 (CH_{2}'), 39.94, 25.8 (*t*-Bu), 18.0 (*t*-Bu 4° C), 12.59, 12.56 ($^{T2}CH_{3}$C-5), 11.9, 11.8 ($^{T1}CH_{3}$C-5), -4.52, -4.59, -4.71, -4.76 ($CH_{3}Si$); ^{31}P NMR (202 MHz, $CDCl_{3}$) δ 151.5, 150.2 (46:54); IR ($CDCl_{3}$) 3395, 2955, 2932, 1690 cm^{-1}. HRMS (ESI): Calcd for $C_{54}H_{63}N_{4}NaO_{13}PSi^{+}$ $[M+Na]^{+}$ 1057.37907, found 1057.37949.

Conversion of 12 and 13 to 17. Sulfurization of **13** occurred by reaction in $CD_{3}CN$ with excess phenylacetyl disulfide (PADS) [10] or DDTT [11] and gave new peaks at 66.4 and 66.0 ppm in the ^{31}P NMR spectrum consistent with formation of the tetravalent sulfides. Reaction with 2 M triethylammonium bicarbonate (TEAB) or with bis(trimethylsilyl)acetamide (BSA) [12] followed by TEAB gave material with only one major peak in the ^{31}P NMR spectrum at 113.3 ppm, and it could not be identified.

Oxidation of **13** in acetonitrile with anhydrous 3.3 M *tert*-butyl hydroperoxide [13] gave two peaks in the ^{31}P NMR spectrum for the diastereomeric oxides at -0.9 and -1.1 ppm. Treatment with TEAB gave the *H*-phosphonate (^{31}P NMR ($CDCl_3$): 8.8, 7.5 ppm [12]), and treatment with PADS gave the diastereomeric phosphorothioates (^{31}P NMR ($CDCl_3$): 58.0, 57.9 ppm [12]).

Oxidation of **12** in the same manner gave the diastereomeric oxides (^{31}P NMR ($CDCl_3$): -2.5, -2.7 ppm), and TEAB and PADS gave the same *H*-phosphonate and phosphorothioate spectra as for **13**.

Thermal decomposition of 12 and 13. Samples of **12** or **13** were dissolved in acetonitrile and added to a one-piece teflon vacuum stopcock-sealed heavy-walled glass vessel. The vessel was evacuated using a vacuum pump and then heated in a thermostatted oil bath. Periodically the solvent was removed under vacuum and the contents analyzed by NMR after extraction into $CDCl_3$ solution; after adding the NMR sample back to the vessel, the solvent was once again removed under vacuum and replaced with acetonitrile. Two samples of **12** were examined. A 1:1 mixture of diastereomers was heated for 12 h at 50 °C followed by 4 h at 75 °C, with no change in diastereomer ratio but extensive decomposition to unidentified materials that exhibited very broad bands in the ^{1}H and particularly the ^{31}P NMR spectra. A 42:58 sample of diastereomers was heated sequentially for 1.5 h at 100 °C, 1.5 h at 130°C, and 1.5 h at 150 °C. No change in diastereomer ratio occurred, but decomposition was nearly complete at the end. One sample of **13** was heated, for 2.5 h at 75 °C and 6 h at 100 °C, with no change in the 1:1 diastereomer ratio, and again with nearly complete decomposition.

X-ray structure of 9. A yellow fragment of **9** with approximate dimensions 0.31 mm x 0.37 mm x 0.41 mm, cleaved from a large crystal obtained by slow cooling of a saturated hexanes solution at –35 °C, was used for the X-ray crystallographic analysis. The X-ray intensity data were measured on a Bruker Smart Breeze CCD system equipped with a graphite monochromator at 100(2) K, cooled by an Oxford Cryosystems 700 Series Cryostream. A total of 1464 frames were collected. The total exposure time was 12.20 hours. The frames were integrated with the Bruker SAINT software package using a narrow-frame algorithm. The integration of the data using a monoclinic unit cell yielded a total of 21786 reflections to a maximum θ angle of 27.10° (0.78 Å resolution), of which 4311 were independent (average redundancy 5.054, completeness = 100.0%, R_{int} = 2.00%, R_{sig} = 1.47%) and 3921 (90.95%) were greater than $2\sigma(F^2)$. The final cell constants of a = 15.8768(8) Å, b = 9.2589(5) Å, c = 14.0614(7) Å, β = 108.8770(10)°, volume = 1955.87(17) $Å^3$, were based upon the refinement of the XYZ-centroids of 9998 reflections above 20 σ(I) with 5.168° < 2θ < 54.18°. Data were corrected for absorption effects using the numerical method (SADABS). The ratio of minimum to maximum apparent transmission was 0.927. The calculated minimum and maximum transmission coefficients (based on crystal size) were 0.9430 and 0.9550.

The structure was solved and refined using the Bruker SHELXTL Software Package, using the space group $P2_1/c$, with Z = 4 for the formula unit, $C_{19}H_{33}N_2OP$. The final anisotropic full-matrix least-squares refinement on F^2 with 249 variables converged at R_1 = 3.11%, for the observed data and wR_2 = 8.21% for all data. The goodness-of-fit was 1.048. The largest peak in the final difference electron density synthesis was 0.412

$e^-/Å^3$ and the largest hole was -0.202 $e^-/Å^3$ with an RMS deviation of 0.041 $e^-/Å^3$. On the basis of the final model, the calculated density was 1.143 g/cm^3 and F(000), 736 e^-.

Cambridge Crystallographic Data Centre deposition number for **9**: CCDC 1030743. The data can be obtained free from Cambridge Crystallographic Data Centre via http://www.ccdc.cam.ac.uk/data_request/cif.

Calculations. All geometry and NMR calculations were carried out using *Gaussian 09 Revision A.02* and *D.01* [14] by first carrying out a geometry optimization (DFT, 6-31G(d), B3LYP) with modeling of solvation in chloroform using the polarization continuum model (IEFPCM), with calculation of vibrational frequencies to insure the finding of an energy minimum; NMR calculations (GIAO) were then carried out on the optimized structures using the 6-311+G(2d,p) basis set and the same IEFPCM solvation method.

Each of the inversion barrier calculations was carried out by optimizing using the 6-31+G(d) basis set, but without solvation modeling, with the exception of the phosphite triester where acetonitrile modeling was used. Following optimization, one of the groups was rotated to give the inverted structure, which was then reoptimized; **18** and **19** gave back the same structures but in the opposite configuration at phosphorus. For the transition state calculations, one enantiomer of each optimized structure was converted to a trigonal planar structure, and then used as the starting point for the transition state search. Both the QST3 option in *Gaussian* as well as the simpler Berny TS option ts=(opt,estmfc,noeigentest) described by the Collum group [15] were successfully used. The reported barriers are the smaller barrier from each ground state, as the sum of the electronic and thermal free energies at 298.15 K.

References

1. Gottlieb, H. E.; Kotlyar, V.; Nudelman, A., *J. Org. Chem.* **1997**, *62*, 7512-7515.
2. Fulmer, G. R.; Miller, A. J. M.; Sherden, N. H.; Gottlieb, H. E.; Nudelman, A.; Stoltz, B. M.; Bercaw, J. E.; Goldberg, K. I., *Organometallics* **2010**, *29*, 2176-2179.
3. Williams, D. B. G.; Lawton, M., *J. Org. Chem.* **2010**, *75*, 8351-8354.
4. King, R. B.; Sundaram, P. M., *J. Org. Chem.* **1984**, *49*, 1784-1789.
5. Dellinger, D. J.; Sheehan, D. M.; Christensen, N. K.; Lindberg, J. G.; Caruthers, M. H., *J. Am. Chem. Soc.* **2003**, *125*, 940-950.
6. Xie, C.; Staszak, M. A.; Quatroche, J. T.; Sturgill, C. D.; Khau, V. V.; Martinelli, M. J., *Org. Process Res. Dev.* **2005**, *9*, 730-737.
7. Schaller, H.; Weimann, G.; Lerch, B.; Khorana, H. G., *J. Am. Chem. Soc.* **1963**, *85*, 3821-3827.
8. Ogilvie, K. K.; Thompson, E. A.; Quilliam, M. A.; Westmore, J. B., *Tetrahedron Lett.* **1974**, 2865-2868.
9. Ogilvie, K. K., *Can. J. Chem.* **1973**, *51*, 3799-3807.
10. Cheruvallath, Z. S.; Carty, R. L.; Moore, M. N.; Capaldi, D. C.; Krotz, A. H.; Wheeler, P. D.; Turney, B. J.; Craig, S. R.; Gaus, H. J.; Scozzari, A. N.; Cole, D. L.; Ravikumar, V. T., *Org. Process Res. Dev.* **2000**, *4*, 199-204.
11. Guzaev, A. P., *Tetrahedron Lett.* **2011**, *52*, 434-437.
12. Mukhlall, J. A.; Hersh, W. H., *Nucleosides, Nucleotides Nucleic Acids* **2011**, *30*, 706-725.
13. Hill, J. G.; Rossiter, B. E.; Sharpless, K. B., *J. Org. Chem.* **1983**, *48*, 3607-3608.
14. Frisch, M. J.; Trucks, G. W.; Schlegel, H. B.; Scuseria, G. E.; Robb, M. A.; Cheeseman, J. R.; Scalmani, G.; Barone, V.; Mennucci, B.; Petersson, G. A.; Nakatsuji, H.; Caricato, M.; Li, X.; Hratchian, H. P.; Izmaylov, A. F.; Bloino, J.; Zheng, G.; Sonnenberg, J. L.; Hada, M.; Ehara, M.; Toyota, K.; Fukuda, R.; Hasegawa, J.; Ishida, M.; Nakajima, T.; Honda, Y.; Kitao, O.; Nakai, H.; Vreven, T.; Montgomery Jr., J. A.; Peralta, J. E.; Ogliaro, F.; Bearpark, M.; Heyd, J. J.; Brothers, E.; Kudin, K. N.; Staroverov, V. N.; Keith, T.; Kobayashi, R.; Normand, J.; Raghavachari, K.; Rendell, A.; Burant, J. C.; Iyengar, S. S.; Tomasi, J.; Cossi, M.; Rega, N.; Millam, J. M.; Klene, M.; Knox, J. E.; Cross, J. B.; Bakken, V.; Adamo, C.; Jaramillo, J.; Gomperts, R.; Stratmann, R. E.; Yazyev, O.; Austin, A. J.; Cammi, R.; Pomelli, C.; Ochterski, J. W.; Martin, R. L.; Morokuma, K.; Zakrzewski, V. G.; Voth, G. A.; Salvador, P.; Dannenberg, J. J.; Dapprich, S.; Daniels, A. D.; Farkas, O.; Foresman, J. B.; Ortiz, J. V.; Cioslowski, J.; Fox, D. J. *Gaussian 09, Revision D.01*, Gaussian, Inc.: Wallingford, CT, 2009.
15. Collum, D. B. Electronic Structure calculations in *Gaussian*. http://collum.chem.cornell.edu/documents/Gaussian_optimization.pdf (accessed October 29, 2014).

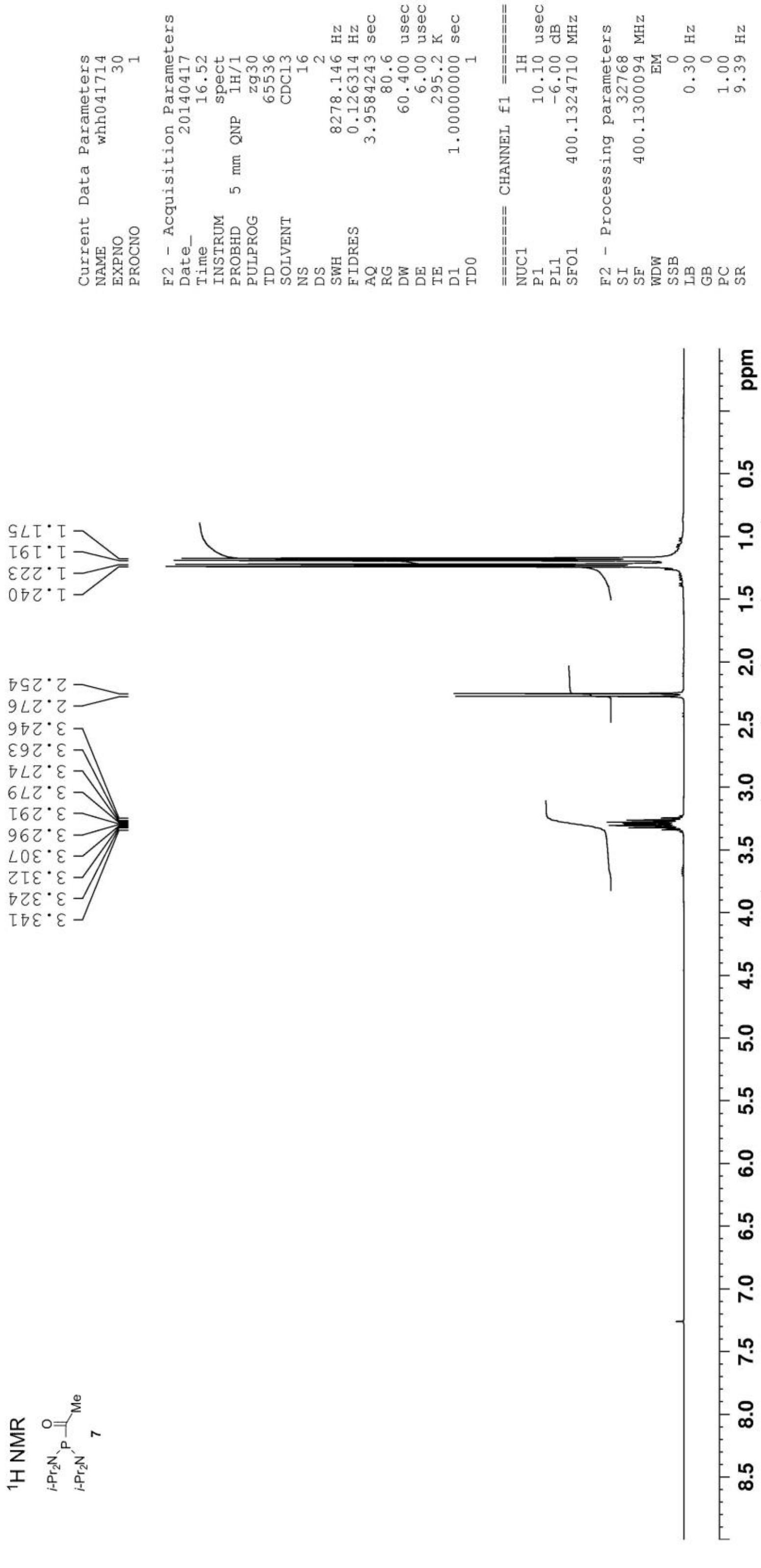

1H NMR
i-Pr2N
i-Pr2N
P
O
Me
7
1.175
1.191
1.223
1.240
2.254
2.276
3.246
3.263
3.274
3.279
3.291
3.296
3.307
3.312
3.324
3.341
6.30
0.64
1.00
ppm
Current Data Parameters
NAME whh041714
EXPNO 30
PROCNO 1
F2 - Acquisition Parameters
Date_ 20140417
Time 16.52
INSTRUM spect
PROBHD 5 mm QNP 1H/1
PULPROG zg30
TD 65536
SOLVENT CDCl3
NS 16
DS 2
SWH 8278.146 Hz
FIDRES 0.126314 Hz
AQ 3.9584243 sec
RG 80.6
DW 60.400 usec
DE 6.00 usec
TE 295.2 K
D1 1.00000000 sec
TD0 1
======== CHANNEL f1 ========
NUC1 1H
P1 10.10 usec
PL1 -6.00 dB
SFO1 400.1324710 MHz
F2 - Processing parameters
SI 32768
SF 400.1300094 MHz
WDW EM
SSB 0
LB 0.30 Hz
GB 0
PC 1.00
SR 9.39 Hz

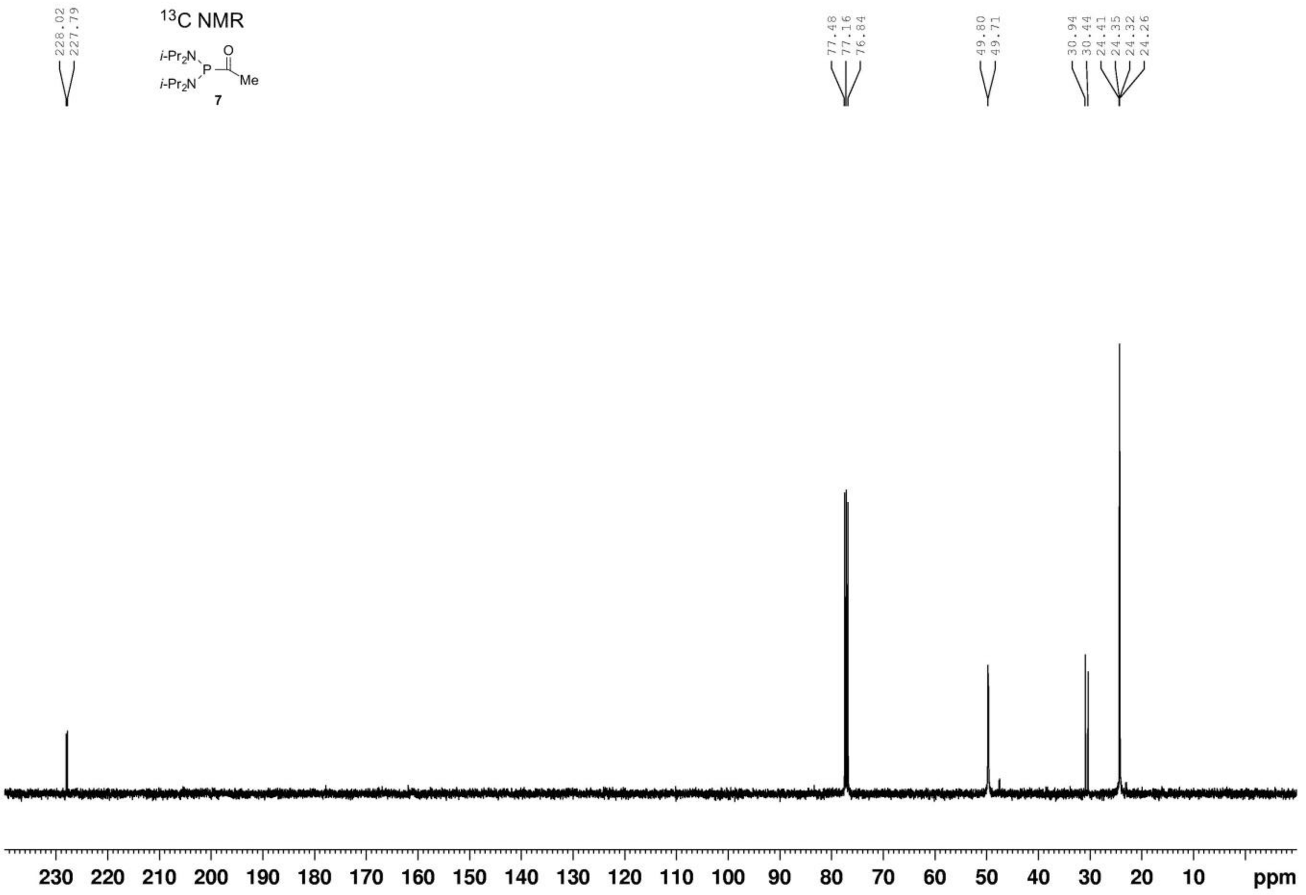


```
Current Data Parameters
NAME          whh041714
EXPNO                32
PROCNO                1

F2 - Acquisition Parameters
Date_          20140417
Time              17.32
INSTRUM           spect
PROBHD   5 mm QNP  1H/1
PULPROG          zgpg30
TD                65536
SOLVENT           CDCl3
NS                  512
DS                    4
SWH           28248.588 Hz
FIDRES         0.431039 Hz
AQ            1.1600372 sec
RG                143.7
DW               17.700 usec
DE                 6.00 usec
TE                295.2 K
D1           2.00000000 sec
d11          0.03000000 sec
DELTA        1.89999998 sec
TD0                   1

======== CHANNEL f1 ========
NUC1                13C
P1                 7.10 usec
PL1               -6.00 dB
SFO1        100.6238364 MHz

======== CHANNEL f2 ========
CPDPRG2         waltz16
NUC2                 1H
PCPD2            100.00 usec
PL2               -6.00 dB
PL12              13.91 dB
PL13             120.00 dB
SFO2        400.1316005 MHz

F2 - Processing parameters
SI                32768
SF          100.6127546 MHz
WDW                  EM
SSB                   0
LB                 1.00 Hz
GB                    0
PC                 1.40
SR               -14.44 Hz
```

^{31}P NMR

7

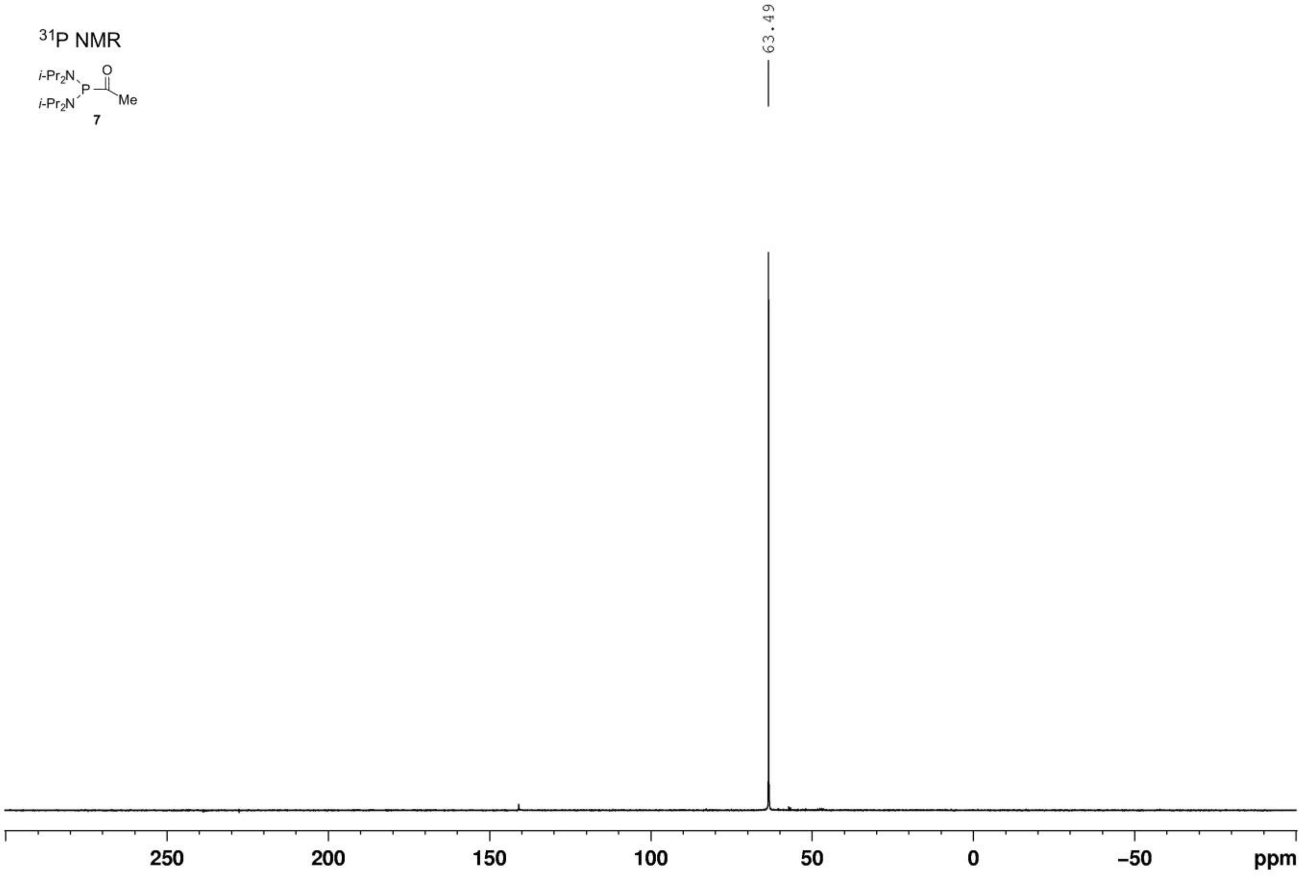


```
Current Data Parameters
NAME          whh041714
EXPNO                31
PROCNO                1

F2 - Acquisition Parameters
Date_          20140417
Time              17.04
INSTRUM           spect
PROBHD   5 mm QNP  1H/1
PULPROG          zgpg30
TD                65536
SOLVENT           CDCl3
NS                  256
DS                    4
SWH           64935.066 Hz
FIDRES         0.990830 Hz
AQ            0.5046772 sec
RG                 3251
DW                7.700 usec
DE                 6.00 usec
TE                295.2 K
D1           2.00000000 sec
d11          0.03000000 sec
DELTA        1.89999998 sec
TD0                   1

======== CHANNEL f1 ========
NUC1                31P
P1                 4.65 usec
PL1               -6.00 dB
SFO1        161.9917906 MHz

======== CHANNEL f2 ========
CPDPRG2         waltz16
NUC2                 1H
PCPD2            100.00 usec
PL2               -6.00 dB
PL12              13.91 dB
PL13             120.00 dB
SFO2        400.1316005 MHz

F2 - Processing parameters
SI                32768
SF          161.9755358 MHz
WDW                  EM
SSB                   0
LB                 1.00 Hz
GB                    0
PC                 1.40
SR               -57.24 Hz
```

2D-HETCOR

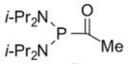
i-Pr2N
i-Pr2N
P
O
Me

7

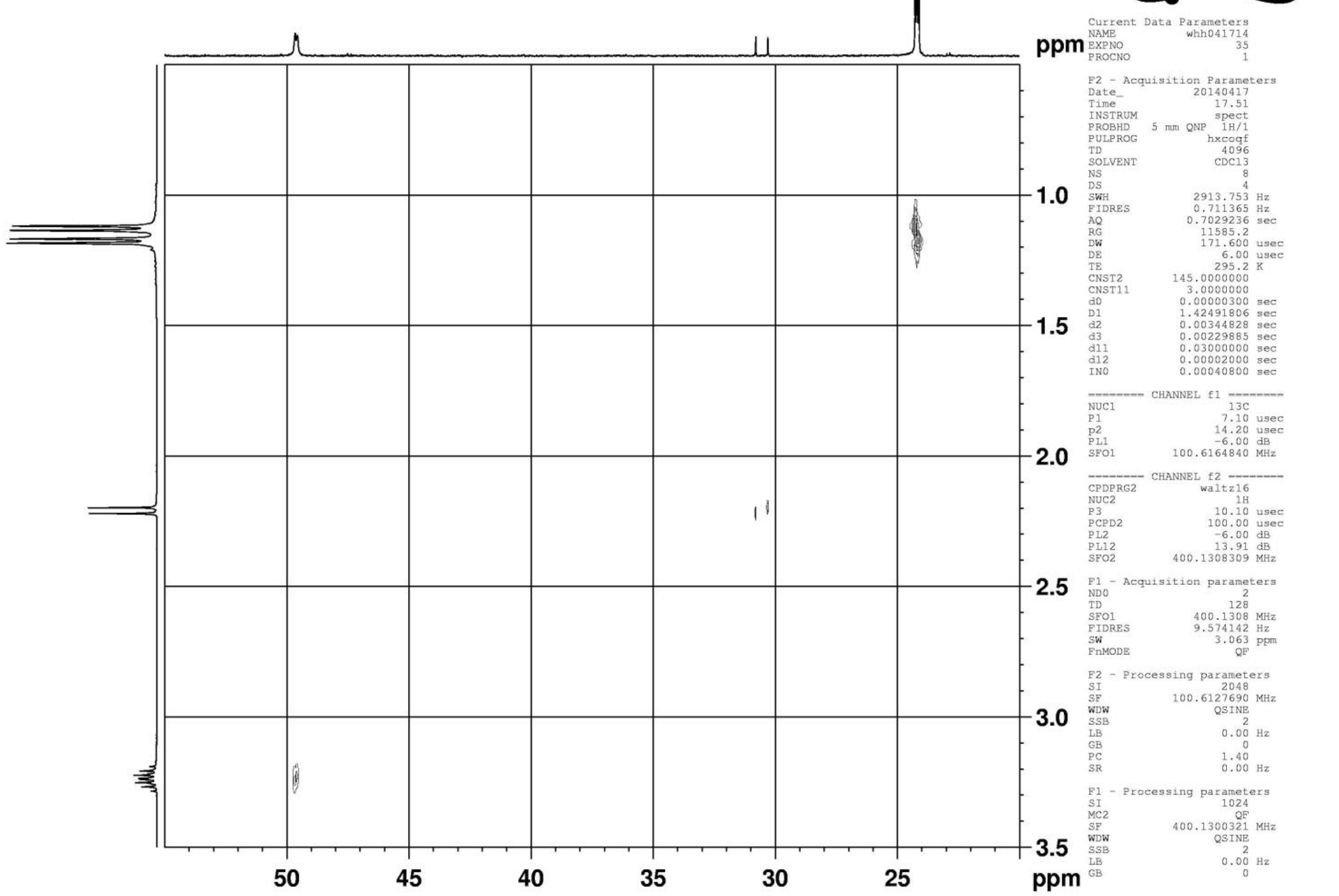
BRUKER
Current Data Parameters
NAME whh041714
EXPNO 35
PROCNO 1
F2 - Acquisition Parameters
Date_ 20140417
Time 17.51
INSTRUM spect
PROBHD 5 mm QNP 1H/1
PULPROG hxcoqf
TD 4096
SOLVENT CDCl3
NS 8
DS 4
SWH 2913.753 Hz
FIDRES 0.711365 Hz
AQ 0.7029236 sec
RG 11585.2
DW 171.600 usec
DE 6.00 usec
TE 295.2 K
CNST2 145.0000000
CNST11 3.0000000
d0 0.00000300 sec
D1 1.42491806 sec
d2 0.00344828 sec
d3 0.00229885 sec
d11 0.03000000 sec
d12 0.00002000 sec
IN0 0.00040800 sec
======== CHANNEL f1 ========
NUC1 13C
P1 7.10 usec
p2 14.20 usec
PL1 -6.00 dB
SFO1 100.6164840 MHz
======== CHANNEL f2 ========
CPDPRG2 waltz16
NUC2 1H
P3 10.10 usec
PCPD2 100.00 usec
PL2 -6.00 dB
PL12 13.91 dB
SFO2 400.1308309 MHz
F1 - Acquisition parameters
ND0 2
TD 128
SFO1 400.1308 MHz
FIDRES 9.574142 Hz
SW 3.063 ppm
FnMODE QF
F2 - Processing parameters
SI 2048
SF 100.6127690 MHz
WDW QSINE
SSB 2
LB 0.00 Hz
GB 0
PC 1.40
SR 0.00 Hz
F1 - Processing parameters
SI 1024
MC2 QF
SF 400.1300321 MHz
WDW QSINE
SSB 2
LB 0.00 Hz
GB 0
ppm
1.0
1.5
2.0
2.5
3.0
3.5
ppm
50
45
40
35
30
25
ppm

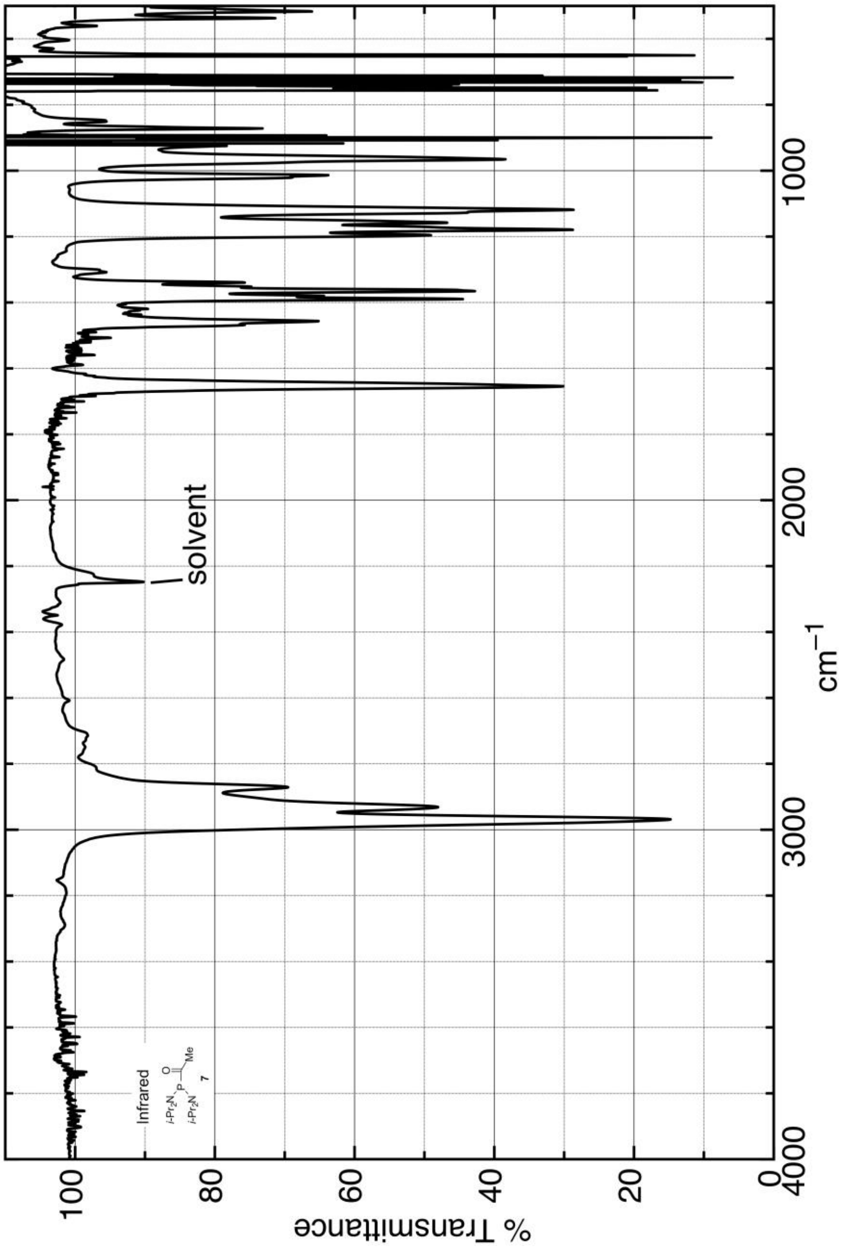
Infrared
7
solvent
cm−1
% Transmittance
100
80
60
40
20
0
4000
3000
2000
1000

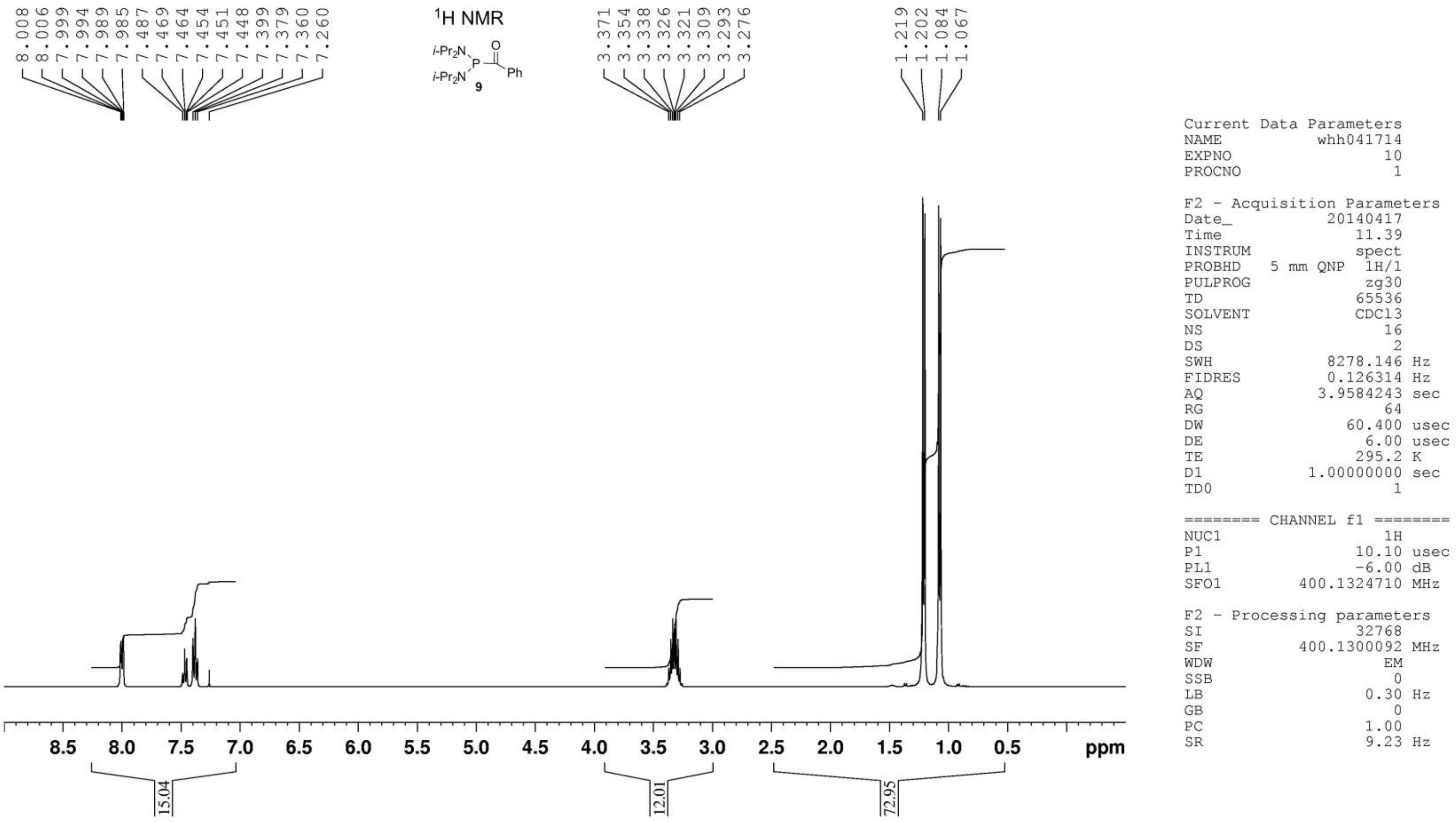

1H NMR
i-Pr2N
i-Pr2N
P
O
Ph
9
8.008
8.006
7.999
7.994
7.989
7.985
7.487
7.469
7.464
7.454
7.451
7.448
7.399
7.379
7.360
7.260
3.371
3.354
3.338
3.326
3.321
3.309
3.293
3.276
1.219
1.202
1.084
1.067
8.5
8.0
7.5
7.0
6.5
6.0
5.5
5.0
4.5
4.0
3.5
3.0
2.5
2.0
1.5
1.0
0.5
ppm
15.04
12.01
72.95
Current Data Parameters
NAME whh041714
EXPNO 10
PROCNO 1
F2 - Acquisition Parameters
Date_ 20140417
Time 11.39
INSTRUM spect
PROBHD 5 mm QNP 1H/1
PULPROG zg30
TD 65536
SOLVENT CDCl3
NS 16
DS 2
SWH 8278.146 Hz
FIDRES 0.126314 Hz
AQ 3.9584243 sec
RG 64
DW 60.400 usec
DE 6.00 usec
TE 295.2 K
D1 1.00000000 sec
TD0 1
======== CHANNEL f1 ========
NUC1 1H
P1 10.10 usec
PL1 -6.00 dB
SFO1 400.1324710 MHz
F2 - Processing parameters
SI 32768
SF 400.1300092 MHz
WDW EM
SSB 0
LB 0.30 Hz
GB 0
PC 1.00
SR 9.23 Hz

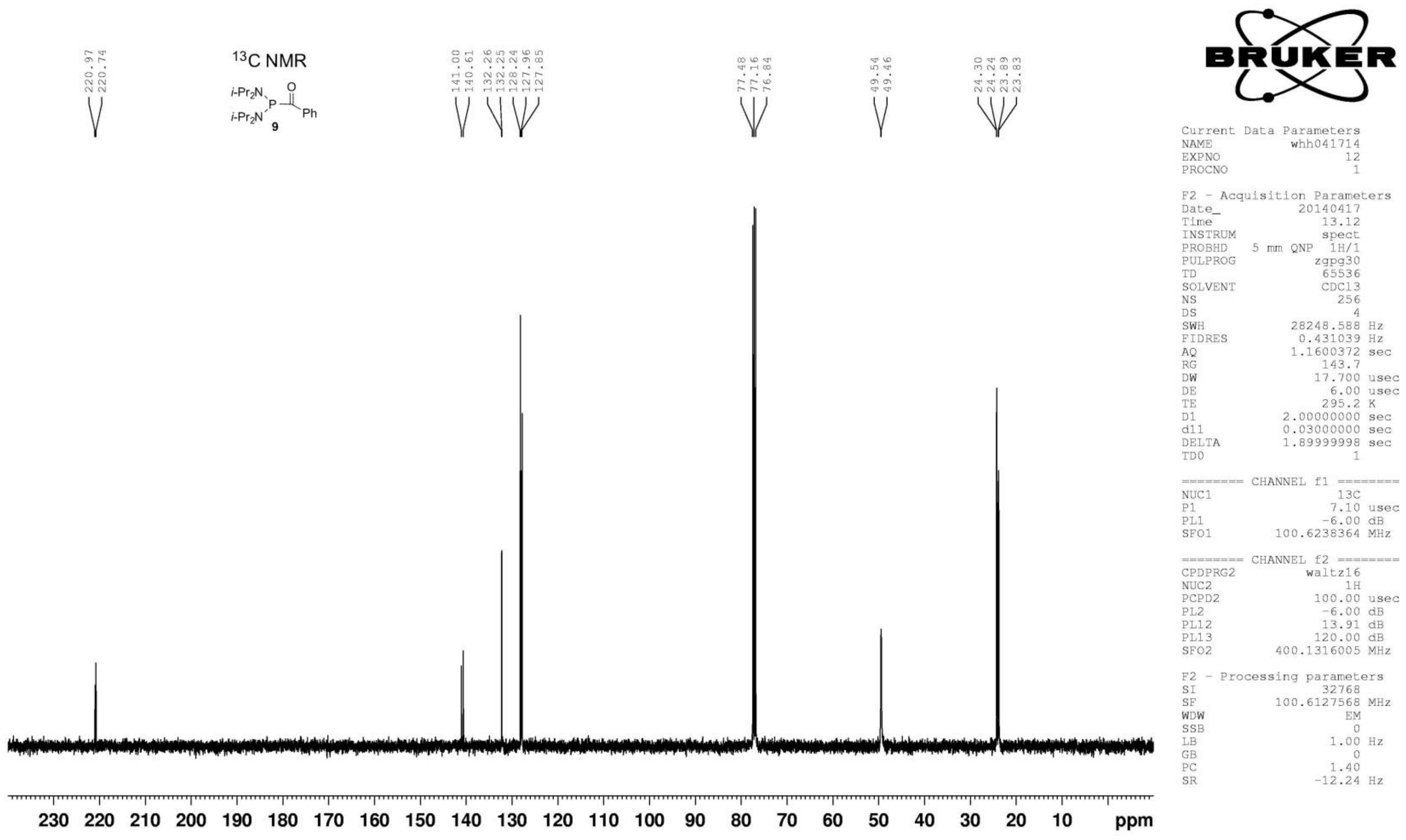
220.97
220.74
13C NMR
i-Pr2N
i-Pr2N
P
O
Ph
9
141.00
140.61
132.26
132.25
128.24
127.96
127.85
77.48
77.16
76.84
49.54
49.46
24.30
24.24
23.89
23.83
BRUKER
Current Data Parameters
NAME whh041714
EXPNO 12
PROCNO 1
F2 - Acquisition Parameters
Date_ 20140417
Time 13.12
INSTRUM spect
PROBHD 5 mm QNP 1H/1
PULPROG zgpg30
TD 65536
SOLVENT CDCl3
NS 256
DS 4
SWH 28248.588 Hz
FIDRES 0.431039 Hz
AQ 1.1600372 sec
RG 143.7
DW 17.700 usec
DE 6.00 usec
TE 295.2 K
D1 2.00000000 sec
d11 0.03000000 sec
DELTA 1.89999998 sec
TD0 1
======== CHANNEL f1 ========
NUC1 13C
P1 7.10 usec
PL1 -6.00 dB
SFO1 100.6238364 MHz
======== CHANNEL f2 ========
CPDPRG2 waltz16
NUC2 1H
PCPD2 100.00 usec
PL2 -6.00 dB
PL12 13.91 dB
PL13 120.00 dB
SFO2 400.1316005 MHz
F2 - Processing parameters
SI 32768
SF 100.6127568 MHz
WDW EM
SSB 0
LB 1.00 Hz
GB 0
PC 1.40
SR -12.24 Hz
230 220 210 200 190 180 170 160 150 140 130 120 110 100 90 80 70 60 50 40 30 20 10 ppm

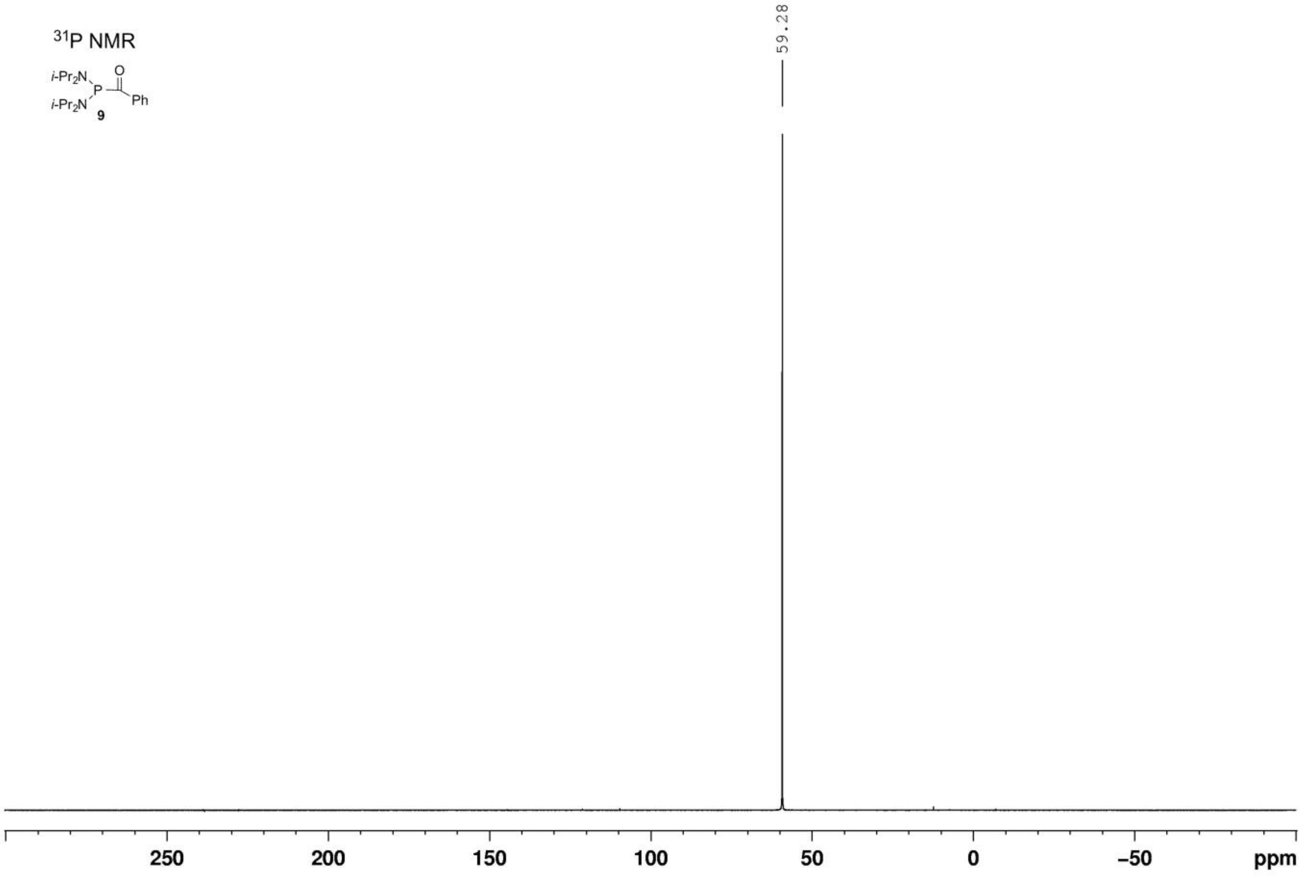


```
Current Data Parameters
NAME              whh041814
EXPNO                    11
PROCNO                    1

F2 - Acquisition Parameters
Date_              20140418
Time                  11.54
INSTRUM               spect
PROBHD   5 mm QNP  1H/1
PULPROG              zgpg30
TD                    65536
SOLVENT               CDCl3
NS                      512
DS                        4
SWH              64935.066 Hz
FIDRES            0.990830 Hz
AQ               0.5046772 sec
RG                  10321.3
DW                    7.700 usec
DE                     6.00 usec
TE                    295.2 K
D1               2.00000000 sec
d11              0.03000000 sec
DELTA            1.89999998 sec
TD0                       1

======== CHANNEL f1 ========
NUC1                    31P
P1                     4.65 usec
PL1                   -6.00 dB
SFO1            161.9917906 MHz

======== CHANNEL f2 ========
CPDPRG2             waltz16
NUC2                     1H
PCPD2                100.00 usec
PL2                   -6.00 dB
PL12                  13.91 dB
PL13                 120.00 dB
SFO2            400.1316005 MHz

F2 - Processing parameters
SI                    32768
SF              161.9755358 MHz
WDW                      EM
SSB                       0
LB                     1.00 Hz
GB                        0
PC                     1.40
SR                   -57.24 Hz
```

2D-HETCOR

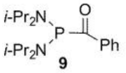

9

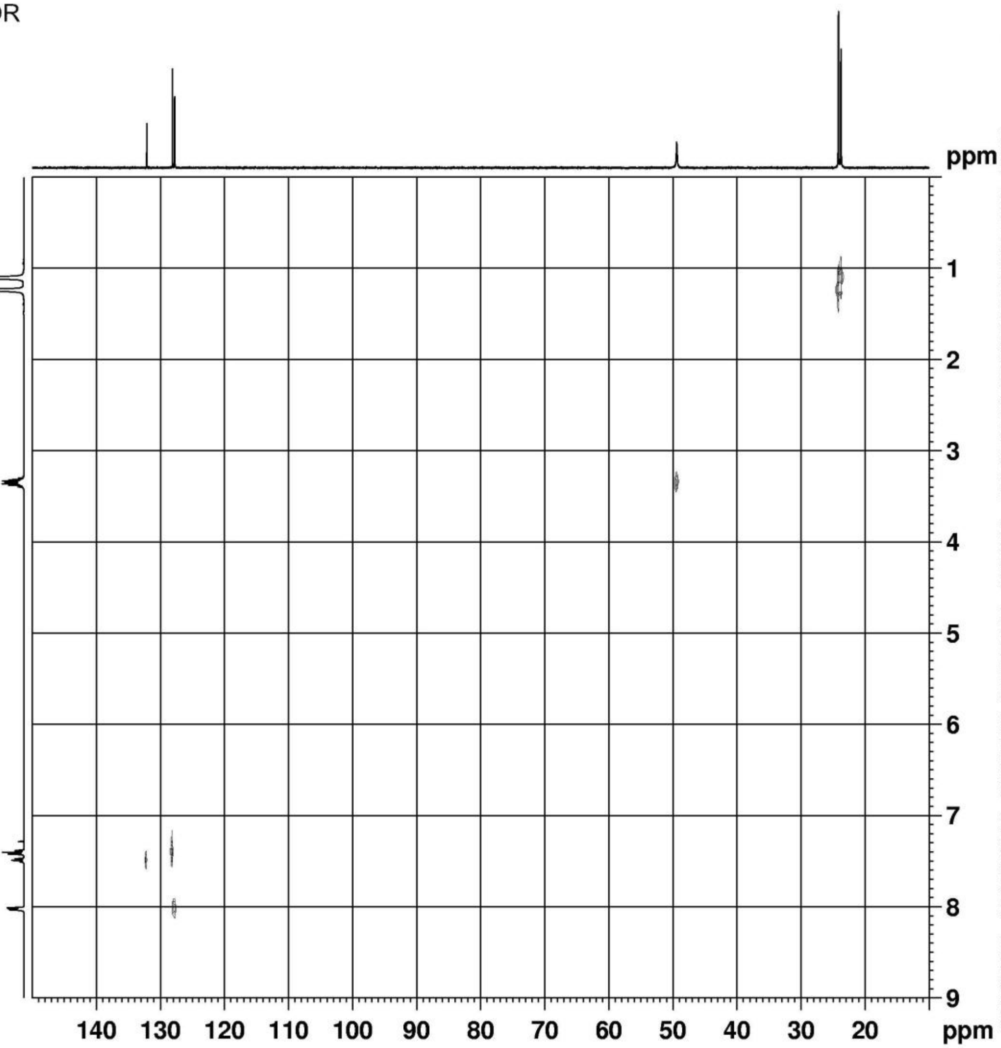

ppm
140 130 120 110 100 90 80 70 60 50 40 30 20
ppm
1 2 3 4 5 6 7 8 9
Current Data Parameters
NAME whh041714
EXPNO 14
PROCNO 1
F2 - Acquisition Parameters
Date_ 20140417
Time 15.45
INSTRUM spect
PROBHD 5 mm QNP 1H/1
PULPROG hxcoqf
TD 4096
SOLVENT CDCl3
NS 8
DS 4
SWH 12165.450 Hz
FIDRES 2.970081 Hz
AQ 0.1683956 sec
RG 13004
DW 41.100 usec
DE 6.00 usec
TE 295.2 K
CNST2 145.0000000
CNST11 3.0000000
d0 0.00000300 sec
D1 1.95739806 sec
d2 0.00344828 sec
d3 0.00229885 sec
d11 0.03000000 sec
d12 0.00002000 sec
IN0 0.00014640 sec
======== CHANNEL f1 ========
NUC1 13C
P1 7.10 usec
p2 14.20 usec
PL1 -6.00 dB
SFO1 100.6205882 MHz
======== CHANNEL f2 ========
CPDPRG2 waltz16
NUC2 1H
P3 10.10 usec
PCPD2 100.00 usec
PL2 -6.00 dB
PL12 13.91 dB
SFO2 400.1317527 MHz
F1 - Acquisition parameters
ND0 2
TD 128
SFO1 400.1318 MHz
FIDRES 26.682035 Hz
SW 8.535 ppm
FnMODE QF
F2 - Processing parameters
SI 2048
SF 100.6127690 MHz
WDW QSINE
SSB 2
LB 0.00 Hz
GB 0
PC 1.40
SR 0.00 Hz
F1 - Processing parameters
SI 1024
MC2 QF
SF 400.1300000 MHz
WDW QSINE
SSB 2
LB 0.00 Hz
GB 0

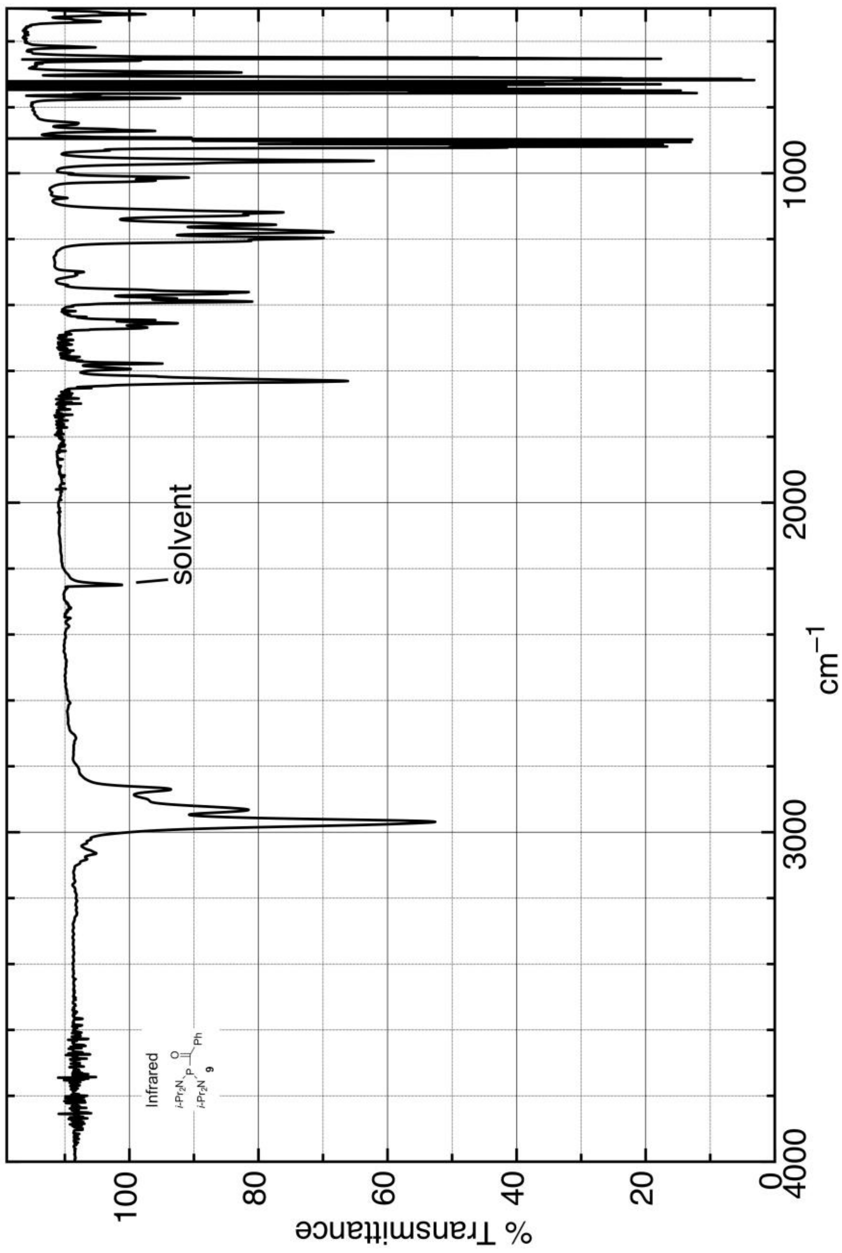
Infrared
9
solvent
% Transmittance
cm−1
0
20
40
60
80
100
4000
3000
2000
1000

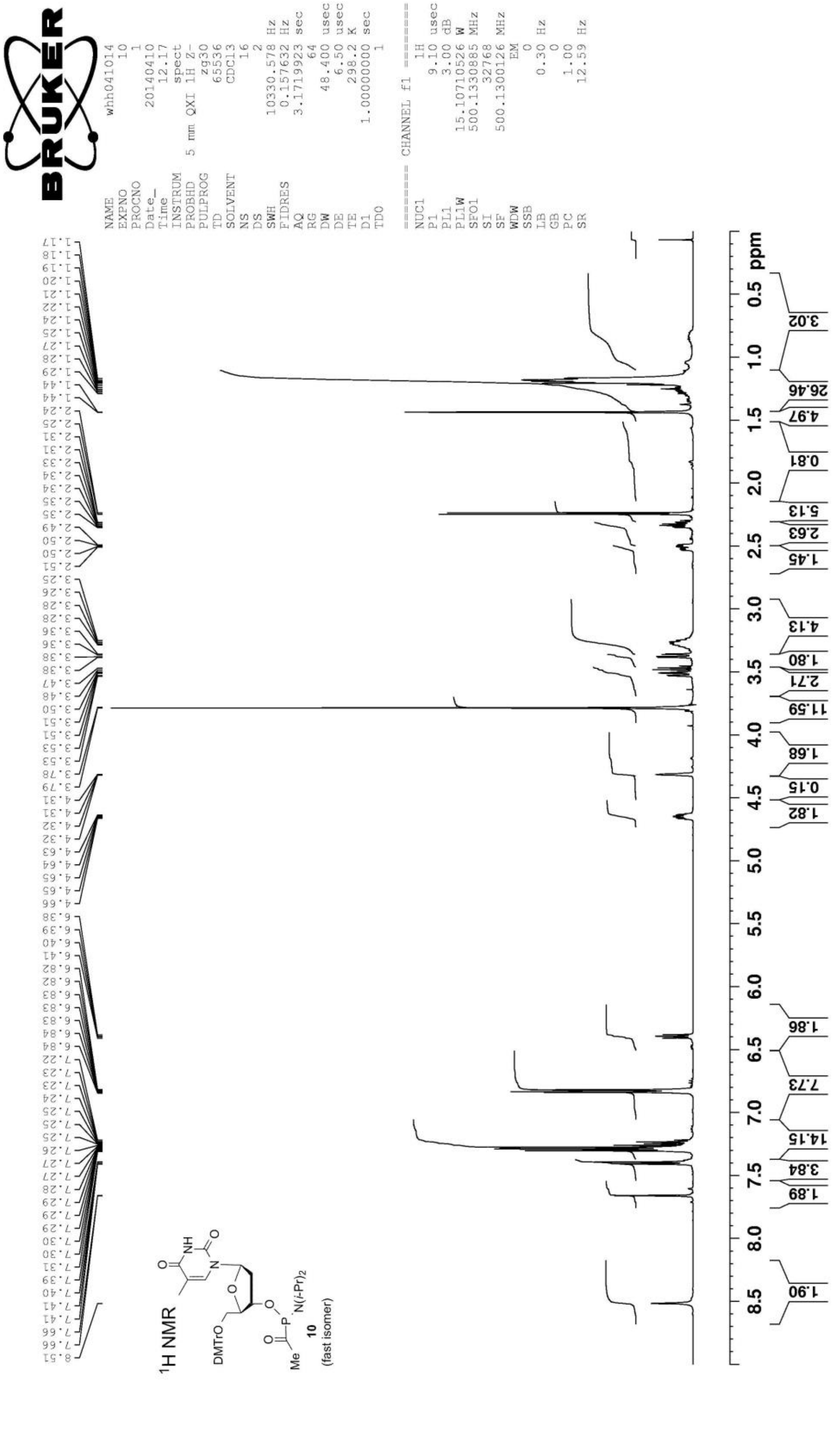

1H NMR
DMTrO
Me
N(i-Pr)2
10
(fast isomer)
NAME whh041014
EXPNO 10
PROCNO 1
Date_ 20140410
Time 12.17
INSTRUM spect
PROBHD 5 mm QXI 1H Z-
PULPROG zg30
TD 65536
SOLVENT CDCl3
NS 16
DS 2
SWH 10330.578 Hz
FIDRES 0.157632 Hz
AQ 3.1719923 sec
RG 64
DW 48.400 usec
DE 6.50 usec
TE 298.2 K
D1 1.00000000 sec
TD0 1
======== CHANNEL f1 ========
NUC1 1H
P1 9.10 usec
PL1 3.00 dB
PL1W 15.10710526 W
SFO1 500.1330885 MHz
SI 32768
SF 500.1300126 MHz
WDW EM
SSB 0
LB 0.30 Hz
GB 0
PC 1.00
SR 12.59 Hz
8.51 7.66 7.66 7.41 7.41 7.40 7.39 7.31 7.30 7.30 7.29 7.29 7.29 7.28 7.27 7.27 7.26 7.25 7.25 7.25 7.24 7.23 7.23 7.22 6.84 6.84 6.83 6.83 6.83 6.82 6.82 6.41 6.40 6.39 6.38 4.66 4.65 4.65 4.64 4.63 4.32 4.32 4.31 4.31 3.79 3.78 3.53 3.53 3.51 3.51 3.50 3.48 3.47 3.38 3.38 3.36 3.36 3.28 3.28 3.26 3.25 2.51 2.50 2.50 2.49 2.35 2.35 2.34 2.34 2.33 2.31 2.31 2.25 2.24 1.44 1.44 1.29 1.28 1.27 1.25 1.24 1.22 1.21 1.20 1.19 1.18 1.17
1.90 1.89 3.84 14.15 7.73 1.86 1.82 0.15 1.68 11.59 2.71 1.80 4.13 1.45 2.63 5.13 0.81 4.97 26.46 3.02
8.5 8.0 7.5 7.0 6.5 6.0 5.5 5.0 4.5 4.0 3.5 3.0 2.5 2.0 1.5 1.0 0.5 ppm

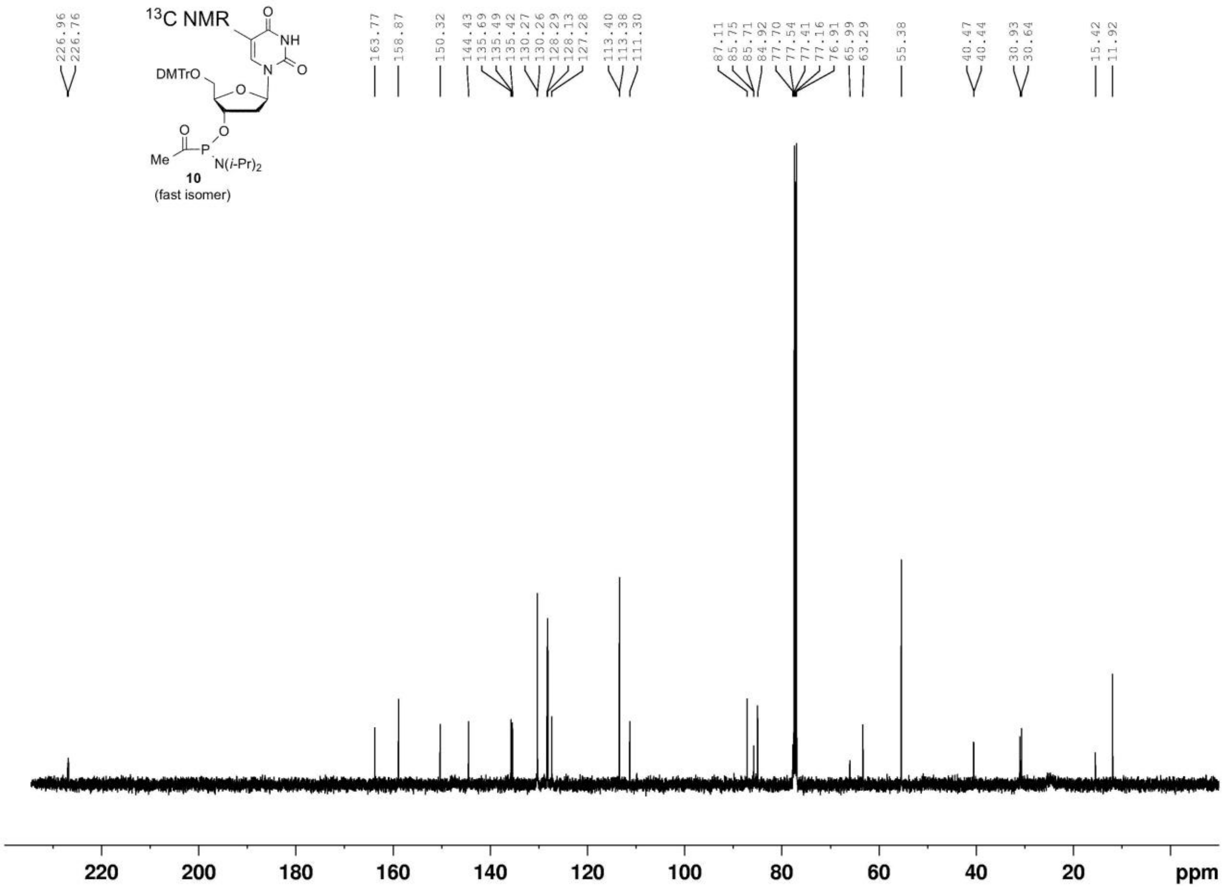

BRUKER

```
NAME          whh041014
EXPNO                11
PROCNO                1
Date_          20140410
Time              13.16
INSTRUM           spect
PROBHD   5 mm QXI 1H Z-
PULPROG         zgpg30
TD                65536
SOLVENT           CDCl3
NS                 1024
DS                    4
SWH           31250.000 Hz
FIDRES         0.476837 Hz
AQ            1.0486259 sec
RG                  575
DW               16.000 usec
DE                 6.50 usec
TE                298.2 K
D1           2.00000000 sec
D11          0.03000000 sec
TD0                   1

======== CHANNEL f1 ========
NUC1                13C
P1                14.50 usec
PL1               -1.00 dB
PL1W       132.81565857 W
SFO1        125.7716224 MHz

======== CHANNEL f2 ========
CPDPRG2         waltz16
NUC2                 1H
PCPD2             87.00 usec
PL2                3.00 dB
PL12              22.00 dB
PL13              22.00 dB
PL2W        15.10710526 W
PL12W        0.19018719 W
PL13W        0.19018719 W
SFO2        500.1320005 MHz
SI                32768
SF          125.7577722 MHz
WDW                  EM
SSB                   0
LB                 1.00 Hz
GB                    0
PC                 1.40
SR               -16.78 Hz
```

^{31}P NMR

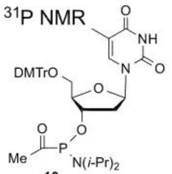

10
(fast isomer)

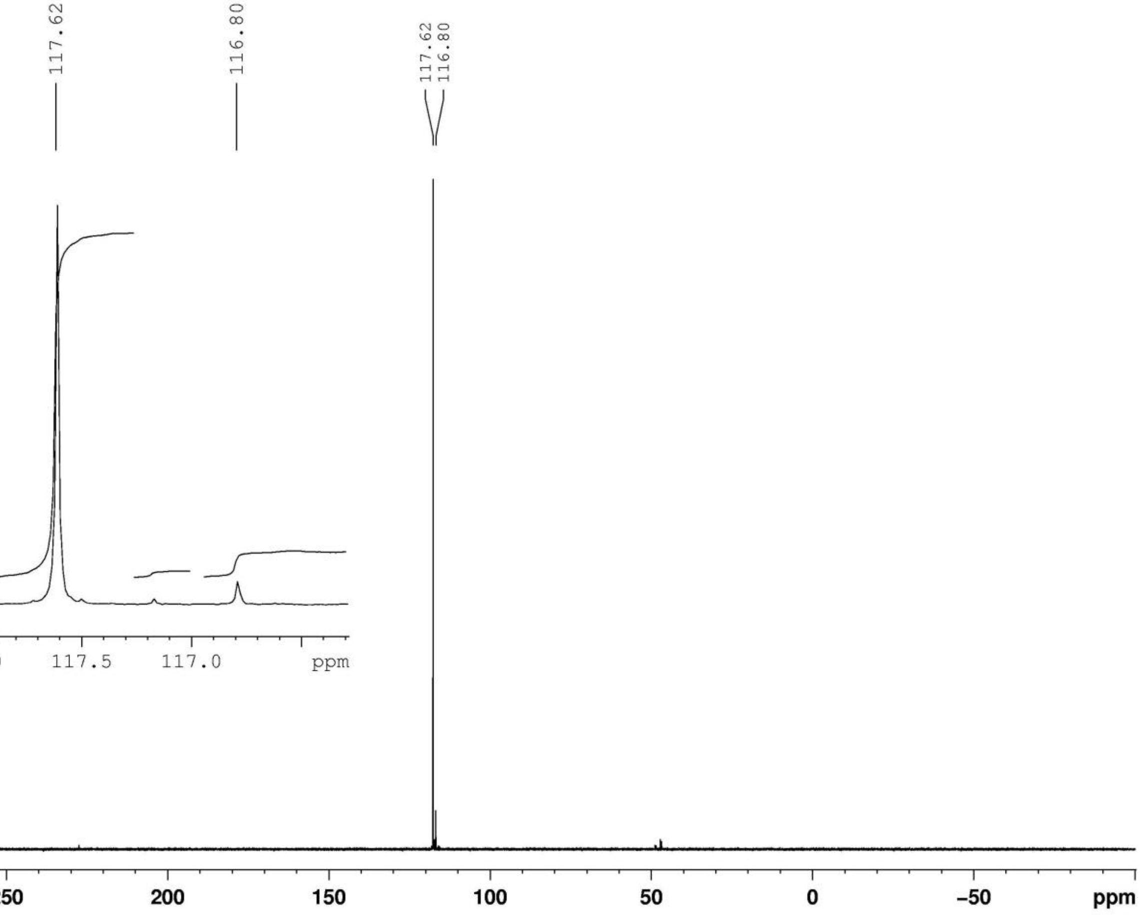


```
Current Data Parameters
NAME          whh040114
EXPNO                11
PROCNO                1

F2 - Acquisition Parameters
Date_          20140401
Time              15.35
INSTRUM           spect
PROBHD   5 mm QNP  1H/1
PULPROG          zgpg30
TD                65536
SOLVENT           CDCl3
NS                  512
DS                    4
SWH           64935.066 Hz
FIDRES         0.990830 Hz
AQ            0.5046772 sec
RG              23170.5
DW                7.700 usec
DE                 6.00 usec
TE                298.2 K
D1           2.00000000 sec
d11          0.03000000 sec
DELTA        1.89999998 sec
TD0                   1

======== CHANNEL f1 ========
NUC1                31P
P1                 4.65 usec
PL1               -6.00 dB
SFO1        161.9917906 MHz

======== CHANNEL f2 ========
CPDPRG2         waltz16
NUC2                 1H
PCPD2            100.00 usec
PL2               -6.00 dB
PL12              13.91 dB
PL13             120.00 dB
SFO2        400.1316005 MHz

F2 - Processing parameters
SI                32768
SF          161.9755358 MHz
WDW                  EM
SSB                   0
LB                 1.00 Hz
GB                    0
PC                 1.40
SR               -57.24 Hz
```

COSY90

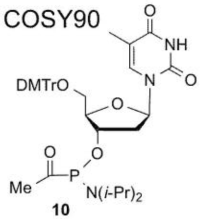

10
(fast isomer)

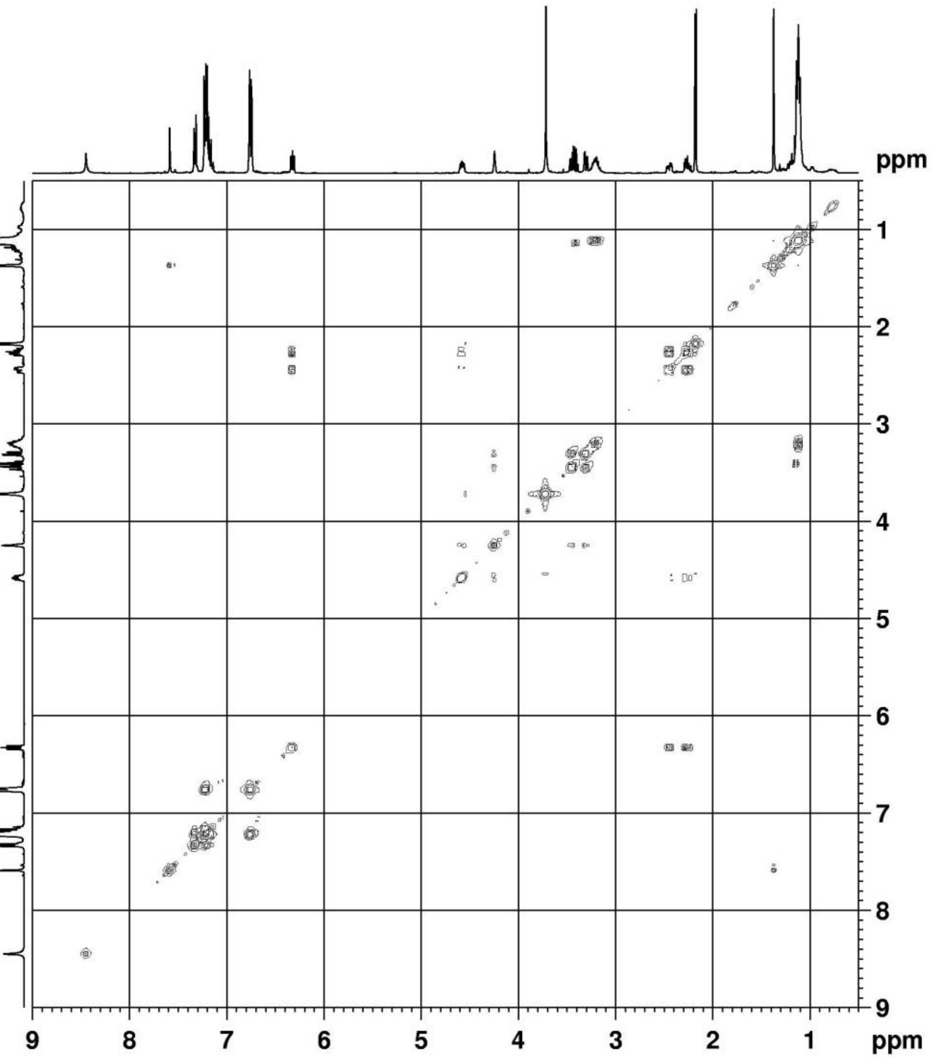


```
Current Data Parameters
NAME            whh041014
EXPNO                  11
PROCNO                  1

F2 - Acquisition Parameters
Date_            20140410
Time                16.35
INSTRUM             spect
PROBHD   5 mm QNP  1H/1
PULPROG          cosygf90
TD                   2048
SOLVENT             CDCl3
NS                      8
DS                      4
SWH              3654.971 Hz
FIDRES           1.784654 Hz
AQ              0.2802164 sec
RG                  161.3
DW                136.800 usec
DE                   6.00 usec
TE                  296.2 K
d0             0.00000300 sec
D1             1.96845996 sec
IN0            0.00027360 sec

======== CHANNEL f1 ========
NUC1                   1H
P1                  10.10 usec
PL1                 -6.00 dB
SFO1          400.1318536 MHz

F1 - Acquisition parameters
ND0                     1
TD                    256
SFO1             400.1319 MHz
FIDRES          14.277229 Hz
SW                  9.134 ppm
FnMODE                 QF

F2 - Processing parameters
SI                   1024
SF            400.1300371 MHz
WDW                  SINE
SSB                     0
LB                   0.00 Hz
GB                      0
PC                   1.40
SR                  37.11 Hz

F1 - Processing parameters
SI                   1024
MC2                    QF
SF            400.1300371 MHz
WDW                  SINE
SSB                     0
LB                   0.00 Hz
GB                      0
```

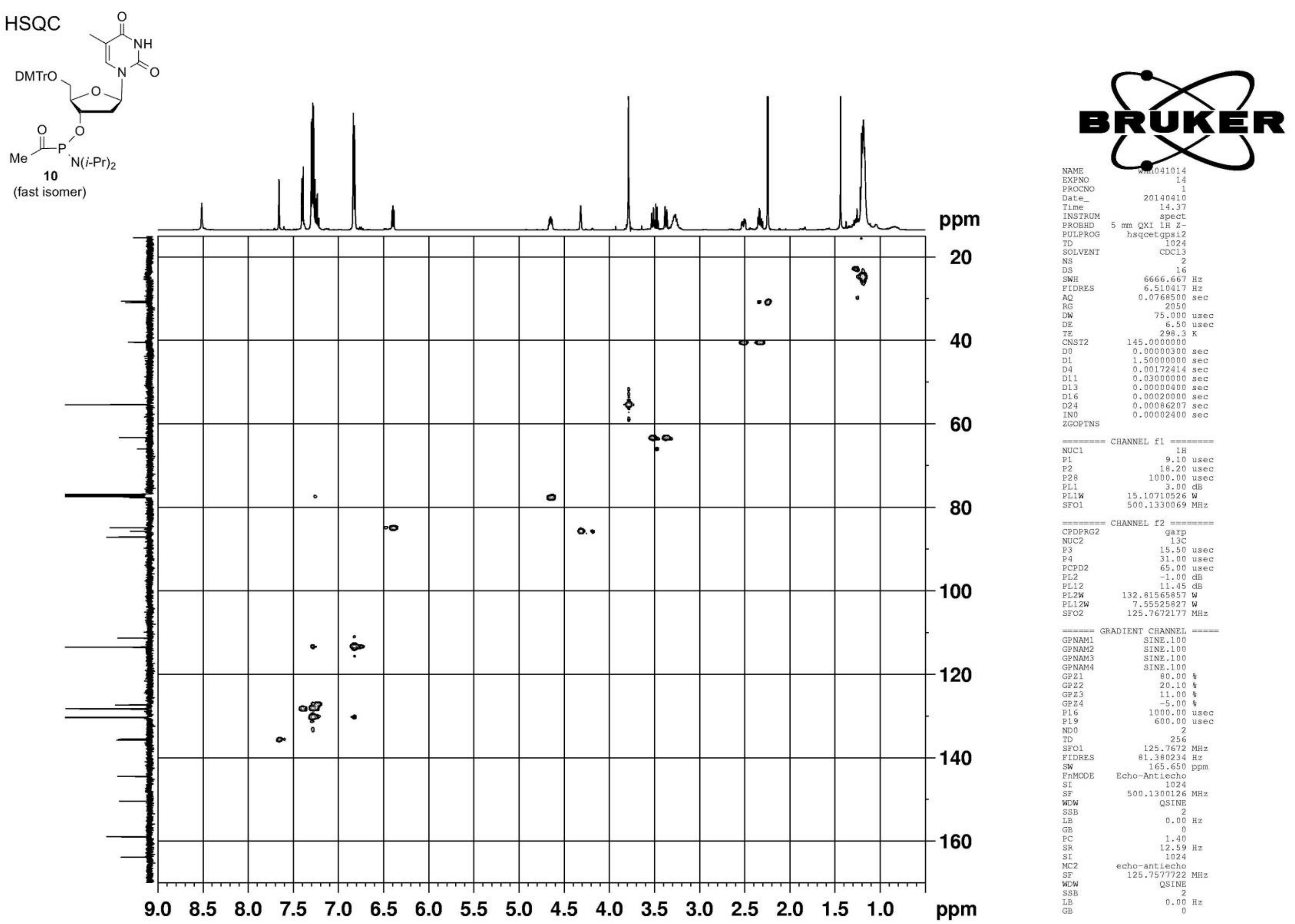
HSQC
DMTrO
O
NH
N
Me
P
N(i-Pr)2
10
(fast isomer)
ppm
20
40
60
80
100
120
140
160
9.0 8.5 8.0 7.5 7.0 6.5 6.0 5.5 5.0 4.5 4.0 3.5 3.0 2.5 2.0 1.5 1.0
BRUKER
NAME wm041014
EXPNO 14
PROCNO 1
Date_ 20140410
Time 14.37
INSTRUM spect
PROBHD 5 mm QXI 1H Z-
PULPROG hsqcetgpsi2
TD 1024
SOLVENT CDCl3
NS 2
DS 16
SWH 6666.667 Hz
FIDRES 6.510417 Hz
AQ 0.0768500 sec
RG 2050
DW 75.000 usec
DE 6.50 usec
TE 298.3 K
CNST2 145.0000000
D0 0.00000300 sec
D1 1.50000000 sec
D4 0.00172414 sec
D11 0.03000000 sec
D13 0.00000400 sec
D16 0.00020000 sec
D24 0.00086207 sec
IN0 0.00002400 sec
ZGOPTNS
======== CHANNEL f1 ========
NUC1 1H
P1 9.10 usec
P2 18.20 usec
P28 1000.00 usec
PL1 3.00 dB
PL1W 15.10710526 W
SFO1 500.1330069 MHz
======== CHANNEL f2 ========
CPDPRG2 garp
NUC2 13C
P3 15.50 usec
P4 31.00 usec
PCPD2 65.00 usec
PL2 -1.00 dB
PL12 11.45 dB
PL2W 132.81565857 W
PL12W 7.55525827 W
SFO2 125.7672177 MHz
====== GRADIENT CHANNEL =====
GPNAM1 SINE.100
GPNAM2 SINE.100
GPNAM3 SINE.100
GPNAM4 SINE.100
GPZ1 80.00 %
GPZ2 20.10 %
GPZ3 11.00 %
GPZ4 -5.00 %
P16 1000.00 usec
P19 600.00 usec
ND0 2
TD 256
SFO1 125.7672 MHz
FIDRES 81.380234 Hz
SW 165.650 ppm
FnMODE Echo-Antiecho
SI 1024
SF 500.1300126 MHz
WDW QSINE
SSB 2
LB 0.00 Hz
GB 0
PC 1.40
SR 12.59 Hz
SI 1024
MC2 echo-antiecho
SF 125.7577722 MHz
WDW QSINE
SSB 2
LB 0.00 Hz
GB 0

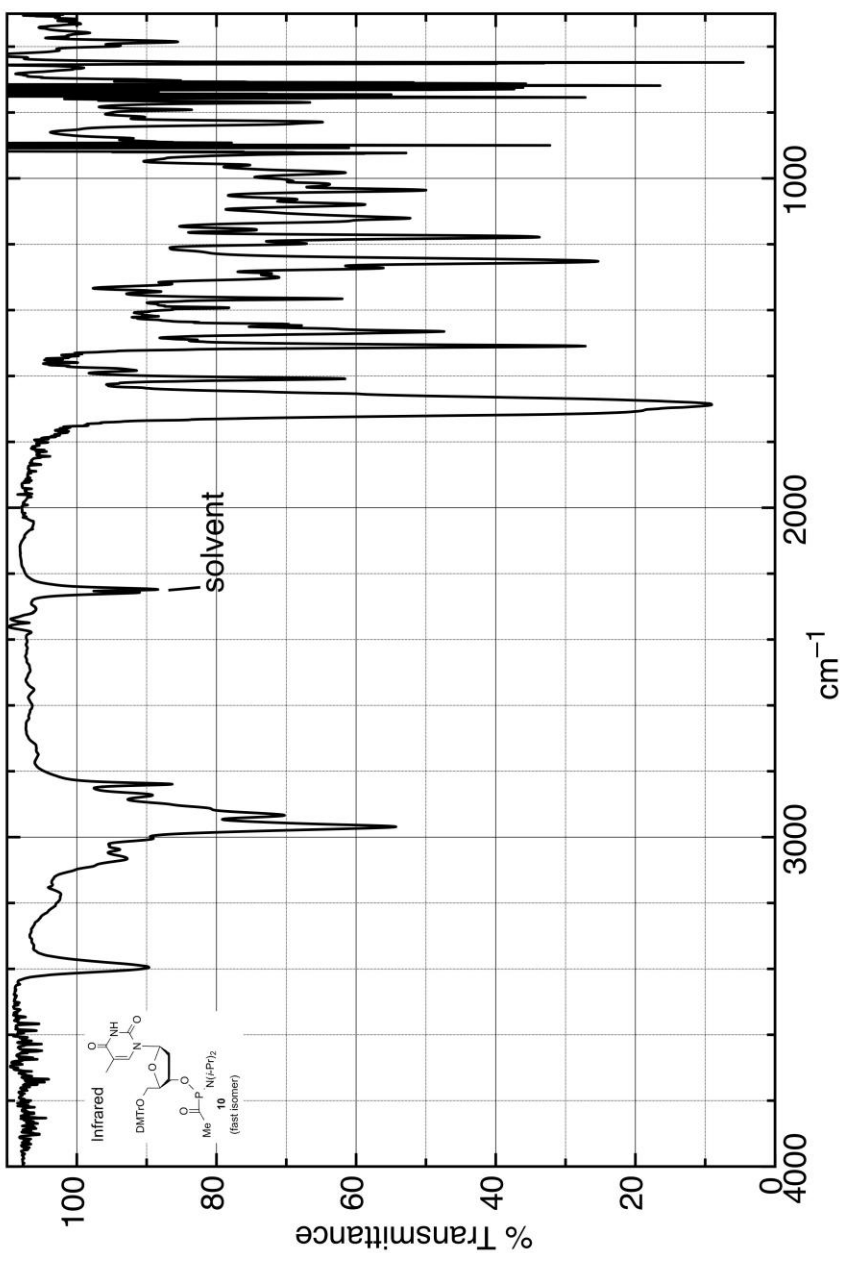

Infrared
DMTrO
NH
Me
N(i-Pr)2
10
(fast isomer)
solvent
% Transmittance
cm−1

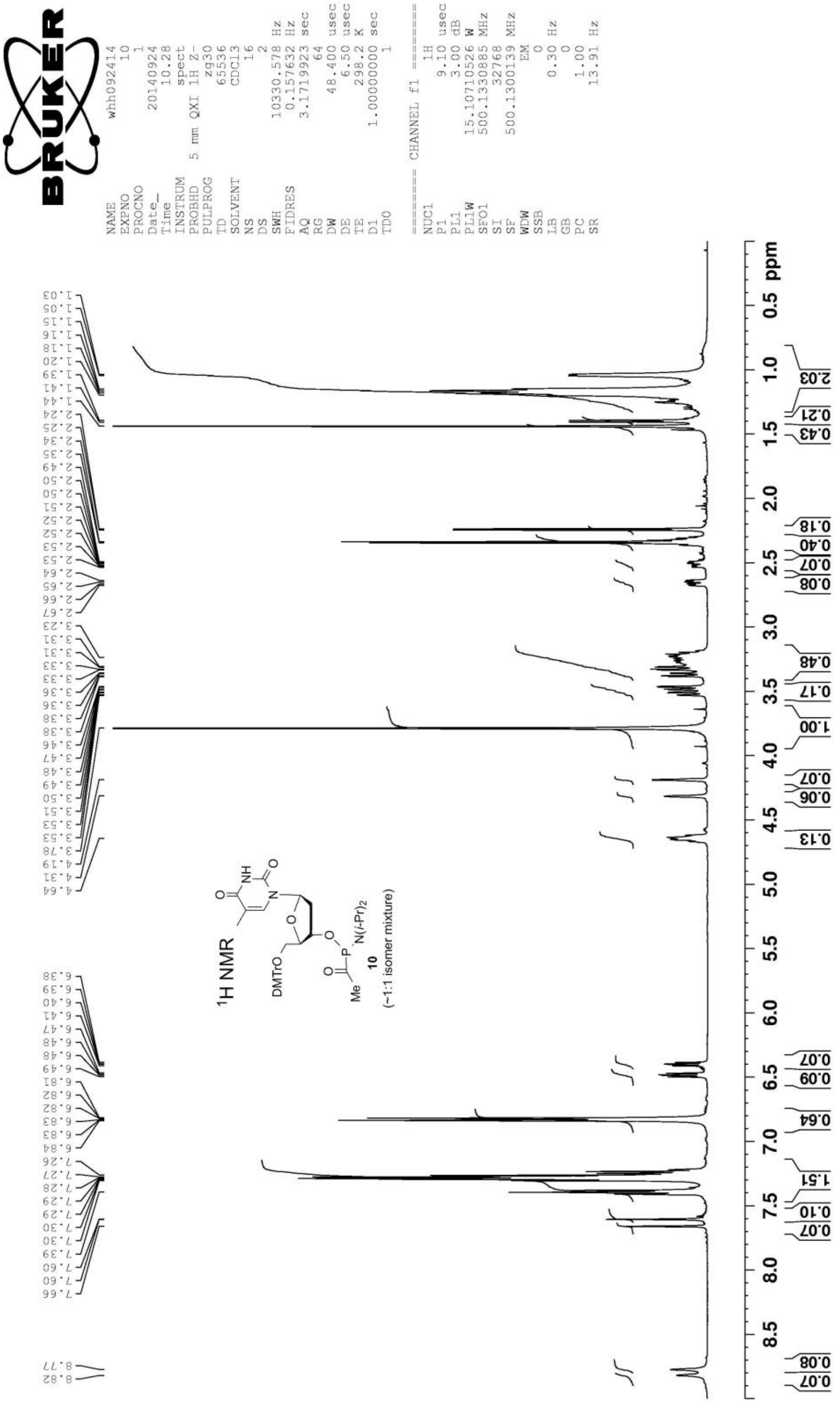

BRUKER
NAME whh092414
EXPNO 10
PROCNO 1
Date_ 20140924
Time 10.28
INSTRUM spect
PROBHD 5 mm QXI 1H Z-
PULPROG zg30
TD 65536
SOLVENT CDCl3
NS 16
DS 2
SWH 10330.578 Hz
FIDRES 0.157632 Hz
AQ 3.1719923 sec
RG 64
DW 48.400 usec
DE 6.50 usec
TE 298.2 K
D1 1.00000000 sec
TD0 1
======== CHANNEL f1 ========
NUC1 1H
P1 9.10 usec
PL1 3.00 dB
PL1W 15.10710526 W
SFO1 500.1330885 MHz
SI 32768
SF 500.1300139 MHz
WDW EM
SSB 0
LB 0.30 Hz
GB 0
PC 1.00
SR 13.91 Hz
1H NMR
DMTrO
Me
N(i-Pr)2
10
(~1:1 isomer mixture)
ppm

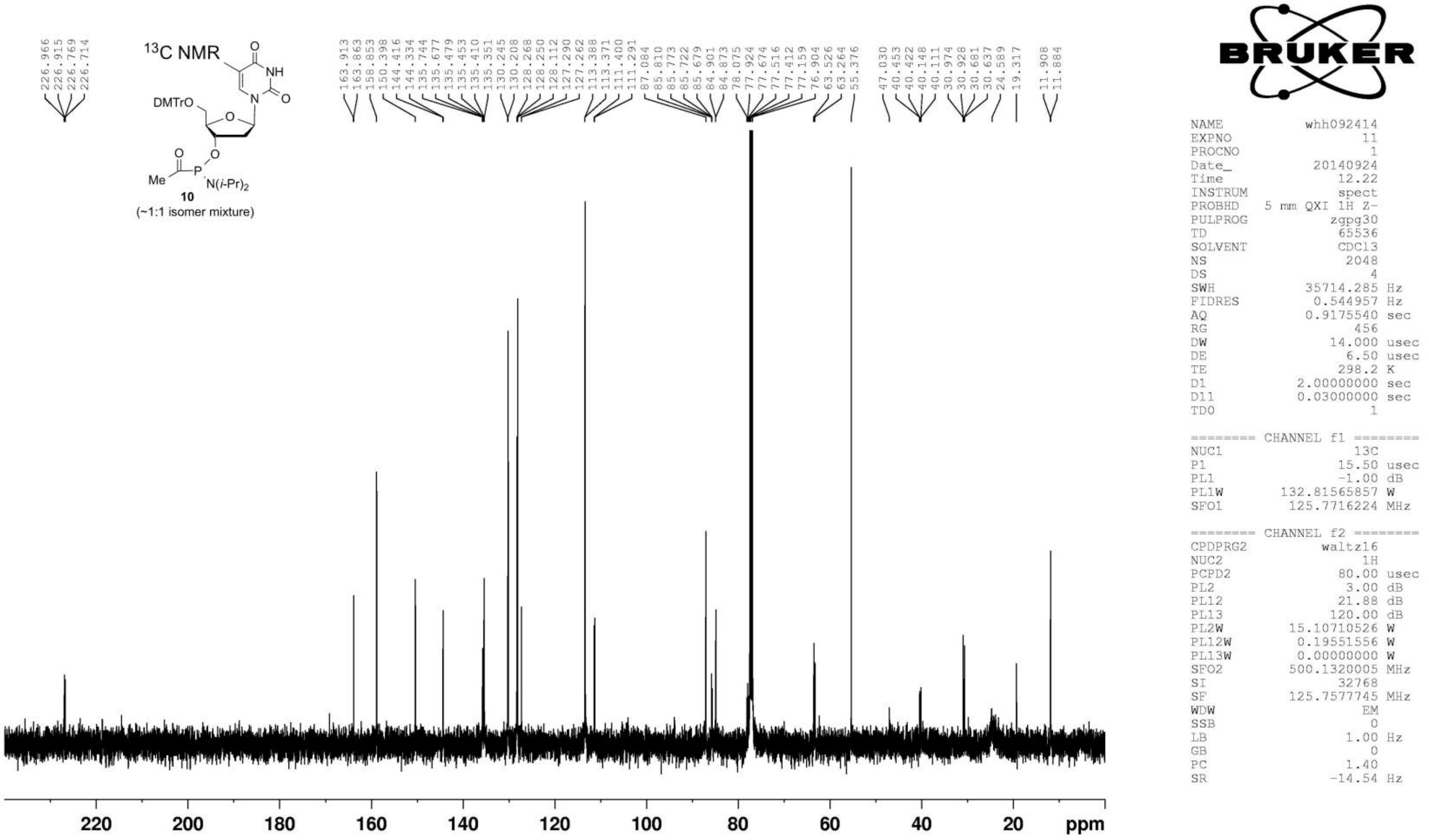

13C NMR
DMTrO
NH
O
N
Me
P
N(i-Pr)2
10
(~1:1 isomer mixture)
226.966 226.915 226.769 226.714
163.913 163.863 158.853 150.398 144.416 144.334 135.744 135.677 135.479 135.453 135.410 135.351 130.245 130.208 128.268 128.250 128.112 127.290 127.262 113.388 113.371 111.400 111.291 87.084 85.810 85.773 85.722 85.679 84.901 84.873 78.075 77.924 77.674 77.516 77.412 77.159 76.904 63.526 63.264 55.376 47.030 40.453 40.422 40.148 40.111 30.974 30.928 30.681 30.637 24.589 19.317 11.908 11.884
220 200 180 160 140 120 100 80 60 40 20 ppm
BRUKER
NAME whh092414
EXPNO 11
PROCNO 1
Date_ 20140924
Time 12.22
INSTRUM spect
PROBHD 5 mm QXI 1H Z-
PULPROG zgpg30
TD 65536
SOLVENT CDCl3
NS 2048
DS 4
SWH 35714.285 Hz
FIDRES 0.544957 Hz
AQ 0.9175540 sec
RG 456
DW 14.000 usec
DE 6.50 usec
TE 298.2 K
D1 2.00000000 sec
D11 0.03000000 sec
TD0 1
======== CHANNEL f1 ========
NUC1 13C
P1 15.50 usec
PL1 -1.00 dB
PL1W 132.81565857 W
SFO1 125.7716224 MHz
======== CHANNEL f2 ========
CPDPRG2 waltz16
NUC2 1H
PCPD2 80.00 usec
PL2 3.00 dB
PL12 21.88 dB
PL13 120.00 dB
PL2W 15.10710526 W
PL12W 0.19551556 W
PL13W 0.00000000 W
SFO2 500.1320005 MHz
SI 32768
SF 125.7577745 MHz
WDW EM
SSB 0
LB 1.00 Hz
GB 0
PC 1.40
SR -14.54 Hz

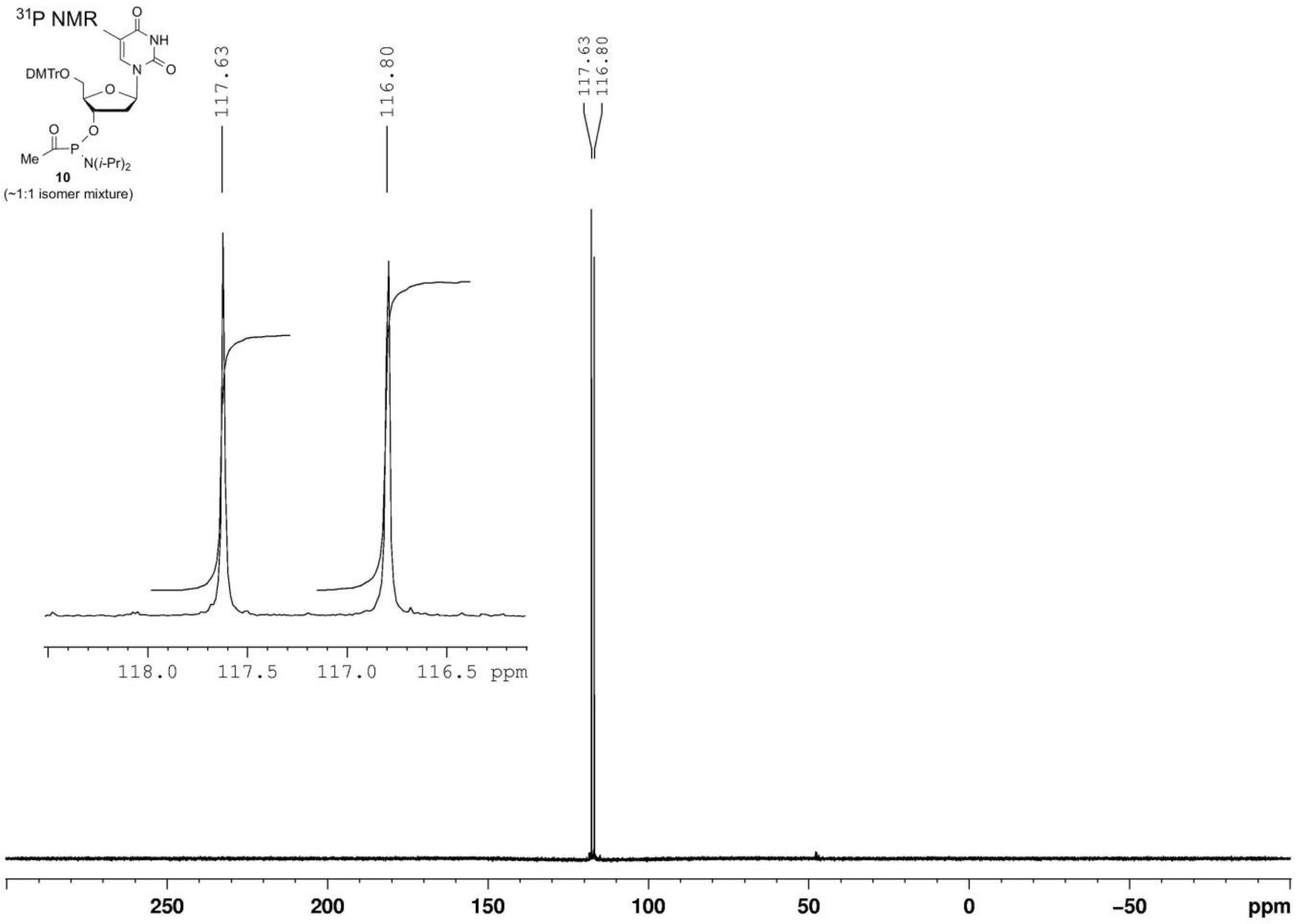


```
Current Data Parameters
NAME          whh040214
EXPNO                11
PROCNO                1

F2 - Acquisition Parameters
Date_          20140402
Time              21.59
INSTRUM           spect
PROBHD   5 mm QNP  1H/1
PULPROG          zgpg30
TD                65536
SOLVENT           CDCl3
NS                  512
DS                    4
SWH           64935.066 Hz
FIDRES         0.990830 Hz
AQ            0.5046772 sec
RG              20642.5
DW                7.700 usec
DE                 6.00 usec
TE                299.2 K
D1           2.00000000 sec
d11          0.03000000 sec
DELTA        1.89999998 sec
TD0                   1

======== CHANNEL f1 ========
NUC1                31P
P1                 4.65 usec
PL1               -6.00 dB
SFO1        161.9917906 MHz

======== CHANNEL f2 ========
CPDPRG2         waltz16
NUC2                 1H
PCPD2            100.00 usec
PL2               -6.00 dB
PL12              13.91 dB
PL13             120.00 dB
SFO2        400.1316005 MHz

F2 - Processing parameters
SI                32768
SF          161.9755358 MHz
WDW                  EM
SSB                   0
LB                 1.00 Hz
GB                    0
PC                 1.40
SR               -57.24 Hz
```

COSY90

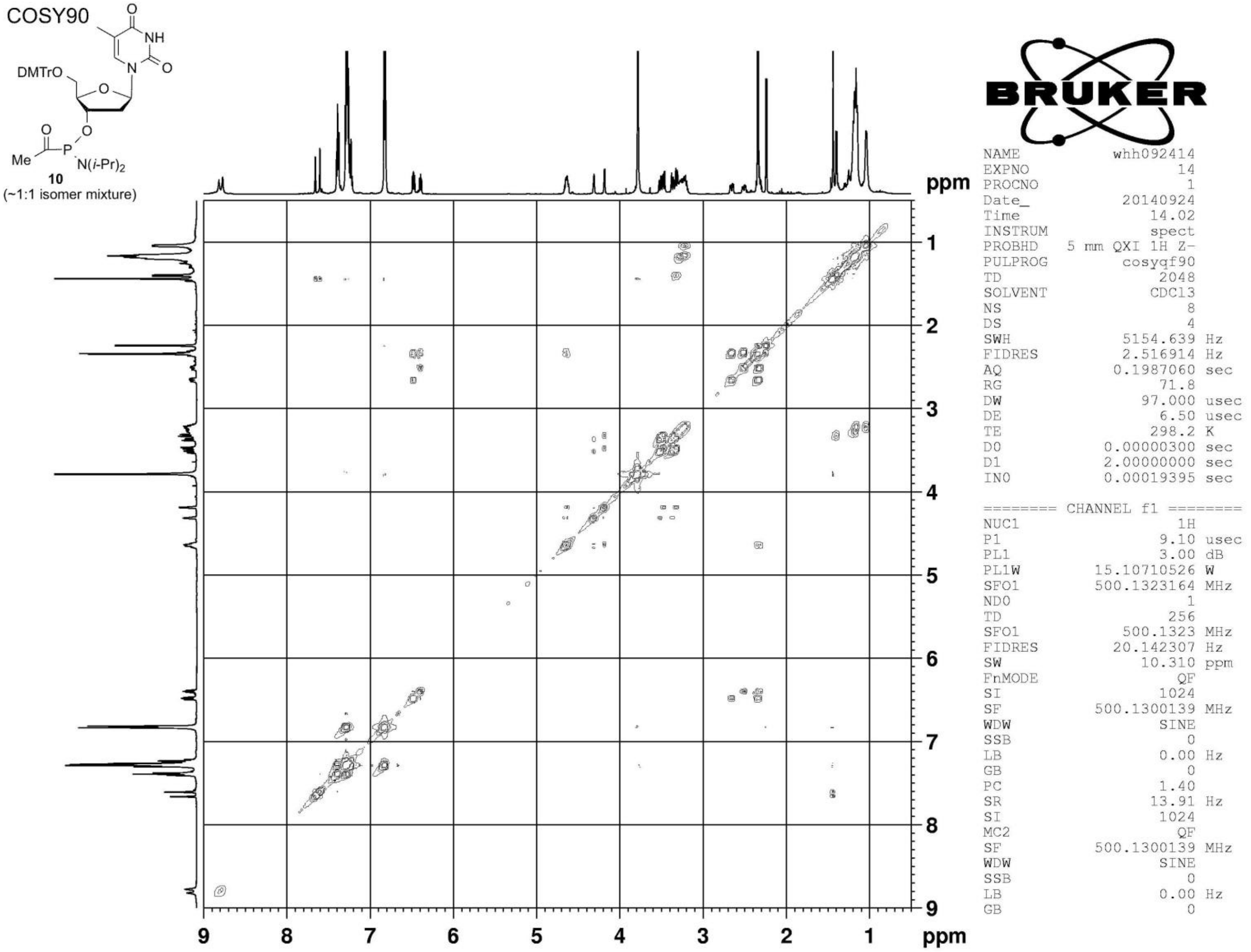

10
(~1:1 isomer mixture)

HSQC

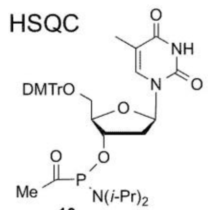

10

(~1:1 isomer mixture)

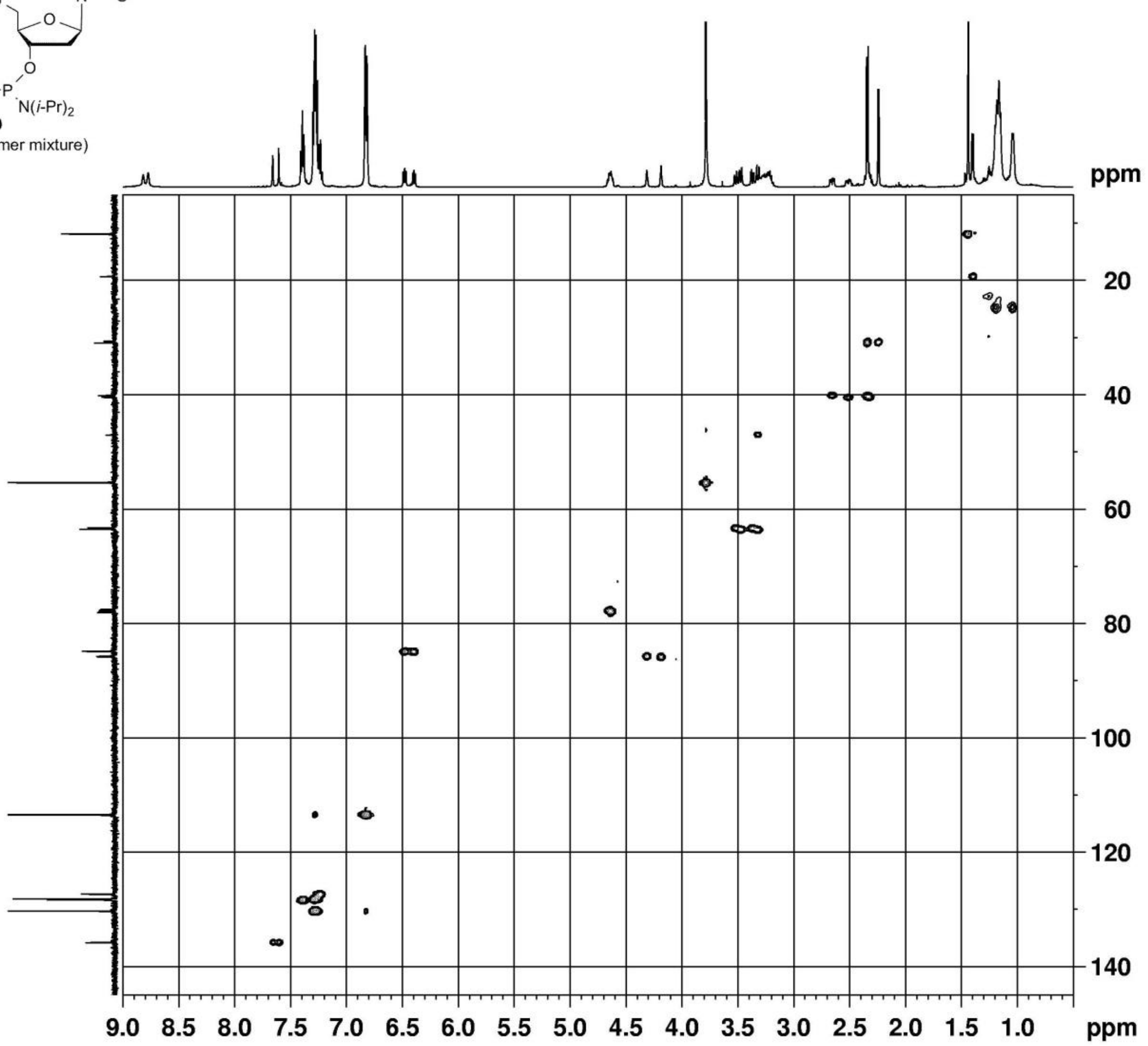


```
NAME          wm1092414
EXPNO                13
PROCNO                1
Date_          20140924
Time              13.35
INSTRUM           spect
PROBHD   5 mm QXI 1H Z-
PULPROG     hsqcetgpsi2
TD                 1024
SOLVENT           CDCl3
NS                    2
DS                   16
SWH            6666.667 Hz
FIDRES         6.510417 Hz
AQ            0.0768500 sec
RG                 2050
DW               75.000 usec
DE                 6.50 usec
TE                298.4 K
CNST2       145.0000000
D0           0.00000300 sec
D1           1.50000000 sec
D4           0.00172414 sec
D11          0.03000000 sec
D13          0.00000400 sec
D16          0.00020000 sec
D24          0.00086207 sec
IN0          0.00002210 sec
ZGOPTNS

======== CHANNEL f1 ========
NUC1                 1H
P1                 9.10 usec
P2                18.20 usec
P28             1000.00 usec
PL1                3.00 dB
PL1W        15.10710526 W
SFO1        500.1330069 MHz

======== CHANNEL f2 ========
CPDPRG2            garp
NUC2                13C
P3                15.50 usec
P4                31.00 usec
PCPD2             70.00 usec
PL2               -1.00 dB
PL12              12.10 dB
PL2W       132.81565857 W
PL12W        6.50502920 W
SFO2        125.7672177 MHz

====== GRADIENT CHANNEL =====
GPNAM1         SINE.100
GPNAM2         SINE.100
GPNAM3         SINE.100
GPNAM4         SINE.100
GPZ1              80.00 %
GPZ2              20.10 %
GPZ3              11.00 %
GPZ4              -5.00 %
P16             1000.00 usec
P19              600.00 usec
ND0                   2
TD                  256
SFO1           125.7672 MHz
FIDRES        88.430077 Hz
SW              180.000 ppm
FnMODE     Echo-Antiecho
SI                 1024
SF          500.1300139 MHz
WDW               QSINE
SSB                   2
LB                 0.00 Hz
GB                    0
PC                 1.40
SR                13.91 Hz
SI                 1024
MC2        echo-antiecho
SF          125.7577745 MHz
WDW               QSINE
SSB                   2
LB                 0.00 Hz
GB                    0
```

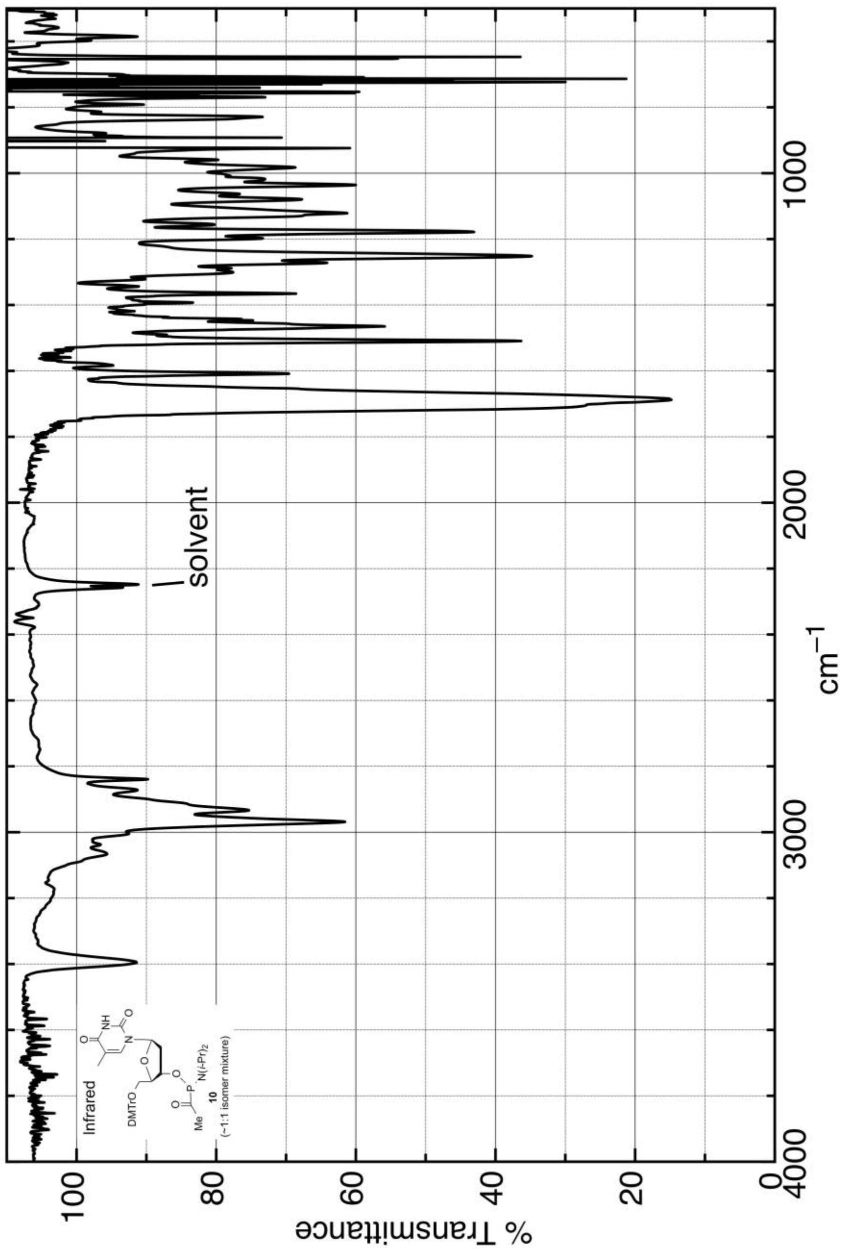
Infrared
DMTrO
NH
N(i-Pr)2
Me
10
(~1:1 isomer mixture)
solvent
% Transmittance
cm−1
4000
3000
2000
1000
0
20
40
60
80
100

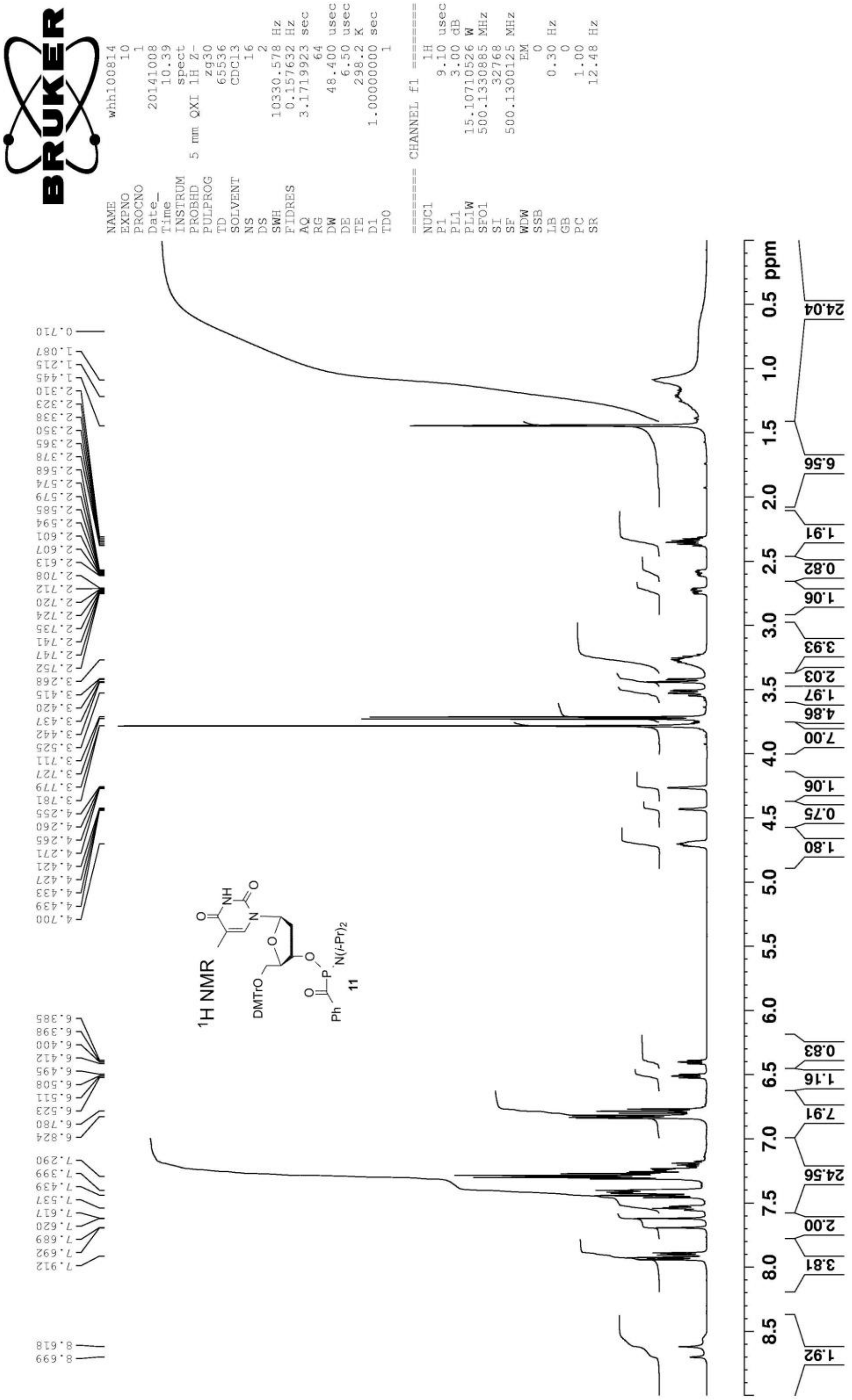
1H NMR
DMTrO
NH
O
N
P
Ph
N(i-Pr)2
11
NAME whh100814
EXPNO 10
PROCNO 1
Date_ 20141008
Time 10.39
INSTRUM spect
PROBHD 5 mm QXI 1H Z-
PULPROG zg30
TD 65536
SOLVENT CDCl3
NS 16
DS 2
SWH 10330.578 Hz
FIDRES 0.157632 Hz
AQ 3.1719923 sec
RG 64
DW 48.400 usec
DE 6.50 usec
TE 298.2 K
D1 1.00000000 sec
TD0 1
======== CHANNEL f1 ========
NUC1 1H
P1 9.10 usec
PL1 3.00 dB
PL1W 15.10710526 W
SFO1 500.1330885 MHz
SI 32768
SF 500.1300125 MHz
WDW EM
SSB 0
LB 0.30 Hz
GB 0
PC 1.00
SR 12.48 Hz
0.710
1.087
1.215
1.445
2.310
2.323
2.338
2.350
2.365
2.378
2.568
2.574
2.579
2.585
2.594
2.601
2.607
2.613
2.708
2.712
2.720
2.724
2.735
2.741
2.747
2.752
3.268
3.415
3.420
3.437
3.442
3.525
3.711
3.727
3.779
3.781
4.255
4.260
4.265
4.271
4.421
4.427
4.433
4.439
4.700
6.385
6.398
6.400
6.412
6.495
6.508
6.511
6.523
6.780
6.824
7.290
7.399
7.439
7.537
7.617
7.620
7.689
7.692
7.912
8.618
8.699
24.04
6.56
1.91
0.82
1.06
3.93
2.03
1.97
4.86
7.00
1.06
0.75
1.80
0.83
1.16
7.91
24.56
2.00
3.81
1.92
0.5 ppm
1.0
1.5
2.0
2.5
3.0
3.5
4.0
4.5
5.0
5.5
6.0
6.5
7.0
7.5
8.0
8.5

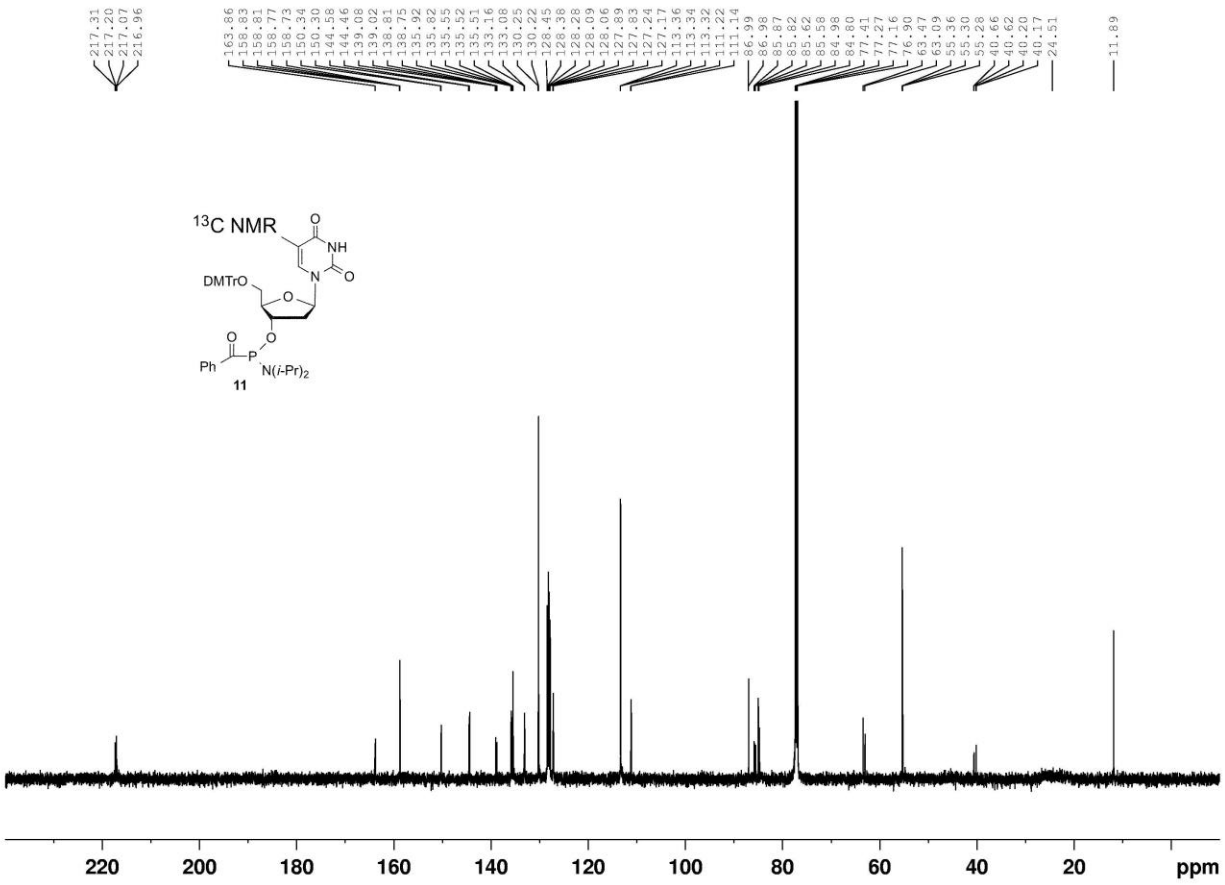


```
NAME           whh100814
EXPNO                 11
PROCNO                 1
Date_           20141008
Time               14.10
INSTRUM            spect
PROBHD   5 mm QXI 1H Z-
PULPROG          zgpg30
TD                 65536
SOLVENT            CDCl3
NS                  4096
DS                     4
SWH            35714.285 Hz
FIDRES          0.544957 Hz
AQ             0.9175540 sec
RG                   456
DW                14.000 usec
DE                  6.50 usec
TE                 298.2 K
D1            2.00000000 sec
D11           0.03000000 sec
TD0                    1

======== CHANNEL f1 ========
NUC1                 13C
P1                 15.50 usec
PL1                -1.00 dB
PL1W        132.81565857 W
SFO1         125.7716224 MHz

======== CHANNEL f2 ========
CPDPRG2          waltz16
NUC2                  1H
PCPD2              80.00 usec
PL2                 3.00 dB
PL12               21.88 dB
PL13              120.00 dB
PL2W         15.10710526 W
PL12W         0.19551556 W
PL13W         0.00000000 W
SFO2         500.1320005 MHz
SI                 32768
SF           125.7577745 MHz
WDW                   EM
SSB                    0
LB                  1.00 Hz
GB                     0
PC                  1.40
SR                -14.52 Hz
```

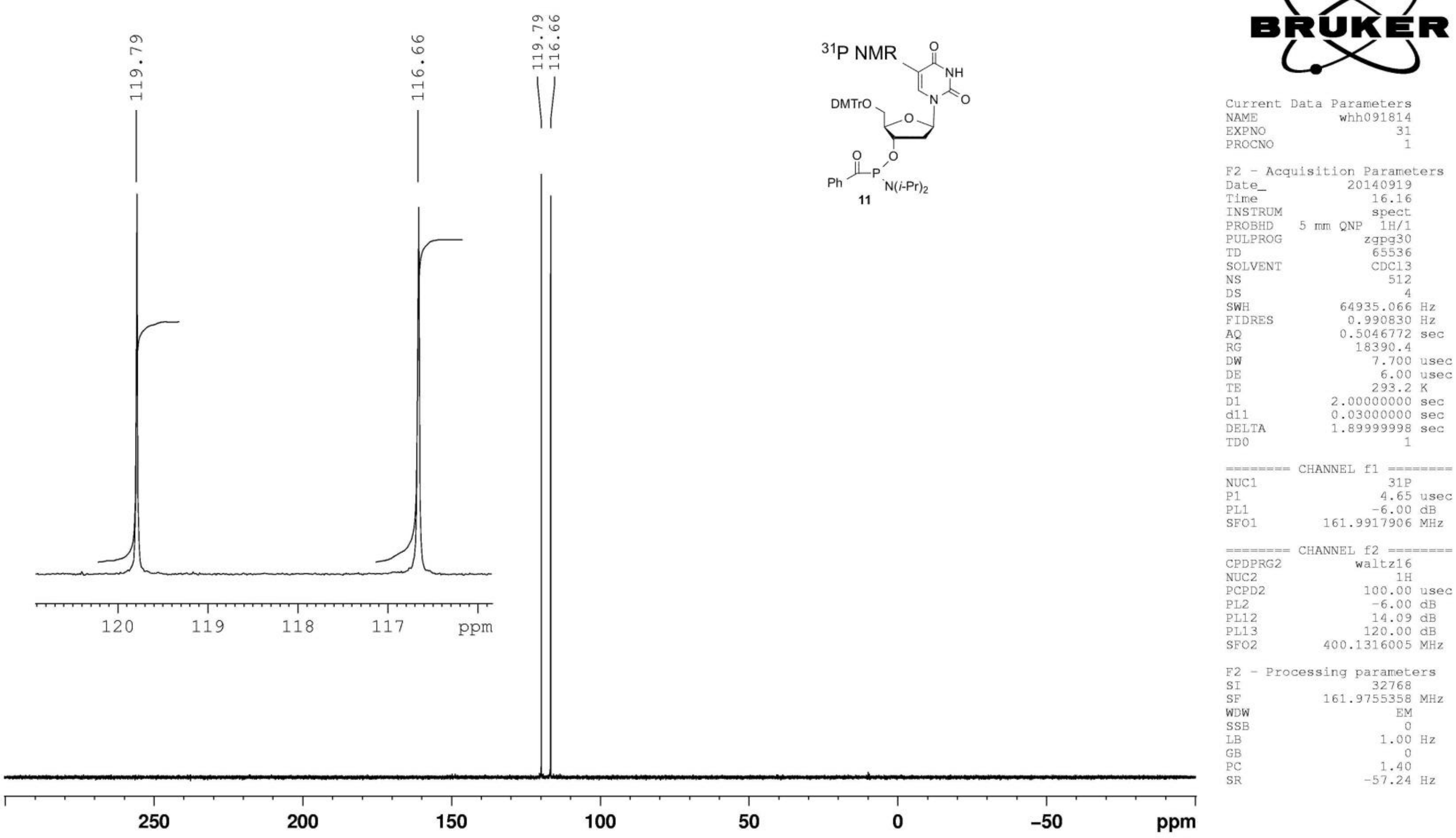

31P NMR
DMTrO
Ph
N(i-Pr)2
11
119.79
116.66
120
119
118
117
ppm
250
200
150
100
50
0
–50
ppm
Current Data Parameters
NAME whh091814
EXPNO 31
PROCNO 1
F2 - Acquisition Parameters
Date_ 20140919
Time 16.16
INSTRUM spect
PROBHD 5 mm QNP 1H/1
PULPROG zgpg30
TD 65536
SOLVENT CDCl3
NS 512
DS 4
SWH 64935.066 Hz
FIDRES 0.990830 Hz
AQ 0.5046772 sec
RG 18390.4
DW 7.700 usec
DE 6.00 usec
TE 293.2 K
D1 2.00000000 sec
d11 0.03000000 sec
DELTA 1.89999998 sec
TD0 1
======== CHANNEL f1 ========
NUC1 31P
P1 4.65 usec
PL1 -6.00 dB
SFO1 161.9917906 MHz
======== CHANNEL f2 ========
CPDPRG2 waltz16
NUC2 1H
PCPD2 100.00 usec
PL2 -6.00 dB
PL12 14.09 dB
PL13 120.00 dB
SFO2 400.1316005 MHz
F2 - Processing parameters
SI 32768
SF 161.9755358 MHz
WDW EM
SSB 0
LB 1.00 Hz
GB 0
PC 1.40
SR -57.24 Hz

COSY90

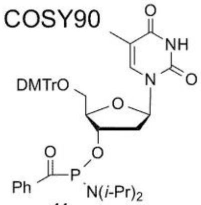

11

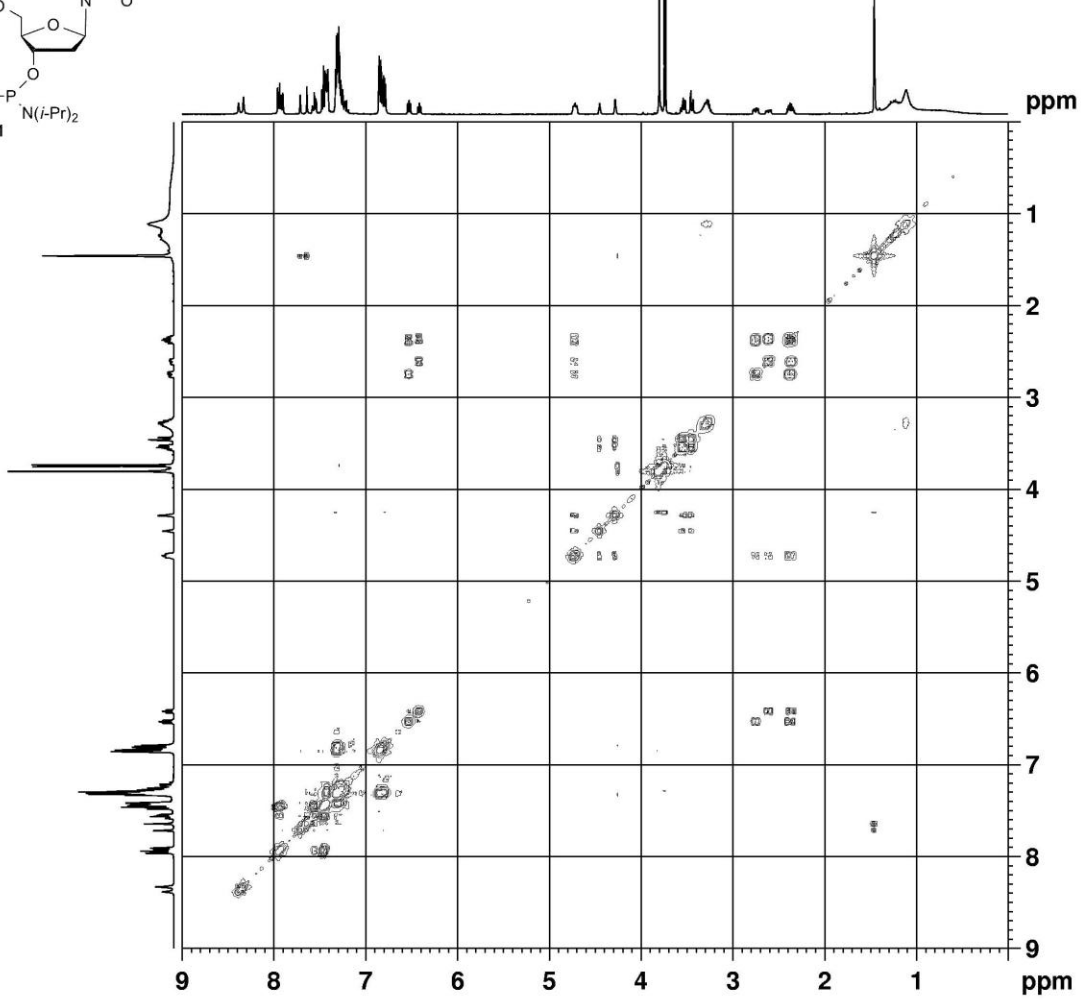

```
Current Data Parameters
NAME            whh091814
EXPNO                  65
PROCNO                  1

F2 - Acquisition Parameters
Date_            20140919
Time                21.08
INSTRUM             spect
PROBHD   5 mm QNP  1H/1
PULPROG          cosygf90
TD                   2048
SOLVENT             CDCl3
NS                      8
DS                      4
SWH              3810.976 Hz
FIDRES           1.860828 Hz
AQ              0.2687476 sec
RG                  203.2
DW                131.200 usec
DE                   6.00 usec
TE                  293.2 K
d0             0.00000300 sec
D1             1.98074806 sec
IN0            0.00026240 sec

======== CHANNEL f1 ========
NUC1                   1H
P1                   9.90 usec
PL1                 -6.00 dB
SFO1          400.1317018 MHz

F1 - Acquisition parameters
ND0                     1
TD                    256
SFO1             400.1317 MHz
FIDRES          14.886623 Hz
SW                  9.524 ppm
FnMODE                 QF

F2 - Processing parameters
SI                   1024
SF            400.1300000 MHz
WDW                  SINE
SSB                     0
LB                   0.00 Hz
GB                      0
PC                   1.40
SR                   0.00 Hz

F1 - Processing parameters
SI                   1024
MC2                    QF
SF            400.1300000 MHz
WDW                  SINE
SSB                     0
LB                   0.00 Hz
GB                      0
```

HETCOR

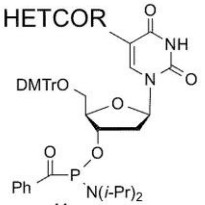

11

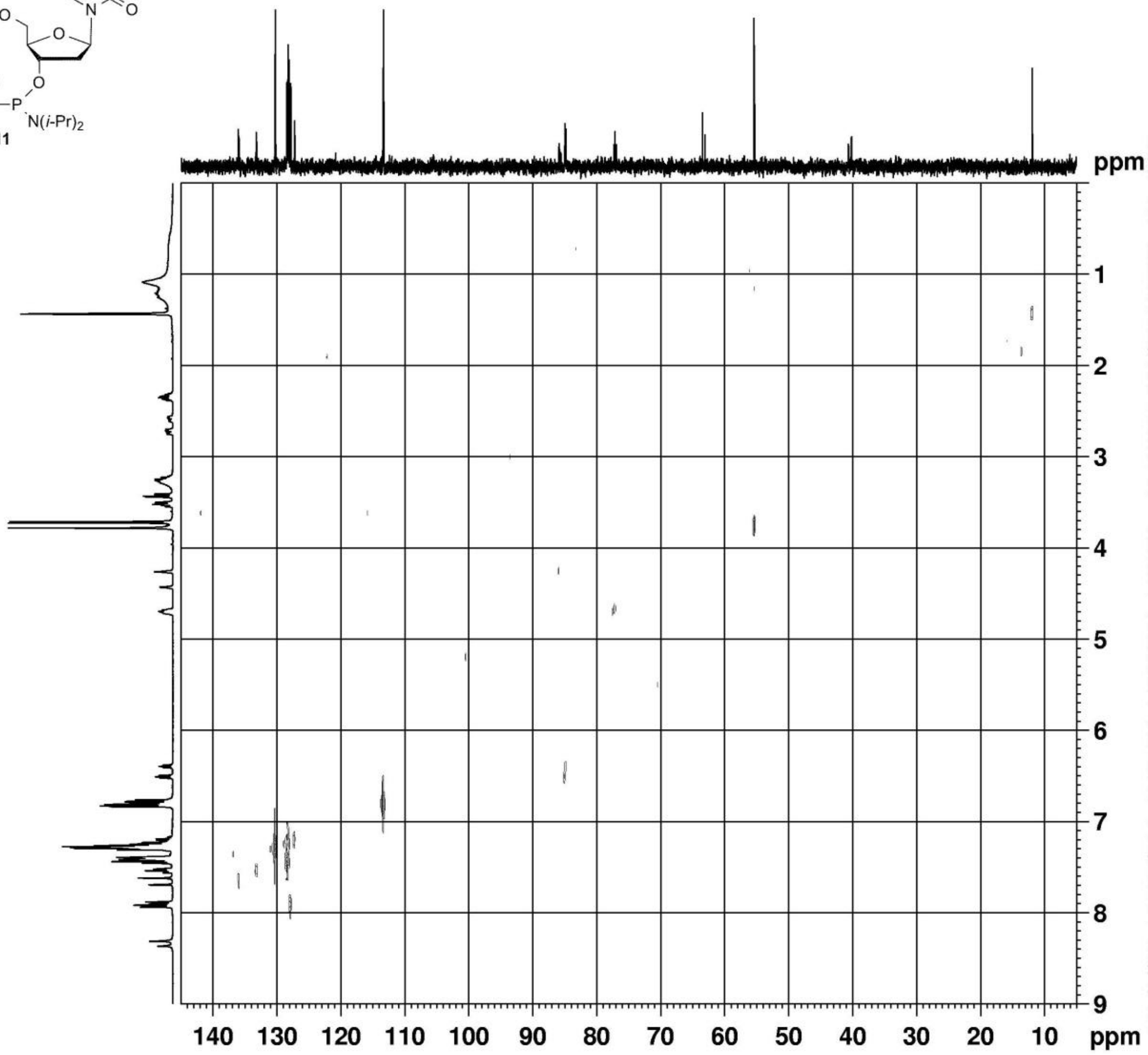


```
Current Data Parameters
NAME            whh091814
EXPNO                  64
PROCNO                  1

F2 - Acquisition Parameters
Date_            20140919
Time                20.30
INSTRUM             spect
PROBHD   5 mm QNP  1H/1
PULPROG            hxcoqf
TD                   4096
SOLVENT             CDCl3
NS                      8
DS                      4
SWH             18518.518 Hz
FIDRES           4.521122 Hz
AQ              0.1106420 sec
RG                  16384
DW                 27.000 usec
DE                   6.00 usec
TE                  293.2 K
CNST2         145.0000000
CNST11          3.0000000
d0             0.00000300 sec
D1             2.01474190 sec
d2             0.00344828 sec
d3             0.00229885 sec
d11            0.03000000 sec
d12            0.00002000 sec
IN0            0.00013120 sec

======== CHANNEL f1 ========
NUC1                  13C
P1                   7.10 usec
p2                  14.20 usec
PL1                 -6.00 dB
SFO1          100.6215196 MHz

======== CHANNEL f2 ========
CPDPRG2           waltz16
NUC2                   1H
P3                   9.90 usec
PCPD2              100.00 usec
PL2                 -6.00 dB
PL12                14.09 dB
SFO2          400.1317018 MHz

F1 - Acquisition parameters
ND0                     2
TD                    128
SFO1             400.1317 MHz
FIDRES          29.773247 Hz
SW                  9.524 ppm
FnMODE                 QF

F2 - Processing parameters
SI                   2048
SF            100.6127558 MHz
WDW                 QSINE
SSB                     2
LB                   0.00 Hz
GB                      0
PC                   1.40
SR                 -13.23 Hz

F1 - Processing parameters
SI                   1024
MC2                    QF
SF            400.1300095 MHz
WDW                 QSINE
SSB                     2
LB                   0.00 Hz
GB                      0
```

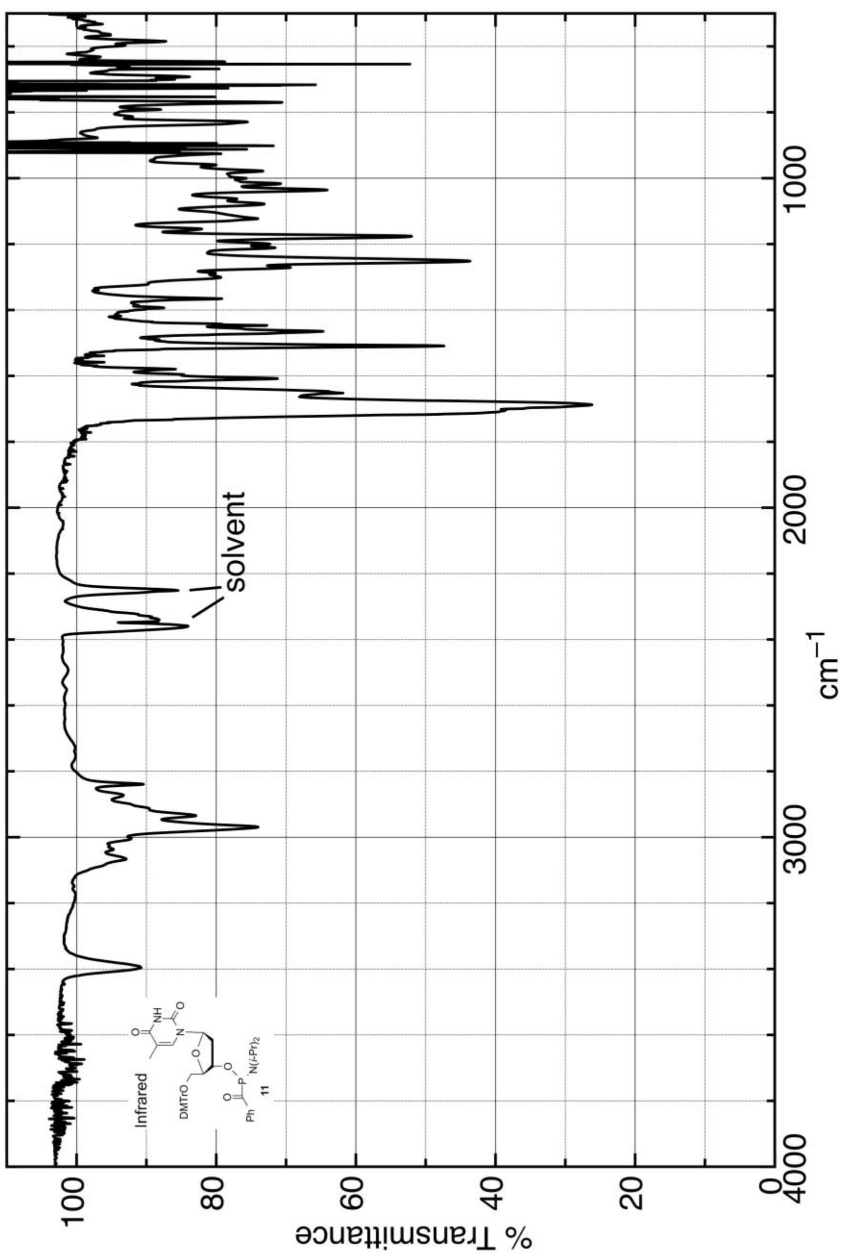
Infrared
DMTrO
Ph
N(i-Pr)2
11
solvent
% Transmittance
cm−1

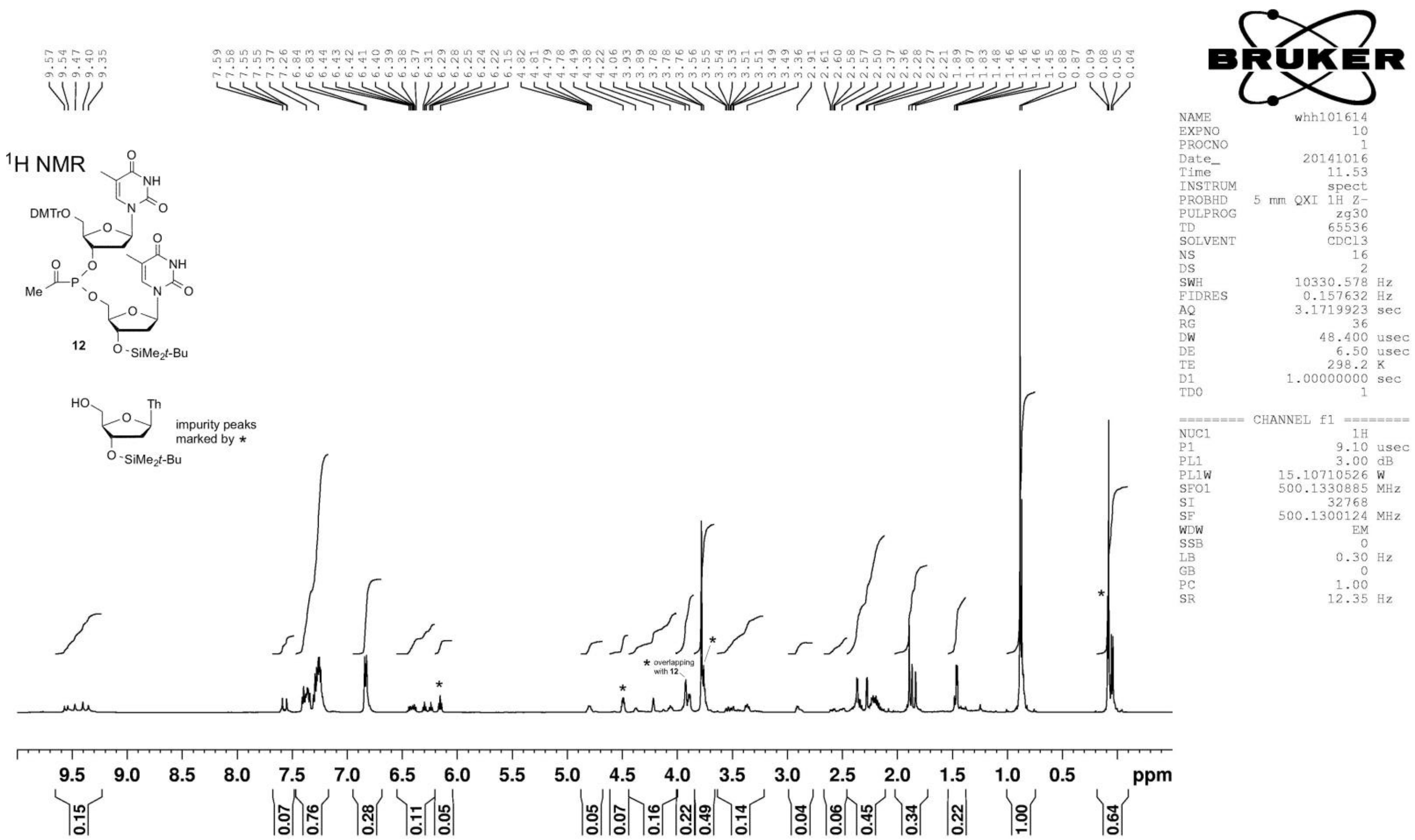
1H NMR
DMTrO
Me
12
O-SiMe2t-Bu
HO
Th
impurity peaks marked by *
* overlapping with 12
9.57 9.54 9.47 9.40 9.35
7.59 7.58 7.55 7.55 7.37 7.26 6.84 6.83 6.44 6.43 6.42 6.41 6.40 6.39 6.38 6.37 6.31 6.29 6.28 6.25 6.24 6.22 6.15
4.82 4.81 4.79 4.78 4.49 4.38 4.22 4.06 3.93 3.89 3.78 3.78 3.76 3.56 3.55 3.54 3.53 3.51 3.51 3.49 3.49 3.36
2.91 2.61 2.60 2.58 2.57 2.50 2.37 2.36 2.28 2.27 2.21 1.89 1.87 1.83 1.48 1.46 1.46 1.46 1.45 1.45 0.88 0.87 0.09 0.08 0.05 0.04
0.15 0.07 0.76 0.28 0.11 0.05 0.05 0.07 0.16 0.22 0.49 0.14 0.04 0.06 0.45 0.34 0.22 1.00 0.64
9.5 9.0 8.5 8.0 7.5 7.0 6.5 6.0 5.5 5.0 4.5 4.0 3.5 3.0 2.5 2.0 1.5 1.0 0.5 ppm
BRUKER
NAME whh101614
EXPNO 10
PROCNO 1
Date_ 20141016
Time 11.53
INSTRUM spect
PROBHD 5 mm QXI 1H Z-
PULPROG zg30
TD 65536
SOLVENT CDCl3
NS 16
DS 2
SWH 10330.578 Hz
FIDRES 0.157632 Hz
AQ 3.1719923 sec
RG 36
DW 48.400 usec
DE 6.50 usec
TE 298.2 K
D1 1.00000000 sec
TD0 1
======== CHANNEL f1 ========
NUC1 1H
P1 9.10 usec
PL1 3.00 dB
PL1W 15.10710526 W
SFO1 500.1330885 MHz
SI 32768
SF 500.1300124 MHz
WDW EM
SSB 0
LB 0.30 Hz
GB 0
PC 1.00
SR 12.35 Hz

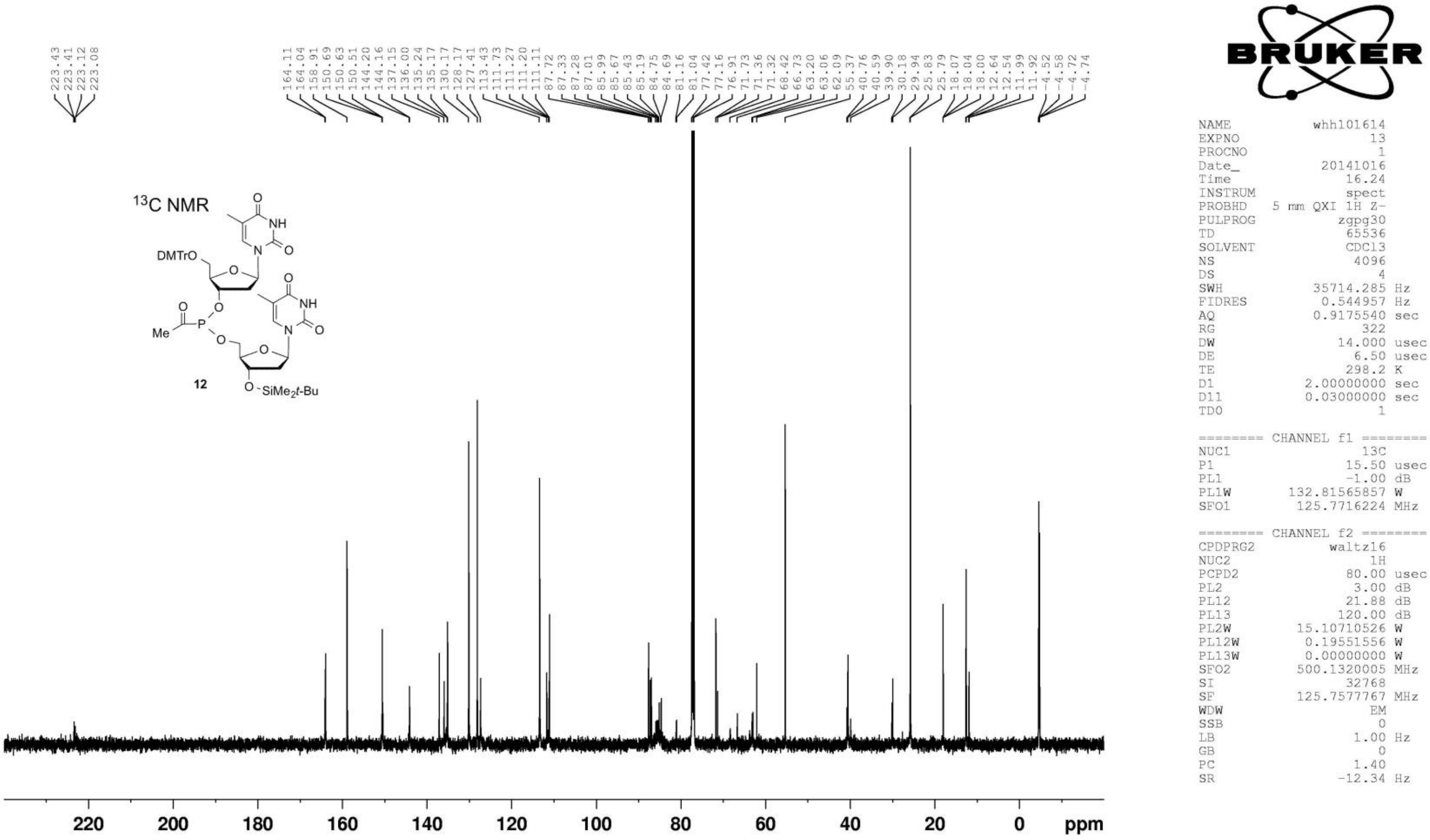

13C NMR
12
DMTrO
Me
O-SiMe2t-Bu
223.43 223.41 223.12 223.08
164.11 164.04 158.91 150.69 150.63 150.51 144.20 144.16 137.15 136.00 135.24 135.17 130.17 128.17 127.41 113.43 111.73 111.27 111.20 111.11 87.72 87.33 87.28 87.01 85.99 85.67 85.43 85.19 84.75 84.69 81.16 81.04 77.42 77.16 76.91 71.73 71.36 71.32 68.42 66.73 63.20 63.06 62.09 55.37 40.76 40.59 39.90 30.18 29.94 25.83 25.79 18.07 18.04 18.00 12.64 12.54 11.99 11.92 -4.52 -4.58 -4.72 -4.74
220 200 180 160 140 120 100 80 60 40 20 0 ppm
NAME whh101614
EXPNO 13
PROCNO 1
Date_ 20141016
Time 16.24
INSTRUM spect
PROBHD 5 mm QXI 1H Z-
PULPROG zgpg30
TD 65536
SOLVENT CDCl3
NS 4096
DS 4
SWH 35714.285 Hz
FIDRES 0.544957 Hz
AQ 0.9175540 sec
RG 322
DW 14.000 usec
DE 6.50 usec
TE 298.2 K
D1 2.00000000 sec
D11 0.03000000 sec
TD0 1
======== CHANNEL f1 ========
NUC1 13C
P1 15.50 usec
PL1 -1.00 dB
PL1W 132.81565857 W
SFO1 125.7716224 MHz
======== CHANNEL f2 ========
CPDPRG2 waltz16
NUC2 1H
PCPD2 80.00 usec
PL2 3.00 dB
PL12 21.88 dB
PL13 120.00 dB
PL2W 15.10710526 W
PL12W 0.19551556 W
PL13W 0.00000000 W
SFO2 500.1320005 MHz
SI 32768
SF 125.7577767 MHz
WDW EM
SSB 0
LB 1.00 Hz
GB 0
PC 1.40
SR -12.34 Hz

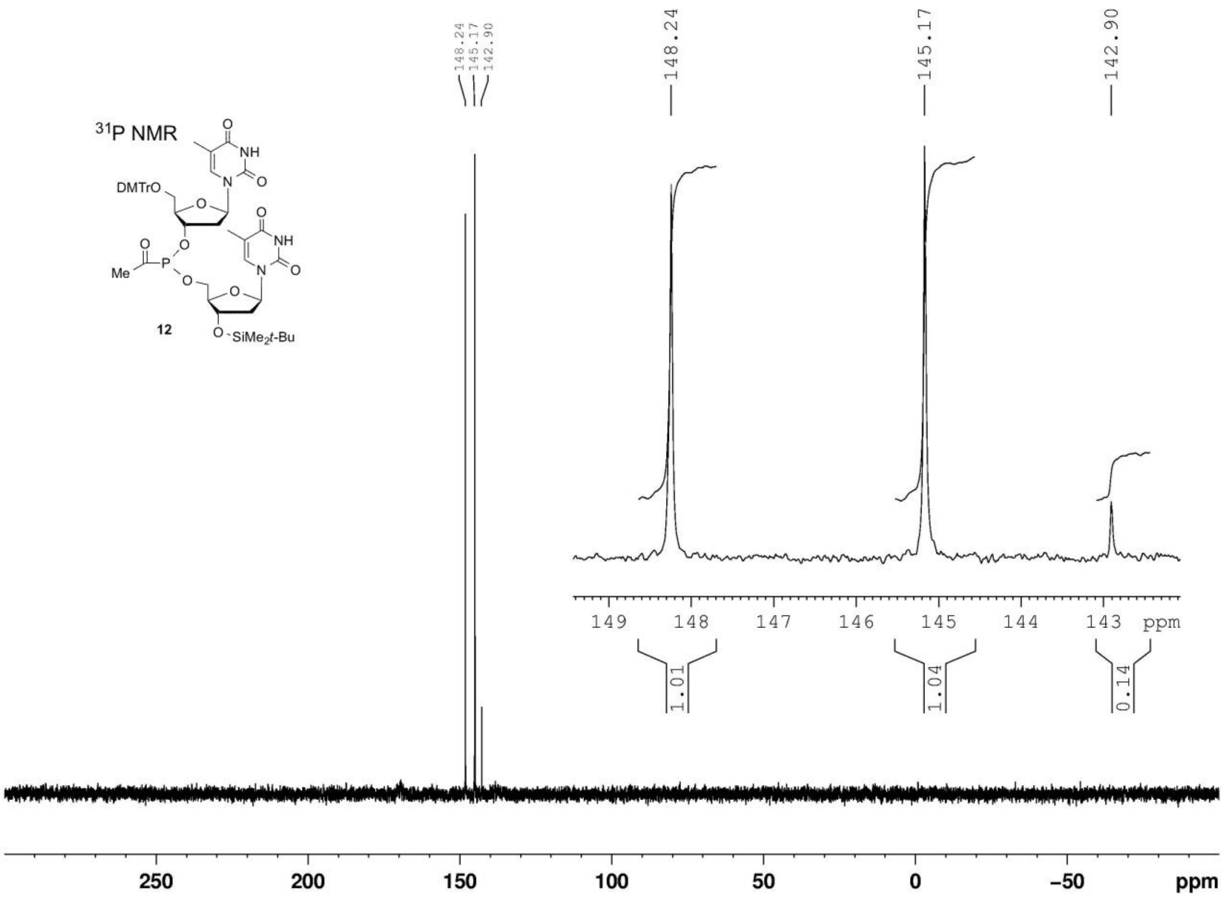

BRUKER

NAME	whh101614
EXPNO	15
PROCNO	1
Date_	20141016
Time	18.07
INSTRUM	spect
PROBHD	5 mm QXI 1H Z-
PULPROG	zgpg30
TD	65536
SOLVENT	CDCl3
NS	128
DS	4
SWH	81521.742 Hz
FIDRES	1.243923 Hz
AQ	0.4020041 sec
RG	2050
DW	6.133 usec
DE	6.50 usec
TE	298.2 K
D1	2.00000000 sec
D11	0.03000000 sec
TD0	1

======== CHANNEL f1 ========

NUC1	31P
P1	38.75 usec
PL1	0.00 dB
PL1W	90.17671967 W
SFO1	202.4765806 MHz

======== CHANNEL f2 ========

CPDPRG2	waltz16
NUC2	1H
PCPD2	80.00 usec
PL2	3.00 dB
PL12	21.88 dB
PL13	120.00 dB
PL2W	15.10710526 W
PL12W	0.19551556 W
PL13W	0.00000000 W
SFO2	500.1320005 MHz
SI	32768
SF	202.4563071 MHz
WDW	EM
SSB	0
LB	4.00 Hz
GB	0
PC	1.40
SR	-27.94 Hz

COSY90

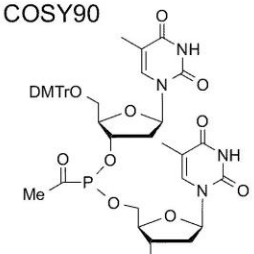

12

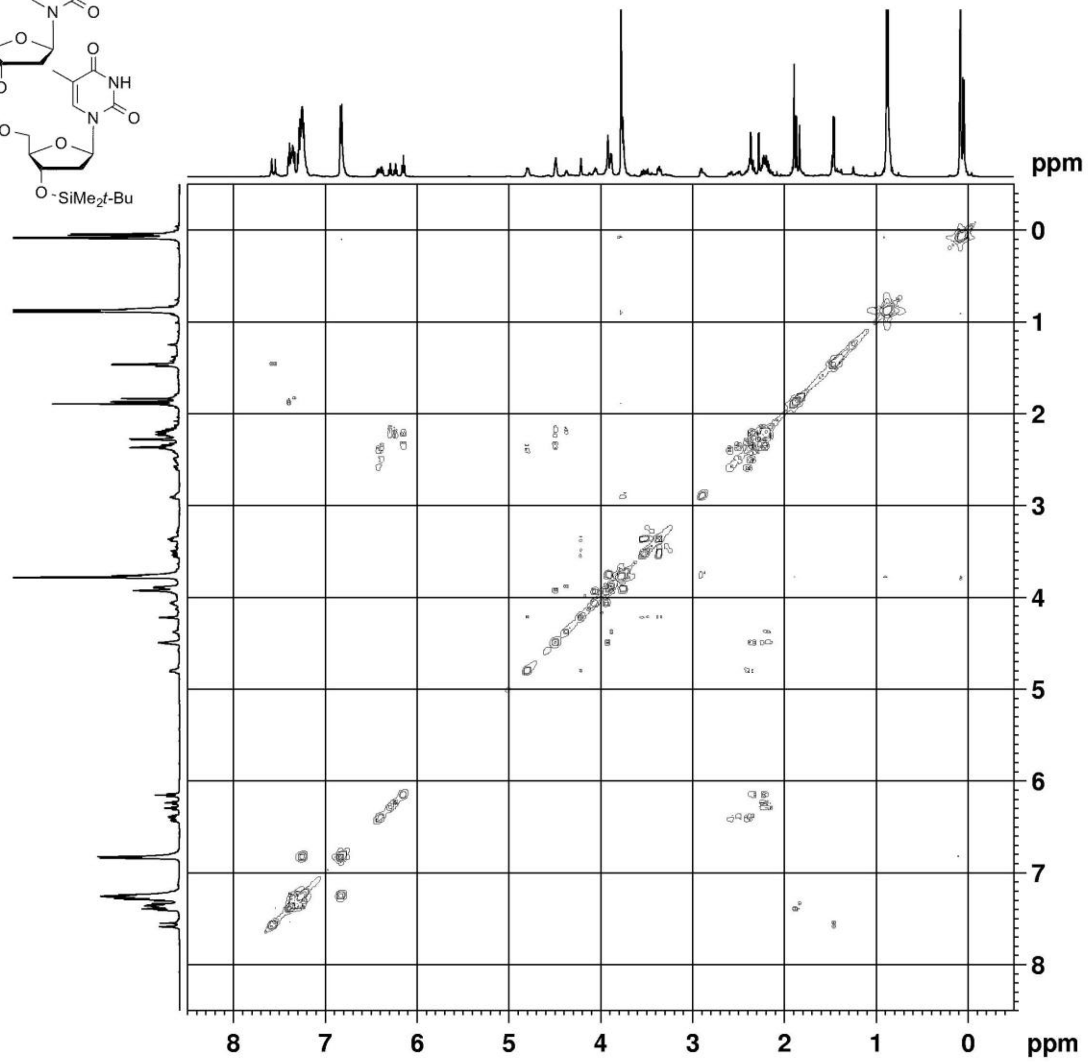

NAME	whh101614
EXPNO	14
PROCNO	1
Date_	20141016
Time	16.35
INSTRUM	spect
PROBHD	5 mm QXI 1H Z-
PULPROG	cosyqf90
TD	2048
SOLVENT	CDCl3
NS	8
DS	4
SWH	5154.639 Hz
FIDRES	2.516914 Hz
AQ	0.1987060 sec
RG	28.5
DW	97.000 usec
DE	6.50 usec
TE	298.2 K
D0	0.00000300 sec
D1	2.00000000 sec
IN0	0.00019395 sec
======== CHANNEL f1 ========	
NUC1	1H
P1	9.10 usec
PL1	3.00 dB
PL1W	15.10710526 W
SFO1	500.1323164 MHz
ND0	1
TD	256
SFO1	500.1323 MHz
FIDRES	20.142307 Hz
SW	10.310 ppm
FnMODE	QF
SI	1024
SF	500.1300124 MHz
WDW	SINE
SSB	0
LB	0.00 Hz
GB	0
PC	1.40
SR	12.35 Hz
SI	1024
MC2	QF
SF	500.1300124 MHz
WDW	SINE
SSB	0
LB	0.00 Hz
GB	0

HSQC

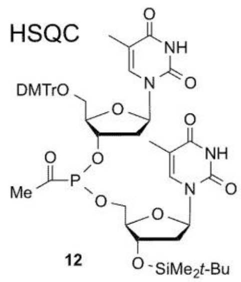
DMTrO
O
NH
N
Me
P
O-SiMe2t-Bu

12

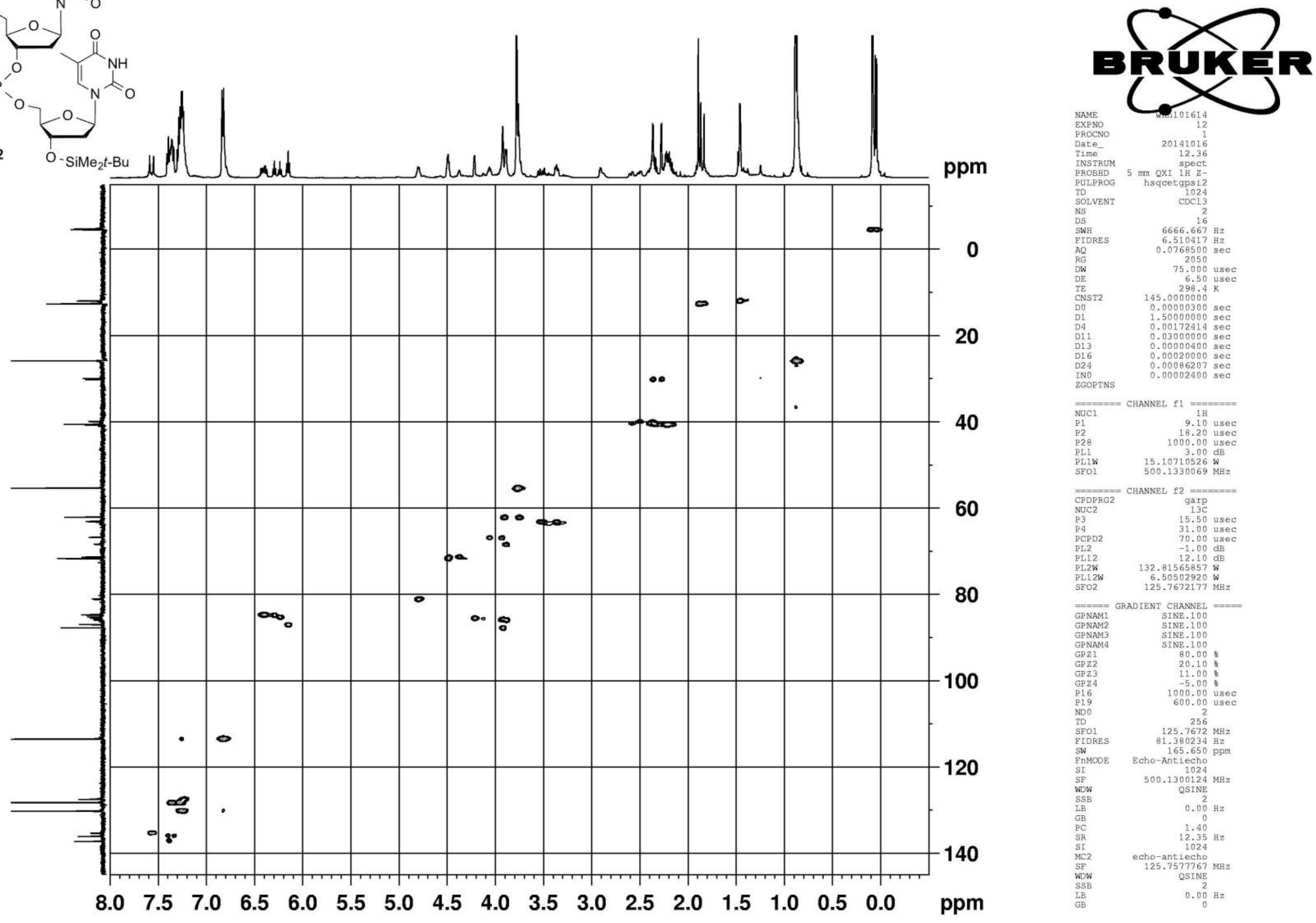
ppm
0
20
40
60
80
100
120
140
8.0 7.5 7.0 6.5 6.0 5.5 5.0 4.5 4.0 3.5 3.0 2.5 2.0 1.5 1.0 0.5 0.0
BRUKER
NAME wm101614
EXPNO 12
PROCNO 1
Date_ 20141016
Time 12.36
INSTRUM spect
PROBHD 5 mm QXI 1H Z-
PULPROG hsqcetgpsi2
TD 1024
SOLVENT CDCl3
NS 2
DS 16
SWH 6666.667 Hz
FIDRES 6.510417 Hz
AQ 0.0768500 sec
RG 2050
DW 75.000 usec
DE 6.50 usec
TE 298.4 K
CNST2 145.0000000
D0 0.00000300 sec
D1 1.50000000 sec
D4 0.00172414 sec
D11 0.03000000 sec
D13 0.00000400 sec
D16 0.00020000 sec
D24 0.00086207 sec
IN0 0.00002400 sec
ZGOPTNS
======== CHANNEL f1 ========
NUC1 1H
P1 9.10 usec
P2 18.20 usec
P28 1000.00 usec
PL1 3.00 dB
PL1W 15.10710526 W
SFO1 500.1330069 MHz
======== CHANNEL f2 ========
CPDPRG2 garp
NUC2 13C
P3 15.50 usec
P4 31.00 usec
PCPD2 70.00 usec
PL2 -1.00 dB
PL12 12.10 dB
PL2W 132.81565857 W
PL12W 6.50502920 W
SFO2 125.7672177 MHz
====== GRADIENT CHANNEL =====
GPNAM1 SINE.100
GPNAM2 SINE.100
GPNAM3 SINE.100
GPNAM4 SINE.100
GPZ1 80.00 %
GPZ2 20.10 %
GPZ3 11.00 %
GPZ4 -5.00 %
P16 1000.00 usec
P19 600.00 usec
ND0 2
TD 256
SFO1 125.7672 MHz
FIDRES 81.380234 Hz
SW 165.650 ppm
FnMODE Echo-Antiecho
SI 1024
SF 500.1300124 MHz
WDW QSINE
SSB 2
LB 0.00 Hz
GB 0
PC 1.40
SR 12.35 Hz
SI 1024
MC2 echo-antiecho
SF 125.7577767 MHz
WDW QSINE
SSB 2
LB 0.00 Hz
GB 0

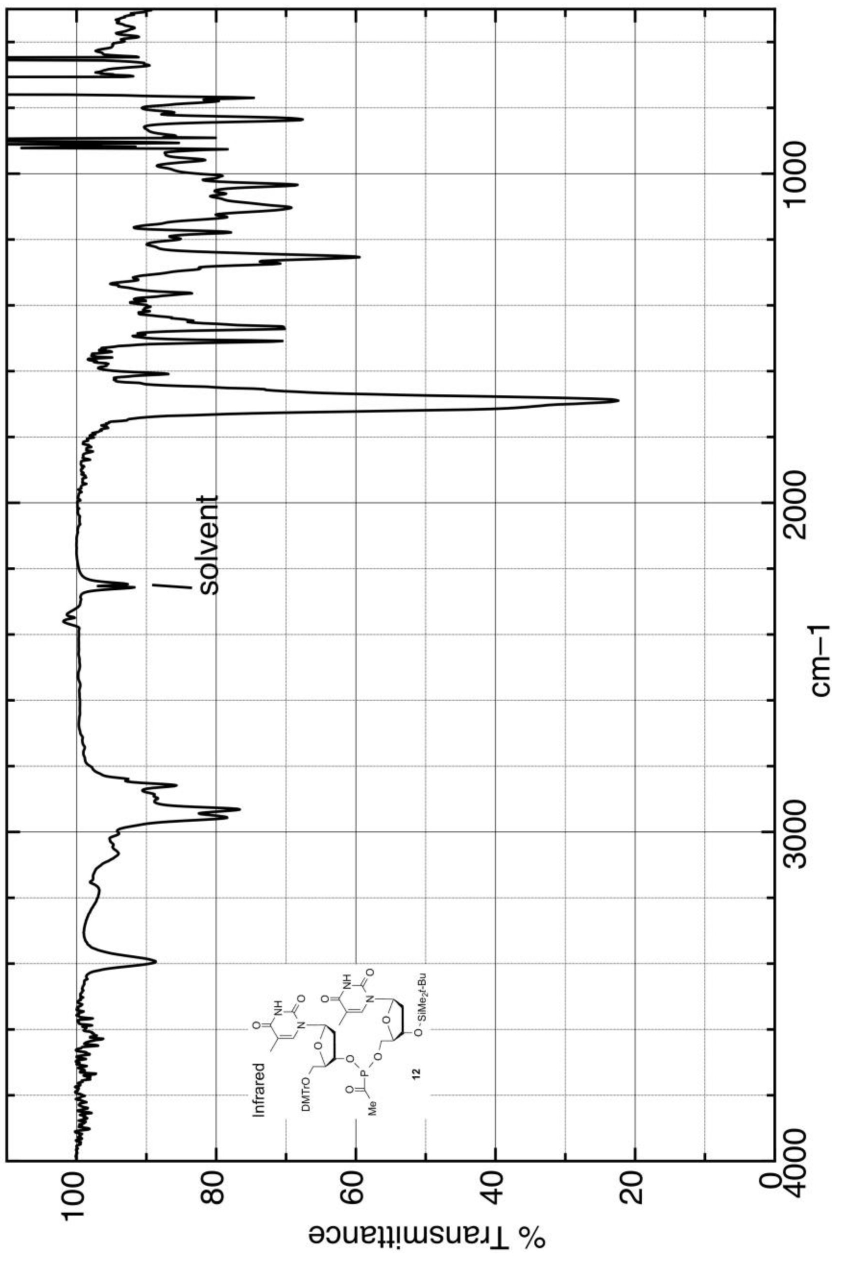

Infrared
DMTrO
Me
12
SiMe2t-Bu
solvent
% Transmittance
cm−1

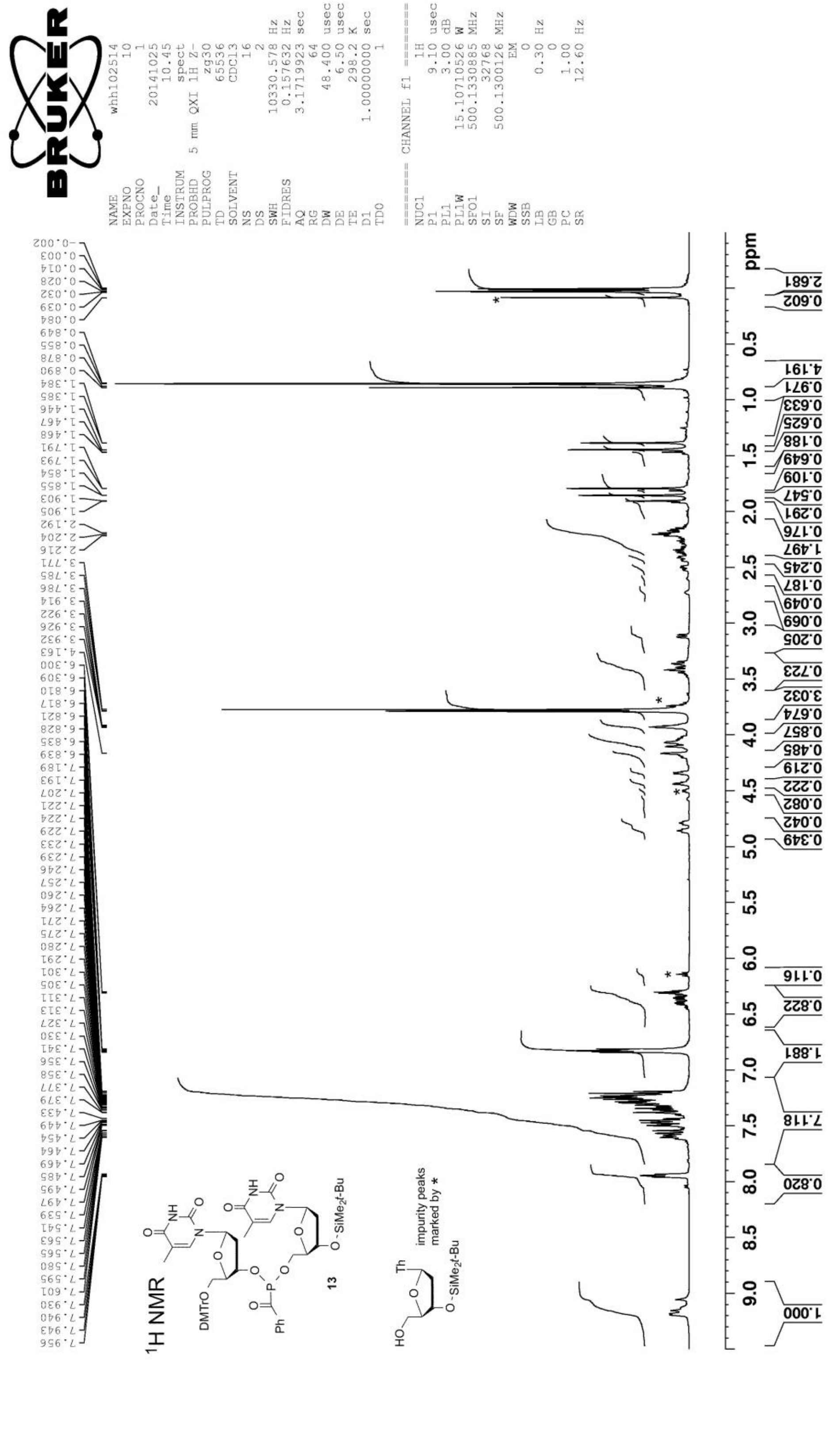
1H NMR
DMTrO
Ph
13
O-SiMe2t-Bu
HO
Th
impurity peaks marked by *
ppm
BRUKER
NAME whh102514
EXPNO 10
PROCNO 1
Date_ 20141025
Time 10.45
INSTRUM spect
PROBHD 5 mm QXI 1H Z-
PULPROG zg30
TD 65536
SOLVENT CDCl3
NS 16
DS 2
SWH 10330.578 Hz
FIDRES 0.157632 Hz
AQ 3.1719923 sec
RG 64
DW 48.400 usec
DE 6.50 usec
TE 298.2 K
D1 1.00000000 sec
TD0 1
======== CHANNEL f1 ========
NUC1 1H
P1 9.10 usec
PL1 3.00 dB
PL1W 15.10710526 W
SFO1 500.1330885 MHz
SI 32768
SF 500.1300126 MHz
WDW EM
SSB 0
LB 0.30 Hz
GB 0
PC 1.00
SR 12.60 Hz

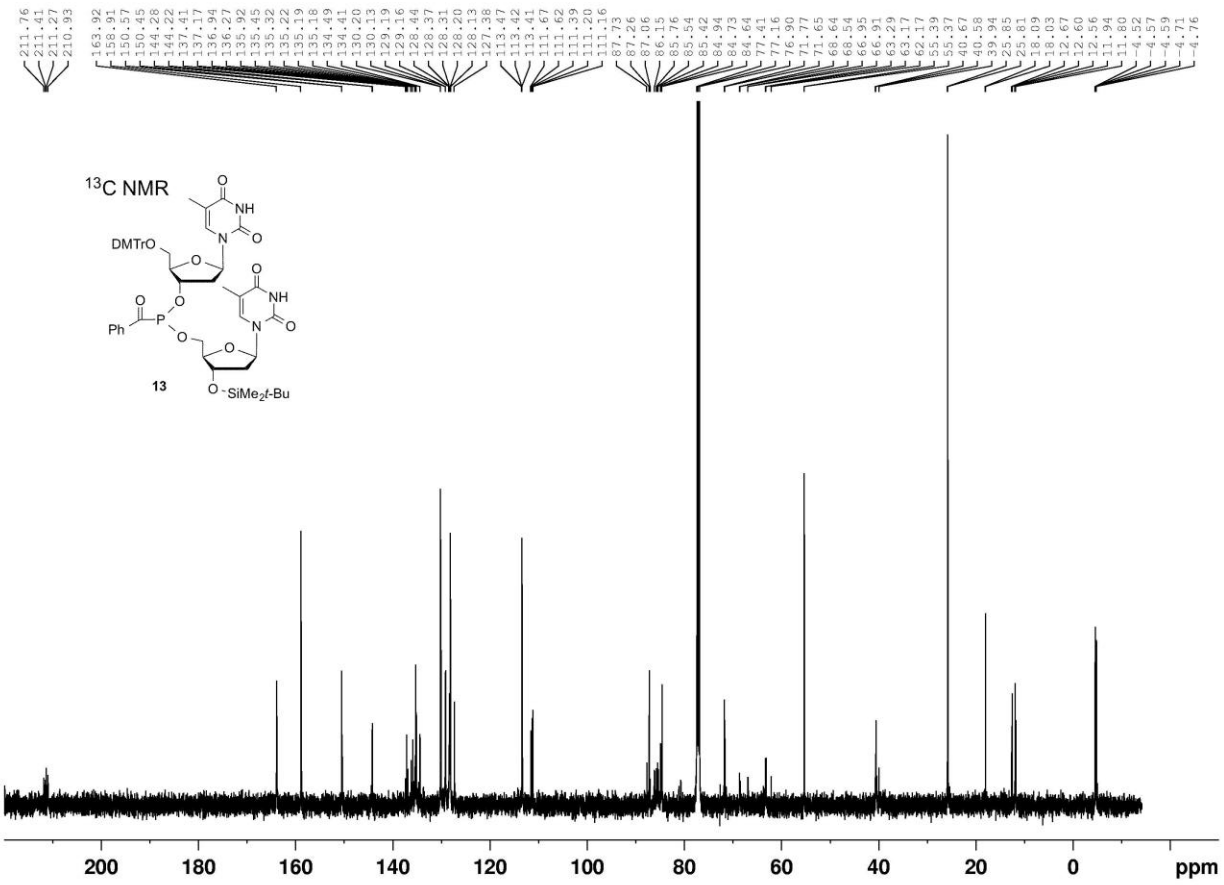

NAME	whh102514
EXPNO	13
PROCNO	1
Date_	20141025
Time	15.14
INSTRUM	spect
PROBHD	5 mm QXI 1H Z-
PULPROG	zgpg30
TD	65536
SOLVENT	CDCl3
NS	4096
DS	4
SWH	31250.000 Hz
FIDRES	0.476837 Hz
AQ	1.0486259 sec
RG	322
DW	16.000 usec
DE	6.50 usec
TE	298.2 K
D1	2.00000000 sec
D11	0.03000000 sec
TD0	1

======== CHANNEL f1 ========

NUC1	13C
P1	15.50 usec
PL1	-1.00 dB
PL1W	132.81565857 W
SFO1	125.7716224 MHz

======== CHANNEL f2 ========

CPDPRG2	waltz16
NUC2	1H
PCPD2	80.00 usec
PL2	3.00 dB
PL12	21.88 dB
PL13	120.00 dB
PL2W	15.10710526 W
PL12W	0.19551556 W
PL13W	0.00000000 W
SFO2	500.1320005 MHz
SI	32768
SF	125.7577747 MHz
WDW	EM
SSB	0
LB	1.00 Hz
GB	0
PC	1.40
SR	-14.31 Hz

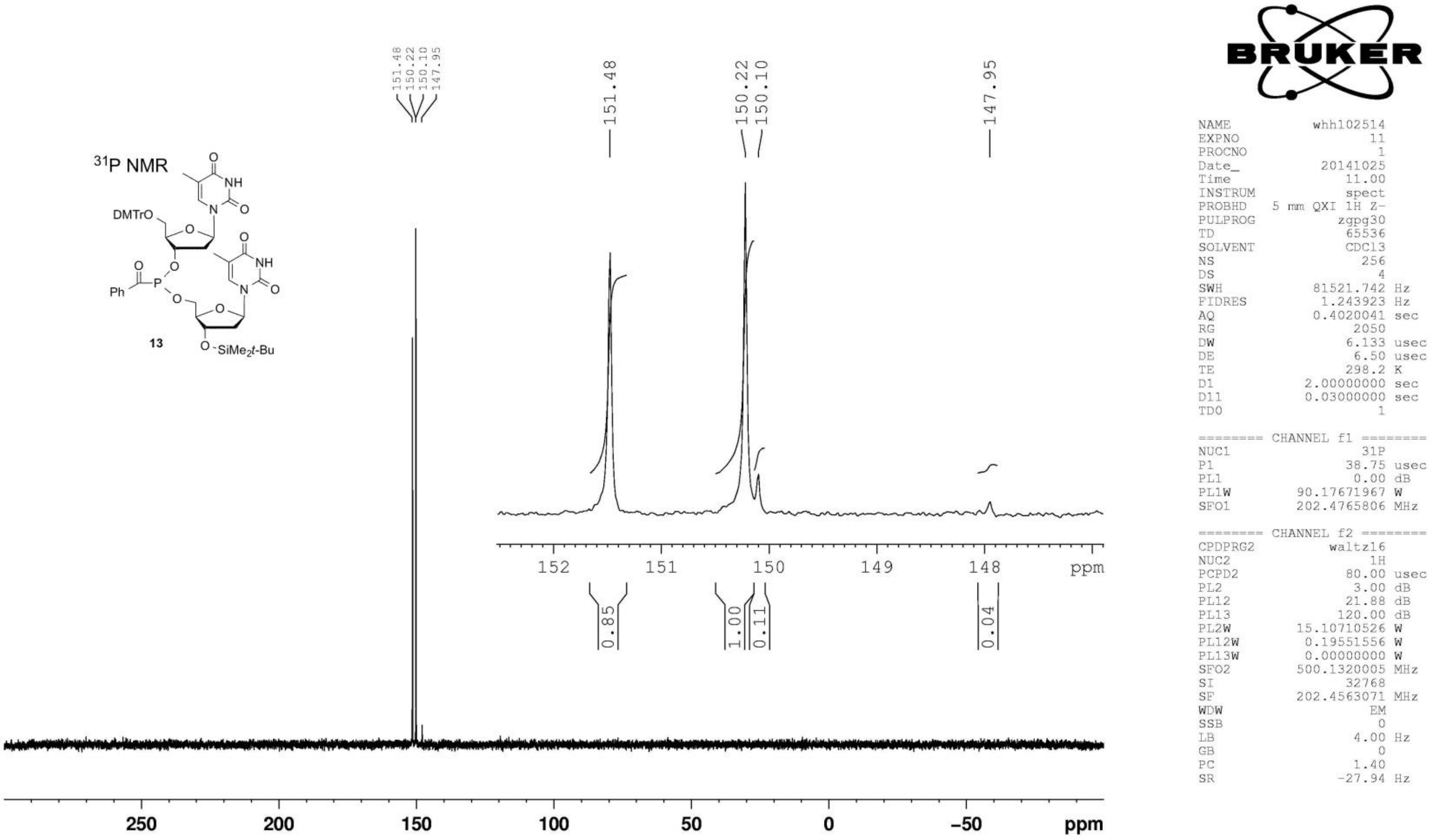

^{31}P NMR
DMTrO
Ph
13
O-SiMe$_2$t-Bu
151.48
150.22
150.10
147.95
0.85
1.00
0.11
0.04
152
151
150
149
148
ppm
250
200
150
100
50
0
–50
ppm
NAME whh102514
EXPNO 11
PROCNO 1
Date_ 20141025
Time 11.00
INSTRUM spect
PROBHD 5 mm QXI 1H Z-
PULPROG zgpg30
TD 65536
SOLVENT CDCl3
NS 256
DS 4
SWH 81521.742 Hz
FIDRES 1.243923 Hz
AQ 0.4020041 sec
RG 2050
DW 6.133 usec
DE 6.50 usec
TE 298.2 K
D1 2.00000000 sec
D11 0.03000000 sec
TD0 1
======== CHANNEL f1 ========
NUC1 31P
P1 38.75 usec
PL1 0.00 dB
PL1W 90.17671967 W
SFO1 202.4765806 MHz
======== CHANNEL f2 ========
CPDPRG2 waltz16
NUC2 1H
PCPD2 80.00 usec
PL2 3.00 dB
PL12 21.88 dB
PL13 120.00 dB
PL2W 15.10710526 W
PL12W 0.19551556 W
PL13W 0.00000000 W
SFO2 500.1320005 MHz
SI 32768
SF 202.4563071 MHz
WDW EM
SSB 0
LB 4.00 Hz
GB 0
PC 1.40
SR -27.94 Hz

COSY90

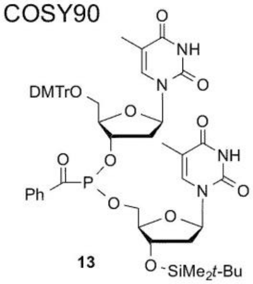

13

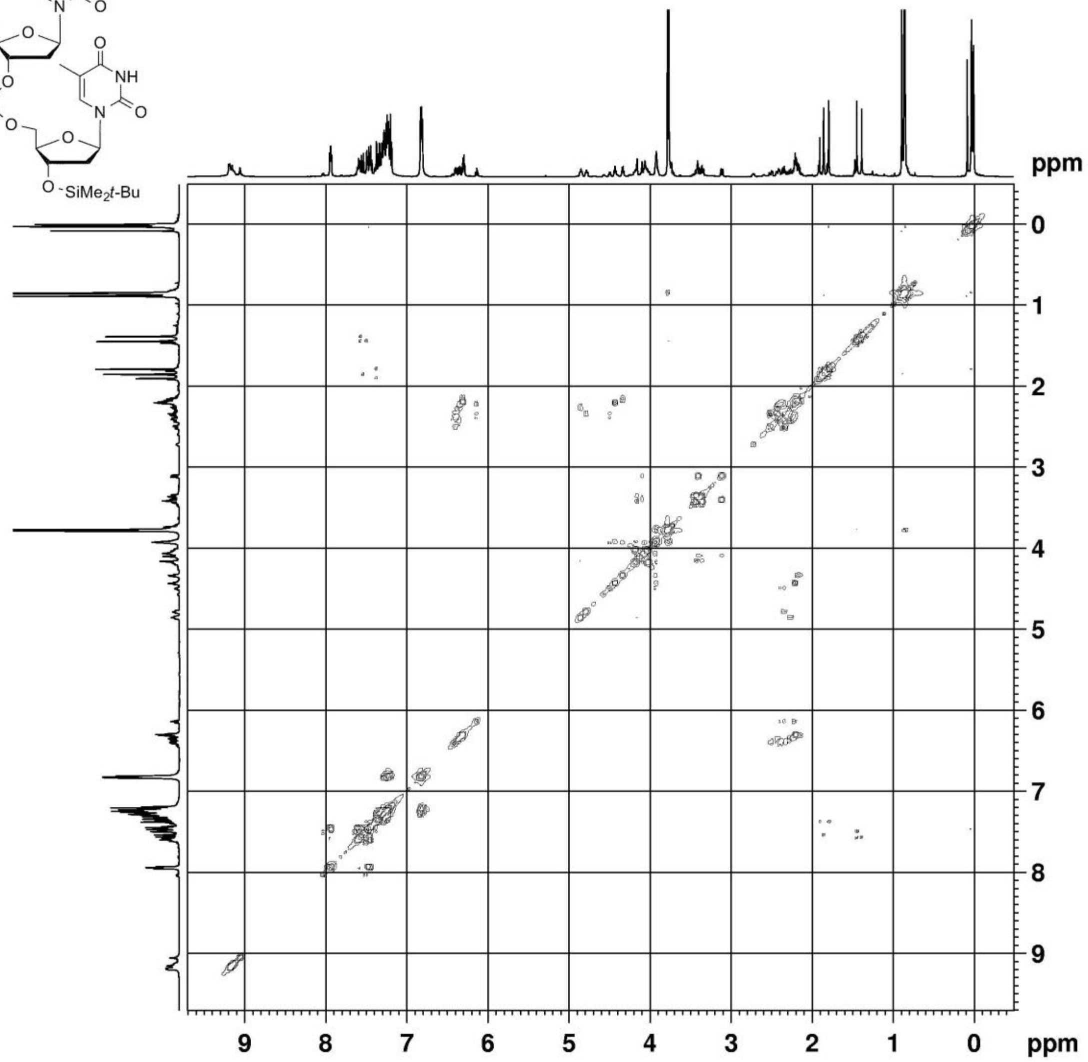

NAME	whh102514	
EXPNO	14	
PROCNO	1	
Date_	20141025	
Time	15.23	
INSTRUM	spect	
PROBHD	5 mm QXI 1H Z-	
PULPROG	cosyqf90	
TD	2048	
SOLVENT	CDCl3	
NS	8	
DS	4	
SWH	5154.639	Hz
FIDRES	2.516914	Hz
AQ	0.1987060	sec
RG	57	
DW	97.000	usec
DE	6.50	usec
TE	298.2	K
D0	0.00000300	sec
D1	2.00000000	sec
IN0	0.00019395	sec

======== CHANNEL f1 ========

NUC1	1H	
P1	9.10	usec
PL1	3.00	dB
PL1W	15.10710526	W
SFO1	500.1323164	MHz
ND0	1	
TD	256	
SFO1	500.1323	MHz
FIDRES	20.142307	Hz
SW	10.310	ppm
FnMODE	QF	
SI	1024	
SF	500.1300126	MHz
WDW	SINE	
SSB	0	
LB	0.00	Hz
GB	0	
PC	1.00	
SR	12.60	Hz
SI	1024	
MC2	QF	
SF	500.1300126	MHz
WDW	SINE	
SSB	0	
LB	0.00	Hz
GB	0	

HSQC

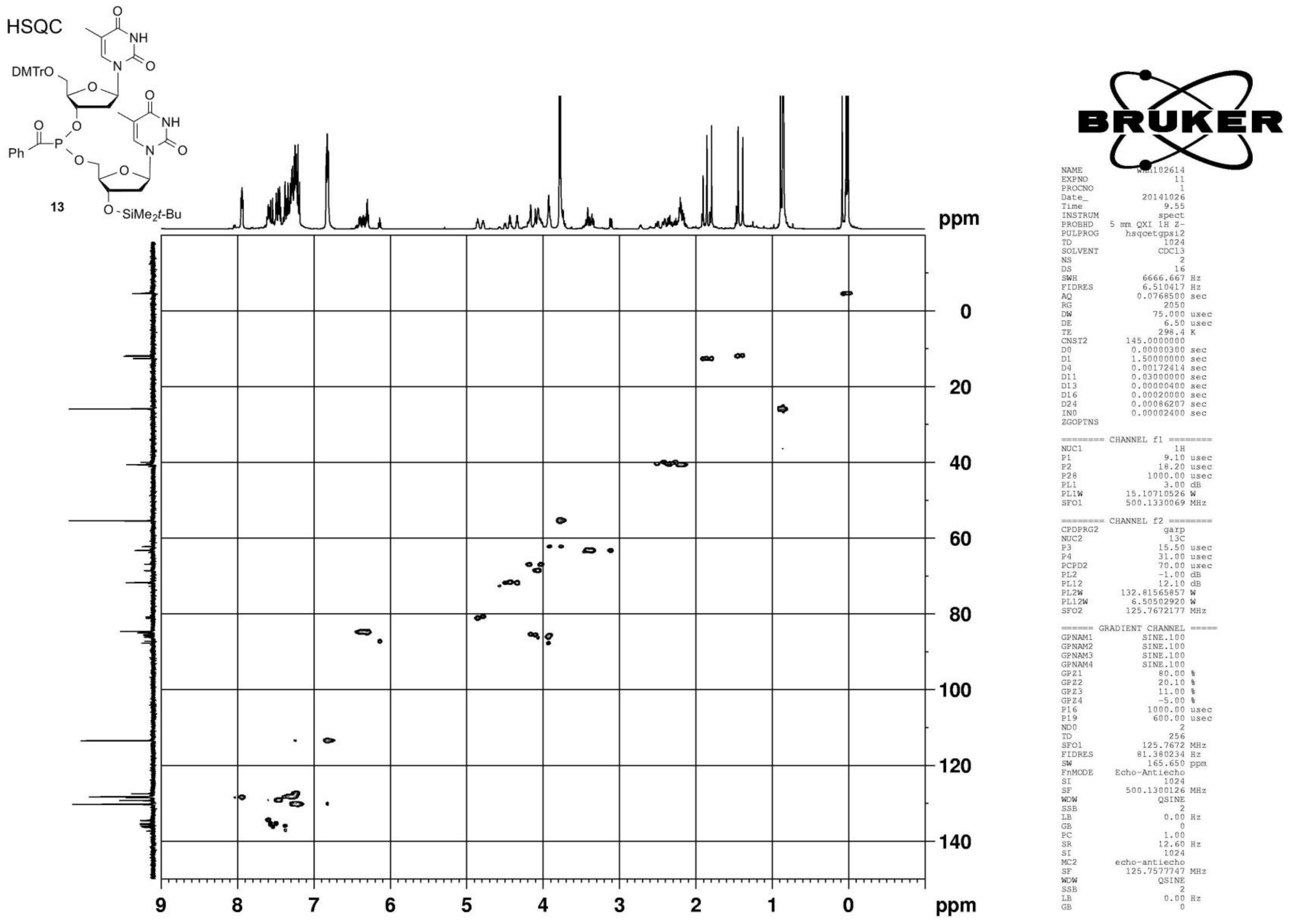
DMTrO
Ph
SiMe2t-Bu
ppm
0
20
40
60
80
100
120
140
ppm
9
8
7
6
5
4
3
2
1
0
BRUKER
NAME wm102614
EXPNO 11
PROCNO 1
Date_ 20141026
Time 9.55
INSTRUM spect
PROBHD 5 mm QXI 1H Z-
PULPROG hsqcetgpsi2
TD 1024
SOLVENT CDCl3
NS 2
DS 16
SWH 6666.667 Hz
FIDRES 6.510417 Hz
AQ 0.0768500 sec
RG 2050
DW 75.000 usec
DE 6.50 usec
TE 298.4 K
CNST2 145.000000
D0 0.00000300 sec
D1 1.50000000 sec
D4 0.00172414 sec
D11 0.03000000 sec
D13 0.00000400 sec
D16 0.00020000 sec
D24 0.00086207 sec
IN0 0.00002400 sec
ZGOPTNS
======== CHANNEL f1 ========
NUC1 1H
P1 9.10 usec
P2 18.20 usec
P28 1000.00 usec
PL1 3.00 dB
PL1W 15.10710526 W
SFO1 500.1330069 MHz
======== CHANNEL f2 ========
CPDPRG2 garp
NUC2 13C
P3 15.50 usec
P4 31.00 usec
PCPD2 70.00 usec
PL2 -1.00 dB
PL12 12.10 dB
PL2W 132.81565857 W
PL12W 6.50502920 W
SFO2 125.7672177 MHz
====== GRADIENT CHANNEL =====
GPNAM1 SINE.100
GPNAM2 SINE.100
GPNAM3 SINE.100
GPNAM4 SINE.100
GPZ1 80.00 %
GPZ2 20.10 %
GPZ3 11.00 %
GPZ4 -5.00 %
P16 1000.00 usec
P19 600.00 usec
ND0 2
TD 256
SFO1 125.7672 MHz
FIDRES 81.380234 Hz
SW 165.650 ppm
FnMODE Echo-Antiecho
SI 1024
SF 500.1300126 MHz
WDW QSINE
SSB 2
LB 0.00 Hz
GB 0
PC 1.00
SR 12.60 Hz
SI 1024
MC2 echo-antiecho
SF 125.7577747 MHz
WDW QSINE
SSB 2
LB 0.00 Hz
GB 0

13

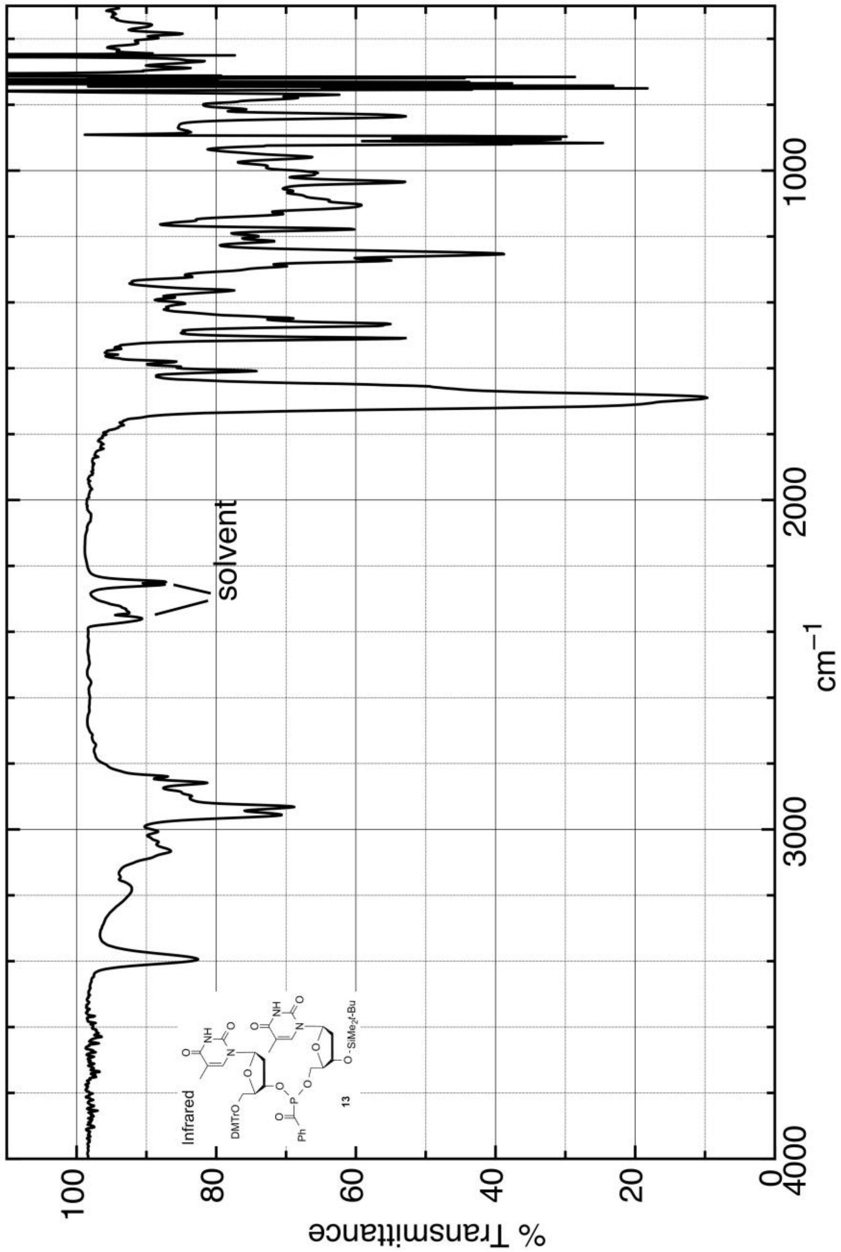

Infrared
DMTrO
Ph
13
O-SiMe2t-Bu
solvent
% Transmittance
cm−1
0
20
40
60
80
100
4000
3000
2000
1000

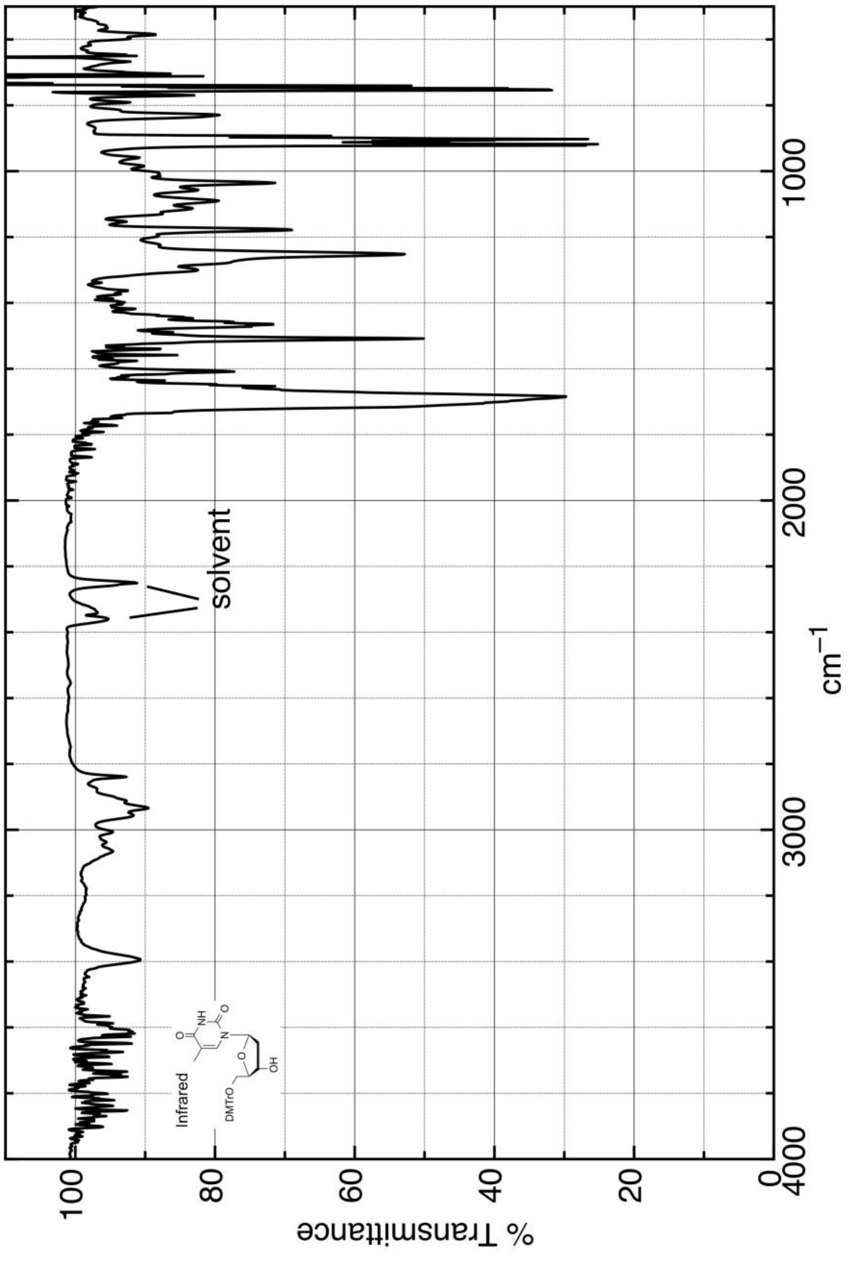
Infrared
DMTrO
NH
O
N
OH
solvent
% Transmittance
cm^{-1}
0
20
40
60
80
100
4000
3000
2000
1000

XYZ Coordinates for **7** from DFT optimization;
E(6-311G+(2d,p))= -1078.49563987 au

Atom	Atomic	Coordinates (Angstroms)		
Number	Number	X	Y	Z
1	15	-0.023368	0.169733	-0.826094
2	8	-0.734670	2.771713	-0.257079
3	7	1.421462	-0.241278	0.007741
4	7	-1.433560	-0.180628	0.076356
5	6	0.110745	2.055204	-0.770891
6	6	1.954595	0.457598	1.206204
7	1	1.265477	1.285959	1.401025
8	6	1.230538	2.668374	-1.604044
9	6	-2.595677	-0.749460	-0.660859
10	1	-3.379189	-0.882476	0.091785
11	6	-1.855706	-1.127351	2.350269
12	1	-2.792431	-1.634746	2.091102
13	1	-1.905181	-0.875026	3.416413
14	1	-1.036204	-1.837205	2.199139
15	6	1.979011	-0.427224	2.465267
16	1	0.992353	-0.847254	2.677949
17	1	2.295303	0.163317	3.333244
18	1	2.686572	-1.257599	2.360206
19	6	-2.779004	1.157441	1.746813
20	1	-2.595511	2.066033	1.170573
21	1	-2.825313	1.416214	2.811931
22	1	-3.758830	0.751448	1.469363
23	6	2.194976	-1.423811	-0.461701
24	1	3.064451	-1.485386	0.199956
25	6	-2.317140	-2.137115	-1.256476
26	1	-1.558304	-2.085880	-2.044680
27	1	-3.231916	-2.549515	-1.699533
28	1	-1.966114	-2.830066	-0.485131
29	6	-1.647562	0.143043	1.506400
30	1	-0.723446	0.615459	1.849028
31	6	3.342222	1.078660	0.960888
32	1	4.107956	0.313744	0.789077
33	1	3.652009	1.658062	1.838532
34	1	3.331079	1.748489	0.096710
35	6	-3.151826	0.212103	-1.724480
36	1	-3.361303	1.194693	-1.291130
37	1	-4.079940	-0.183996	-2.155062
38	1	-2.434737	0.346880	-2.542690
39	6	1.428934	-2.746677	-0.313775
40	1	1.066611	-2.878333	0.711023
41	1	2.081359	-3.593516	-0.559514

42	1	0.566770	-2.784276	-0.987567
43	6	2.733424	-1.246437	-1.889358
44	1	1.914131	-1.176311	-2.612892
45	1	3.360623	-2.100986	-2.170585
46	1	3.338972	-0.337066	-1.968068
47	1	0.787828	3.091280	-2.514708
48	1	1.690793	3.492920	-1.049588
49	1	1.993525	1.942694	-1.892081

XYZ Coordinates for **8** from DFT optimization;
E(6-311G+(2d,p))= -1231.17406507 au

Atom Number	Atomic Number	Coordinates (Angstroms) X	Y	Z
1	15	-0.029622	-0.290573	0.388031
2	7	0.331583	1.370506	0.127581
3	7	-1.665212	-0.645341	-0.027312
4	6	0.948391	-1.059218	-0.988680
5	6	0.515259	2.018791	-1.196116
6	1	0.261494	1.256220	-1.938670
7	6	-2.398183	-1.560601	0.891703
8	1	-3.421917	-1.602572	0.507199
9	6	-3.469202	1.043516	-0.454945
10	1	-4.234176	0.527592	0.137127
11	1	-3.986613	1.599798	-1.246116
12	1	-2.961397	1.759847	0.197151
13	6	-0.430970	3.213073	-1.412570
14	1	-1.476769	2.926987	-1.271244
15	1	-0.315832	3.602647	-2.431171
16	1	-0.209777	4.036209	-0.722980
17	6	-3.187529	-0.907068	-2.031617
18	1	-2.496339	-1.610535	-2.504175
19	1	-3.673304	-0.327311	-2.825358
20	1	-3.969733	-1.488157	-1.530399
21	6	0.680811	2.191272	1.319254
22	1	0.898673	3.191351	0.931461
23	6	-2.492367	-1.055986	2.341422
24	1	-1.508460	-1.038454	2.821093
25	1	-3.139745	-1.718740	2.928592
26	1	-2.912087	-0.045719	2.380395
27	6	-2.463810	0.047196	-1.062340
28	1	-1.749262	0.620925	-1.657532
29	6	1.974576	2.429447	-1.467459
30	1	2.313301	3.208932	-0.775176

31	1	2.065252	2.833771	-2.482869
32	1	2.642088	1.569402	-1.376100
33	6	-1.838643	-2.991325	0.836174
34	1	-1.861574	-3.379073	-0.187521
35	1	-2.429280	-3.662000	1.472369
36	1	-0.799797	-3.023443	1.184191
37	6	-0.490264	2.343202	2.300574
38	1	-1.375630	2.741327	1.794033
39	1	-0.221149	3.029831	3.112482
40	1	-0.757130	1.381892	2.752040
41	6	1.948122	1.702488	2.040345
42	1	1.791508	0.710440	2.477222
43	1	2.217343	2.390716	2.850957
44	1	2.793477	1.641553	1.347396
45	6	0.548557	-1.809192	-2.014184
46	1	1.263581	-2.292130	-2.674668
47	1	-0.505062	-1.966246	-2.201659
48	8	2.340169	-0.863668	-0.804673
49	6	3.029303	-1.764048	-0.040757
50	8	2.531789	-2.746786	0.458431
51	6	4.473258	-1.344323	0.080382
52	1	4.903194	-1.164968	-0.909633
53	1	4.538705	-0.406831	0.642977
54	1	5.036622	-2.120544	0.599067

XYZ Coordinates for **18** from DFT optimization;
Sum of electronic and thermal Free Energies(6-31G+(d))= -691.998977 au

Atom Number	Atomic Number	Coordinates (Angstroms) X	Y	Z
1	6	-2.395875	1.348909	-0.250561
2	6	-3.305990	0.289175	-0.329180
3	6	-2.852673	-1.023242	-0.180918
4	6	-1.494845	-1.272653	0.045765
5	6	-0.567901	-0.220820	0.125882
6	6	-1.041285	1.094246	-0.025389
7	15	1.215158	-0.658838	0.419399
8	6	3.553367	0.041777	-1.053137
9	6	2.026509	0.204764	-1.039799
10	6	1.656013	0.528174	1.795329
11	1	-2.742049	2.373614	-0.364742
12	1	-4.360560	0.487733	-0.503957
13	1	-3.552962	-1.853029	-0.239194
14	1	-1.147473	-2.296842	0.162938

15	1	-0.351959	1.933442	0.032885
16	1	3.984167	0.490486	-1.956763
17	1	3.841327	-1.016119	-1.034616
18	1	4.022421	0.530313	-0.190679
19	1	1.756573	1.269799	-1.057032
20	1	1.596510	-0.246131	-1.943274
21	1	2.720822	0.425393	2.032063
22	1	1.088554	0.264030	2.694096
23	1	1.453351	1.577912	1.549333

XYZ Coordinates for **18** transition state from DFT optimization;
Sum of electronic and thermal Free Energies(6-31G+(d))= -691.947172 au

Atom Number	Atomic Number	Coordinates (Angstroms) X	Y	Z
1	6	2.834870	0.855352	0.234687
2	6	3.291750	-0.457361	0.077929
3	6	2.356301	-1.472865	-0.150160
4	6	0.993569	-1.189413	-0.220144
5	6	0.521841	0.135172	-0.067570
6	6	1.474714	1.154192	0.164504
7	15	-1.211261	0.519598	-0.172081
8	6	-2.907505	-1.648502	0.691271
9	6	-2.448595	-0.806549	-0.510153
10	6	-1.747138	2.266628	0.029452
11	1	3.543851	1.660685	0.413915
12	1	4.352566	-0.684755	0.134037
13	1	2.688778	-2.501371	-0.272497
14	1	0.293032	-2.002527	-0.392960
15	1	1.151979	2.184737	0.289689
16	1	-3.630047	-2.411115	0.367984
17	1	-2.062127	-2.159287	1.164162
18	1	-3.384740	-1.022485	1.452597
19	1	-3.304314	-0.295947	-0.969505
20	1	-2.033203	-1.451322	-1.294621
21	1	-2.831058	2.284109	-0.127840
22	1	-1.545628	2.658341	1.032032
23	1	-1.289018	2.927039	-0.714681

XYZ Coordinates for **19** from DFT optimization;
Sum of electronic and thermal Free Energies(6-31G+(d))= -766.036587 au

Atom Number	Atomic Number	Coordinates (Angstroms) X	Y	Z
1	6	-2.332829	0.055021	1.437940
2	6	-3.290100	0.279684	0.442357
3	6	-2.916996	0.225074	-0.902225
4	6	-1.591192	-0.054336	-1.249886
5	6	-0.619296	-0.279242	-0.261478
6	6	-1.009530	-0.221243	1.088513
7	15	1.115680	-0.640195	-0.806029
8	6	2.067601	0.734962	0.082548
9	6	1.611924	-2.080081	0.258289
10	8	3.045519	0.467672	0.759838
11	6	1.636892	2.166326	-0.180619
12	1	-2.616854	0.097075	2.486675
13	1	-4.320150	0.494524	0.715388
14	1	-3.655643	0.394879	-1.681664
15	1	-1.307673	-0.102437	-2.298680
16	1	-0.278035	-0.389503	1.875496
17	1	2.673771	-2.281415	0.089696
18	1	1.036429	-2.959498	-0.048406
19	1	1.463815	-1.902480	1.327786
20	1	2.364152	2.854252	0.259292
21	1	0.646606	2.350259	0.252646
22	1	1.555374	2.348894	-1.259133

XYZ Coordinates for **19** transition state from DFT optimization;
Sum of electronic and thermal Free Energies(6-31G+(d))= -766.002998 au

Atom Number	Atomic Number	Coordinates (Angstroms) X	Y	Z
1	6	-2.989774	0.765051	-0.679087
2	6	-3.506502	-0.367112	-0.041367
3	6	-2.644195	-1.224481	0.647920
4	6	-1.277615	-0.945800	0.715989
5	6	-0.742794	0.176661	0.054693
6	6	-1.619274	1.026889	-0.648425
7	15	1.026577	0.550220	0.110624
8	6	2.354735	-0.621259	-0.172889
9	6	1.551401	2.259579	0.518064
10	8	3.522774	-0.254005	-0.063112
11	6	1.989009	-2.043502	-0.557687
12	1	-3.651756	1.436440	-1.220293

13	1	-4.572147	-0.576967	-0.078483
14	1	-3.037867	-2.100738	1.156927
15	1	-0.625946	-1.591314	1.297599
16	1	-1.227132	1.888269	-1.182403
17	1	2.644123	2.216647	0.576362
18	1	1.153176	2.581012	1.484519
19	1	1.270230	2.980497	-0.254727
20	1	2.297413	-2.719490	0.248935
21	1	2.557659	-2.313064	-1.454613
22	1	0.921460	-2.178716	-0.751557

XYZ Coordinates for **20** from DFT optimization;
Sum of electronic and thermal Free Energies(6-31G+(d))= -936.138233 au

Atom Number	Atomic Number	Coordinates (Angstroms) X	Y	Z
1	6	-2.054739	0.044486	-0.300289
2	6	-2.940209	-0.973618	0.436752
3	6	-4.354482	-0.822392	0.088488
4	7	-5.473514	-0.692587	-0.196166
5	8	-0.715862	-0.157069	0.161113
6	15	0.549743	0.223342	-0.890229
7	6	2.194851	-1.760140	-0.121028
8	8	1.762588	-0.374544	0.055362
9	8	0.768444	1.827666	-0.628002
10	6	0.941617	2.429625	0.686626
11	1	-2.376141	1.066377	-0.070372
12	6	3.679172	-1.744192	-0.465997
13	1	1.632212	-2.195611	-0.958582
14	6	1.879371	-2.532016	1.155251
15	1	-2.124125	-0.106865	-1.384949
16	1	-2.830468	-0.843861	1.519349
17	1	-2.623123	-1.992938	0.190102
18	6	0.911972	3.938642	0.525724
19	1	1.897357	2.090180	1.097187
20	1	0.135627	2.082099	1.340833
21	1	2.189290	-3.578210	1.047850
22	1	4.046169	-2.767518	-0.606859
23	1	0.806554	-2.509073	1.370993
24	1	4.255394	-1.279126	0.342726
25	1	3.858506	-1.182683	-1.389140
26	1	2.416489	-2.099480	2.007732
27	1	1.048171	4.414954	1.503946
28	1	-0.046243	4.270960	0.111374
29	1	1.716107	4.277961	-0.136549

XYZ Coordinates for **20** after rotation to opposite configuration, from DFT optimization; Sum of electronic and thermal Free Energies(6-31G+(d))= -936.137960 au

Atom Number	Atomic Number	Coordinates (Angstroms) X	Y	Z
1	6	2.038365	-0.332346	-0.240296
2	6	3.087921	0.468896	0.548525
3	6	4.451315	0.020820	0.257507
4	7	5.528094	-0.343814	0.017209
5	8	0.752412	0.148930	0.158980
6	15	-0.469082	0.263299	-0.999639
7	6	-1.769137	-1.737475	0.390085
8	8	-1.269116	-1.154859	-0.863489
9	8	-1.476091	1.214423	-0.089394
10	6	-1.168394	2.624497	0.038825
11	1	2.118076	-1.399170	-0.005565
12	6	-3.291884	-1.751465	0.329960
13	1	-1.435138	-1.103132	1.216732
14	6	-1.160049	-3.127471	0.525561
15	1	2.191481	-0.200461	-1.318705
16	1	2.905330	0.360018	1.623658
17	1	3.006766	1.533670	0.303516
18	6	-2.384660	3.333245	0.607938
19	1	-0.904939	3.031720	-0.946485
20	1	-0.302105	2.736384	0.700578
21	1	-1.508658	-3.600495	1.451239
22	1	-3.699301	-2.185614	1.250883
23	1	-0.066781	-3.075602	0.557092
24	1	-3.638513	-2.355275	-0.517218
25	1	-3.686643	-0.736653	0.221907
26	1	-1.455285	-3.762723	-0.317953
27	1	-2.166838	4.401092	0.726783
28	1	-2.649296	2.924937	1.589570
29	1	-3.247082	3.228355	-0.059309

XYZ Coordinates for **20** transition state from DFT optimization;
Sum of electronic and thermal Free Energies(6-31G+(d))= -936.072122 au

Atom Number	Atomic Number	Coordinates (Angstroms) X	Y	Z
1	6	2.370958	-0.332605	0.000023
2	6	3.536484	-1.341651	0.000325
3	6	4.840569	-0.675170	0.000196
4	7	5.865295	-0.126270	0.000115
5	8	1.155797	-1.069680	0.000426

6	15	-0.536825	-0.545341	0.000407
7	6	-3.223424	-0.973199	0.000676
8	8	-2.184035	0.022650	0.000989
9	8	0.000101	0.997639	-0.000165
10	6	-0.767950	2.228594	-0.000602
11	1	2.423502	0.309689	-0.886804
12	6	-4.053260	-0.816593	1.273541
13	1	-2.752368	-1.970932	0.002711
14	6	-4.049600	-0.820113	-1.274994
15	1	2.423697	0.310476	0.886274
16	1	3.472559	-1.986210	-0.883712
17	1	3.472473	-1.985763	0.884681
18	6	0.213817	3.386153	-0.000764
19	1	-1.409293	2.237631	0.884513
20	1	-1.409090	2.237196	-0.885869
21	1	-4.840708	-1.578746	-1.309858
22	1	-4.845200	-1.574380	1.307758
23	1	-3.417420	-0.940361	-2.161764
24	1	-4.521580	0.174390	1.310023
25	1	-3.423811	-0.935455	2.162437
26	1	-4.518758	0.170334	-1.315103
27	1	-0.343179	4.330532	-0.003147
28	1	0.853411	3.362735	-0.889739
29	1	0.850608	3.365511	0.890284

XYZ Coordinates for **21** from DFT optimization;
Sum of electronic and thermal Free Energies(6-31G+(d))= -842.668118 au

Atom Number	Atomic Number	Coordinates (Angstroms) X	Y	Z
1	6	0.417580	2.965013	0.584131
2	8	-1.854501	2.326648	0.122276
3	1	-0.021325	3.905237	0.930003
4	6	-0.671930	2.036281	0.104336
5	15	-0.234200	0.359015	-0.690536
6	6	2.076795	-0.948312	-0.244079
7	8	1.188423	0.141491	0.130612
8	8	-1.273562	-0.595438	0.194031
9	6	-2.430370	-1.164635	-0.454690
10	1	0.984150	2.484599	1.390053
11	6	3.467341	-0.359128	-0.453263
12	1	1.717047	-1.377673	-1.191180
13	6	2.031773	-2.012664	0.848426
14	1	1.132742	3.159813	-0.225472

15	6	-2.878663	-2.378034	0.342805
16	1	-2.176721	-1.441602	-1.487980
17	1	-3.219776	-0.404858	-0.488009
18	1	2.684174	-2.855288	0.587872
19	1	4.178706	-1.146032	-0.731236
20	1	1.012475	-2.386666	0.984270
21	1	3.822519	0.116982	0.468183
22	1	3.456692	0.393111	-1.249421
23	1	2.372610	-1.593265	1.802190
24	1	-3.778320	-2.812063	-0.110710
25	1	-3.113867	-2.095651	1.374481
26	1	-2.096144	-3.144612	0.363668

XYZ Coordinates for **21** after rotation to opposite configuration, from DFT optimization; Sum of electronic and thermal Free Energies(6-31G+(d))= -842.668984 au

Atom Number	Atomic Number	Coordinates (Angstroms) X	Y	Z
1	6	-0.203875	-2.883460	0.605711
2	8	-2.288031	-1.829577	0.045501
3	1	-0.833309	-3.694144	0.983372
4	6	-1.071232	-1.791765	0.019087
5	15	-0.207857	-0.382810	-0.949807
6	6	2.258944	0.466609	-0.258749
7	8	1.035201	-0.200323	0.157097
8	8	-1.226791	0.881889	-0.693503
9	6	-1.755834	1.274144	0.598913
10	1	0.489900	-3.270217	-0.151996
11	6	2.209174	1.936934	0.149920
12	1	2.330849	0.394450	-1.354175
13	6	3.421097	-0.287633	0.377602
14	1	0.412596	-2.469757	1.412216
15	6	-2.491640	2.591130	0.423588
16	1	-0.923033	1.374861	1.304831
17	1	-2.430248	0.488949	0.952252
18	1	4.375615	0.169691	0.091203
19	1	3.130944	2.448328	-0.153256
20	1	3.430087	-1.334494	0.056135
21	1	2.106926	2.028068	1.237886
22	1	1.363926	2.447995	-0.322829
23	1	3.341383	-0.262169	1.470843
24	1	-2.915317	2.911199	1.383559
25	1	-3.309599	2.482887	-0.296466
26	1	-1.815692	3.374834	0.064331

XYZ Coordinates for **21** transition state from DFT optimization;
Sum of electronic and thermal Free Energies(6-31G+(d))= -842.601643 au

Atom Number	Atomic Number	Coordinates (Angstroms) X	Y	Z
1	6	-0.495296	3.064340	0.146405
2	8	1.842796	2.421002	0.257126
3	1	-0.537962	3.485847	1.157291
4	6	0.675162	2.104010	0.067021
5	15	0.248203	0.420344	-0.364057
6	6	-2.065146	-0.832641	0.334162
7	8	-1.265434	-0.115225	-0.672690
8	8	1.433060	-0.680735	-0.578212
9	6	2.034054	-1.350955	0.570492
10	1	-0.312104	3.884990	-0.556750
11	6	-3.476388	-0.266813	0.253700
12	1	-1.625936	-0.618516	1.317330
13	6	-1.997499	-2.325226	0.036178
14	1	-1.450141	2.590396	-0.095224
15	6	3.222689	-2.146085	0.065840
16	1	1.280585	-2.002751	1.030266
17	1	2.337103	-0.590808	1.297488
18	1	-0.966589	-2.692094	0.080269
19	1	-4.130162	-0.777711	0.970548
20	1	-2.392330	-2.533157	-0.964714
21	1	-3.888864	-0.407929	-0.751758
22	1	-3.484175	0.803656	0.484008
23	1	-2.592642	-2.885608	0.767380
24	1	3.700070	-2.669410	0.903321
25	1	3.961262	-1.482838	-0.395788
26	1	2.910014	-2.889352	-0.675379
